# A causal examination of the correlation between hormonal and reproductive factors and low back pain

**DOI:** 10.3389/fendo.2024.1326761

**Published:** 2024-05-10

**Authors:** Dafu Chen, Jiaxiang Zhou, Chengkai Lin, Junhong Li, Zhengya Zhu, Xuezhi Rao, Jianmin Wang, Jianfeng Li, Hongkun Chen, Fuan Wang, Xianlong Li, Manman Gao, Zhiyu Zhou, Yongming Xi, Shufen Li

**Affiliations:** ^1^ Laboratory of Bone Tissue Engineering, Beijing Laboratory of Biomedical Materials, National Center for Orthopaedics, Beijing Research Institute of Traumatology and Orthopaedics, Beijing Jishuitan Hospital, Capital Medical University, Beijing, China; ^2^ Department of Spinal Surgery, The Affiliated Hospital of Qingdao University, Qingdao, China; ^3^ Innovation Platform of Regeneration and Repair of Spinal Cord and Nerve Injury, Department of Orthopaedic Surgery, The Seventh Affiliated Hospital of Sun Yat-sen University, Shenzhen, China; ^4^ Beijing University of Chinese Medicine, Beijing, China; ^5^ Department of Sport Medicine, Institute of Translational Medicine, The First Affiliated Hospital of Shenzhen University, Shenzhen Second People’s Hospital, Shenzhen, China; ^6^ Guangdong Provincial Key Laboratory of Orthopedics and Traumatology, The First Affiliated Hospital of Sun Yat-sen University, Guangzho, China; ^7^ Shenzhen Key Laboratory of Anti-aging and Regenerative Medicine, Department of Medical Cell Biology and Genetics, Health Sciences Center, Shenzhen University, Shenzhen, China; ^8^ Changzhou Maternal and Child Health Care Hospital, Changzhou Medical Center, Nanjing Medical University, Changzhou, China

**Keywords:** low back pain, reproductive factors, age at menarche, age at menopause, age at first birth, Mendelian randomization

## Abstract

**Background:**

The relationship between hormonal fluctuations in the reproductive system and the occurrence of low back pain (LBP) has been widely observed. However, the causal impact of specific variables that may be indicative of hormonal and reproductive factors, such as age at menopause (ANM), age at menarche (AAM), length of menstrual cycle (LMC), age at first birth (AFB), age at last live birth (ALB) and age first had sexual intercourse (AFS) on low back pain remains unclear.

**Methods:**

This study employed Bidirectional Mendelian randomization (MR) using publicly available summary statistics from Genome Wide Association Studies (GWAS) and FinnGen Consortium to investigate the causal links between hormonal and reproductive factors on LBP. Various MR methodologies, including inverse-variance weighted (IVW), MR-Egger regression, and weighted median, were utilized. Sensitivity analysis was conducted to ensure the robustness and validity of the findings. Subsequently, Multivariate Mendelian randomization (MVMR) was employed to assess the direct causal impact of reproductive and hormone factors on the risk of LBP.

**Results:**

After implementing the Bonferroni correction and conducting rigorous quality control, the results from MR indicated a noteworthy association between a decreased risk of LBP and AAM (OR=0.784, 95% CI: 0.689-0.891; p=3.53E-04), AFB (OR=0.558, 95% CI: 0.436-0.715; p=8.97E-06), ALB (OR=0.396, 95% CI: 0.226-0.692; p=0.002), and AFS (OR=0.602, 95% CI: 0.518-0.700; p=3.47E-10). Moreover, in the reverse MR analysis, we observed no significant causal effects of LBP on ANM, AAM, LMC and AFS. MVMR analysis demonstrated the continued significance of the causal effect of AFB on LBP after adjusting for BMI.

**Conclusion:**

Our study explored the causal relationship between ANM, AAM, LMC, AFB, AFS, ALB and the prevalence of LBP. We found that early menarche, early age at first birth, early age at last live birth and early age first had sexual intercourse may decrease the risk of LBP. These insights enhance our understanding of LBP risk factors, offering valuable guidance for screening, prevention, and treatment strategies for at-risk women.

## Introduction

Low back pain (LBP) is a prevalent public health issue, affecting approximately 60-80% of individuals at various stages of their lives (1, 2). Intervertebral disc degeneration is a major contributing factor to LBP and a noticeable trend towards its occurrence at younger ages has been observed (3, 4). The prevalence of LBP is generally higher among women than men, which can be attributed to factors such as increased pain sensitivity, variations in the menstrual cycle, physiological responses to pregnancy and childbirth, and abdominal weight gain during the perimenopausal phase (5–11).

Some studies have found a higher propensity for LBP among postmenopausal women compared to men of equivalent age (12). There was also evidence of an increased likelihood of LBP in individuals undergoing postmenopausal hormone therapy (13, 14). However, conflicting perspectives exist, with some suggesting potential positive outcomes associated with hormone therapy (15–17). These divergent views highlighted the potential significance of hormonal and reproductive factors in the pathogenesis and progression of LBP.

A strong connection has been established between hormonal factors, such as age at menopause (ANM), age at menarche (AAM), length of menstrual cycle (LMC), and age at first birth (AFB), and the occurrence of LBP. Various studies have identified associations between these factors and the risk of developing LBP, although the causal relationship between these remains unclear (18–21).

Observational studies on this subject were prone to bias due to confounding factors and reverse causality. To overcome these limitations, researchers have proposed the use of Mendelian randomization (MR) analysis. MR is a genetic epidemiological approach that uses single nucleotide polymorphisms (SNPs) as instrumental variables (IVs) for risk factors, allowing for the assessment of potential causal effects of exposure on outcomes (22). This method is based on Mendel’s second law (23), asserting that alleles are randomly allocated during meiosis and are typically unaffected by environmental influences (22, 24).

Prior to this investigation, MR analyses had not been used to explore the causal relationship between hormonal and reproductive factors and LBP. Therefore, we conducted the MR analysis focusing on four female hormonal and reproductive factors (ANM, AAM, LMC, AFB, AFS and ALB), examining their associations with LBP. Subsequently, Multivariate Mendelian randomization (MVMR) was employed to assess the direct causal impact of reproductive and hormone factors on the risk of LBP. These findings may enhance our understanding of the hormonal and reproductive mechanisms underlying LBP, guiding future research towards developing potential therapeutic or preventative strategies.

## Materials and methods

### Study design and data sources

**Figure 1:**
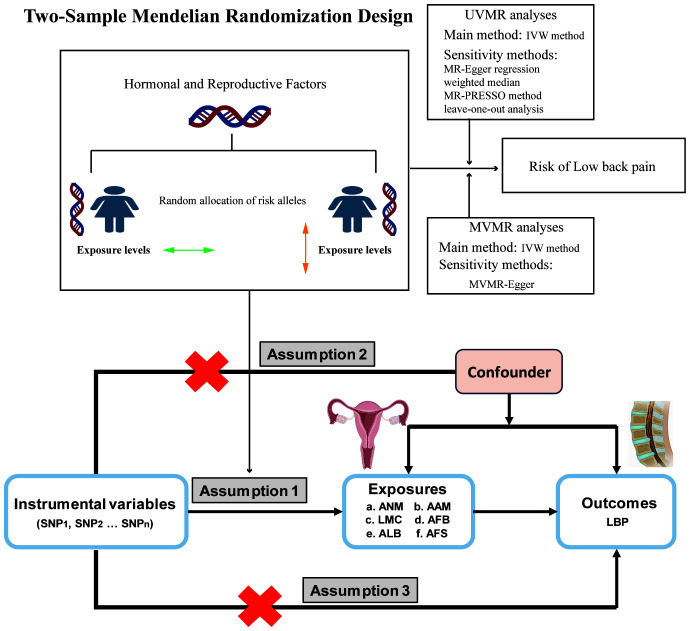
Scheme diagram of Mendelian randomization design.

We conducted a comprehensive analysis using publicly accessible Genome-Wide Association Studies (GWAS) database to explore the potential causal relationship between ANM, AAM, LMC, AFB, AFS and ALB, and the occurrence of LBP. A comprehensive overview of the proposed hypotheses was presented in (**Figure 1**). The current study adhered to the three fundamental assumptions essential for MR analyses (25): assumption 1, all chosen IVs exhibit a strong correlation with the exposure; assumption 2 the selected instrumental variables are independent of both exposure and outcome confounders; assumption 3, the selected instrumental variables impact the outcome solely through exposure. Previous MR studies have established BMI as a risk factor for LBP (26). And a reverse MR analysis was conducted to evaluate potential reverse causality. Consequently, we conducted MVMR to address this potential confounding factor. The exposure data were obtained from the GWAS database (https://gwas.mrcieu.ac.uk/). Data on LBP was sourced from the FinnGen Consortium (https://finngen.fi). The summary data for the GWAS of LBP from the FinnGen Consortium comprises 177,860 participants of European ancestry (13,178 cases and 164,682 controls). Summary information for all datasets were presented in (**Table 1**) . All participants were of European origin, and informed consent was obtained from each. Since our data were derived from publicly accessible GWAS summary statistics, no ethical approval was necessary.

**Table 1:** Summary of GWAS data for instrumental variables.

Analysis	Variable	ID	Sample size	Number of SNPs	Consortium	Population	Sex	Year
original analysis + validation analysis	ANM	ukb-b-17422	143,819	9,851,867	MRC-IEU	European	Males and Females	2018
	AAM	ukb-b-3768	243,944	9,851,867	MRC-IEU	European	Males and Females	2018
	LMC	ukb-a-351	30,245	10,894,596	Neale Lab	European	Males and Females	2017
	AFB	ukb-b-12405	170,498	9,851,867	MRC-IEU	European	Males and Females	2018
	ALB	ukb-b-8727	170,248	9,851,867	MRC-IEU	European	Males and Females	2018
	AFS	ukb-b-6591	406,457	9,851,867	MRC-IEU	European	Males and Females	2018
	BMI	ieu-b-40	681,275	2,336,260	GIANT	European	Males and Females	2018
original analysis	LBP	finn-b-M13_LOWBACKPAIN	13,178	16,380,287	NA	European	Males and Females	2021
validation analysis	LBP	ukb-d-M13_LOWBACKPAIN	361,194	12,184,069	NA	European	Males and Females	2018

ANM, age at menopause; AAM, age at menarche; LMC, length of menstrual cycle; AFB, age at first birth; ALB, Age at last live birth; AFS, Age first had sexual intercourse; BMI, Body mass index; LBP, low back pain.

### Selection of instrumental variables

Firstly, we carefully selected SNPs that demonstrated a strong association with exposure (P < 5 × 10^-8^) and excluded SNPs with F-values < 10, ensuring significance and mitigating weak instrumental variable bias (27). Secondly, we utilized specific parameters (r^2^ < 0.001, kb = 10,000 kb) to eliminate strong linkage disequilibrium, thus guaranteeing instrumental variable independence (28). Thirdly, we excluded SNPs associated with confounders and results using Phenoscanner V2. Additionally, palindromic SNPs with moderate allele frequencies were subsequently removed. Ultimately, we assessed the instrument strength through the F parameter, calculated using the formula F = R² × (n - 2)/(1 - R²), where R² represents the proportion of variance in instruments. The formula for R² is given by R² = 2 × effect allele frequency × (1 - effect allele frequency) × (Beta/SD², with SD equaling 1), and n denotes the sample size. An F statistic exceeding 10 indicated a diminished likelihood of weak instrument bias.

### Statistical analysis

In MR and MVMR analyses, the primary method employed was inverse variance weighting (IVW), complemented by MR-Egger, weighted median, simple mode, and weighted mode (29). In the absence of weak IVs, the primary outcome was determined using the IVW method, with the alternative methods considered as secondary outcomes. We employed MVMR as a statistical approach to incorporate SNP-phenotype associations into the analysis, facilitating the estimation of each phenotype’s direct impact on the outcome. As indicated by previous studies (26), in MVMR, we adjusted for body mass index (BMI) to clarify the causal impact of hormonal and reproductive factors on LBP.

### Heterogeneity and sensitivity test

Cochrane’s Q-test was utilized to detect heterogeneity, while funnel plots indicated heterogeneity through symmetry (30). The MR-Egger intercept test and the MR polytomous residuals and outliers (MR-PRESSO) global test were employed to assess pleiotropy (31). If significant pleiotropy was identified through the MR-PRESSO method, we will mitigate this concern by addressing outlier variability and subsequently reiterating the MR analysis. Lastly, the leave-one-out test was conducted to evaluate the sensitivity of the results. We utilized the TwoSample MR, MVMR, and MR-PRESSO packages in R software (version 4.3.1). Statistically significant associations were defined by results with a *p*-value < 0.05.

## Results

### Instrumental variables selection

After conducting a comprehensive quality assessment, we incorporated SNPs as reliable IVs for ANM, AAM, LMC, AFB, AFS, ALB and BMI. Detailed information regarding these IVs were provided in **Supplementary Tables S1**-**S9**. Notably, all the selected SNPs utilized as IVs possess F values exceeding 10, indicating their effectiveness as IVs.

### MR analysis of each feature related to hormonal and reproductive factors on LBP

**Figure 2:**
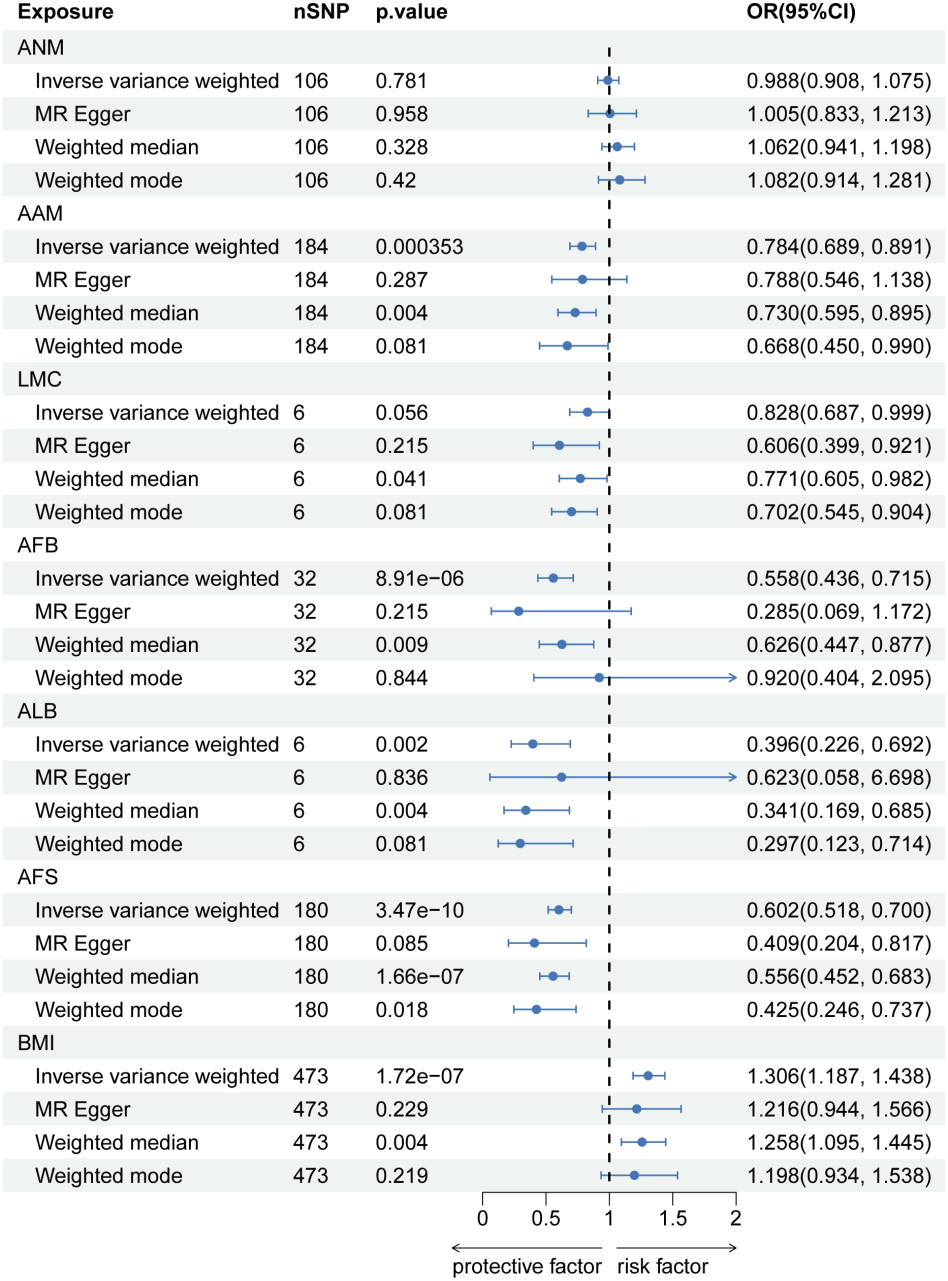
The causal relationship of genetically predicted Hormonal and Reproductive Factors and LBP.

After implementing the Bonferroni correction, the results from MR indicated a noteworthy association between a decreased risk of LBP and AAM (OR=0.784, 95% CI: 0.689-0.891; p=3.53E-04), AFB (OR=0.558, 95% CI: 0.436-0.715; p=8.97E-06), ALB (OR=0.396, 95% CI: 0.226-0.692; p=0.002), and AFS (OR=0.602, 95% CI: 0.518-0.700; p=3.47E-10). Nevertheless, no significant association was observed between ANM (OR=0.988, 95% CI: 0.908-0.1.075; p=0.781) and LMC (OR=0.828, 95% CI: 0.687-0.999; p=0.056) with LBP. The causal association between genetically predicted reproductive and hormonal factors and the risk of LBP were presented in **Figure 2**. Scatter plots and funnel plots illustrating the association between reproductive and hormonal factors and LBP were presented in **Supplementary Figures S1** and **S2**. Heterogeneity and pleiotropy are depicted in **Table 2**. The leave-one-out plot reinforces the robustness of our results, indicating that the influence of any individual SNP is unlikely to affect the causal estimate (**Supplementary Figure S3**).

**Table 2:** Sensitivity analysis of hormonal and reproductive factors causally linked to LBP.

Exposure	Outcome	Pleiotropy		Heterogeneity	
		Horizontal pleiotropy (Egger intercept)	Horizontal pleiotropy (*p*-value)	Heterogeneity (*Q*)	Heterogeneity (*p*-value)
ANM	LBP	-0.001	0.843	126.846	0.072
AAM		-1.13E-04	0.975	213.279	0.062
LMC		0.022	0.177	4.209	0.520
AFB		0.016	0.351	36.219	0.238
ALB		-0.011	0.719	3.011	0.698
AFS		0.007	0.262	215.809	0.031

ANM, age at menopause; AAM, age at menarche; LMC, length of menstrual cycle; AFB, age at first birth; ALB, Age at last live birth; AFS, Age first had sexual intercourse; BMI, Body mass index; LBP, low back pain.

**Figure 3:**
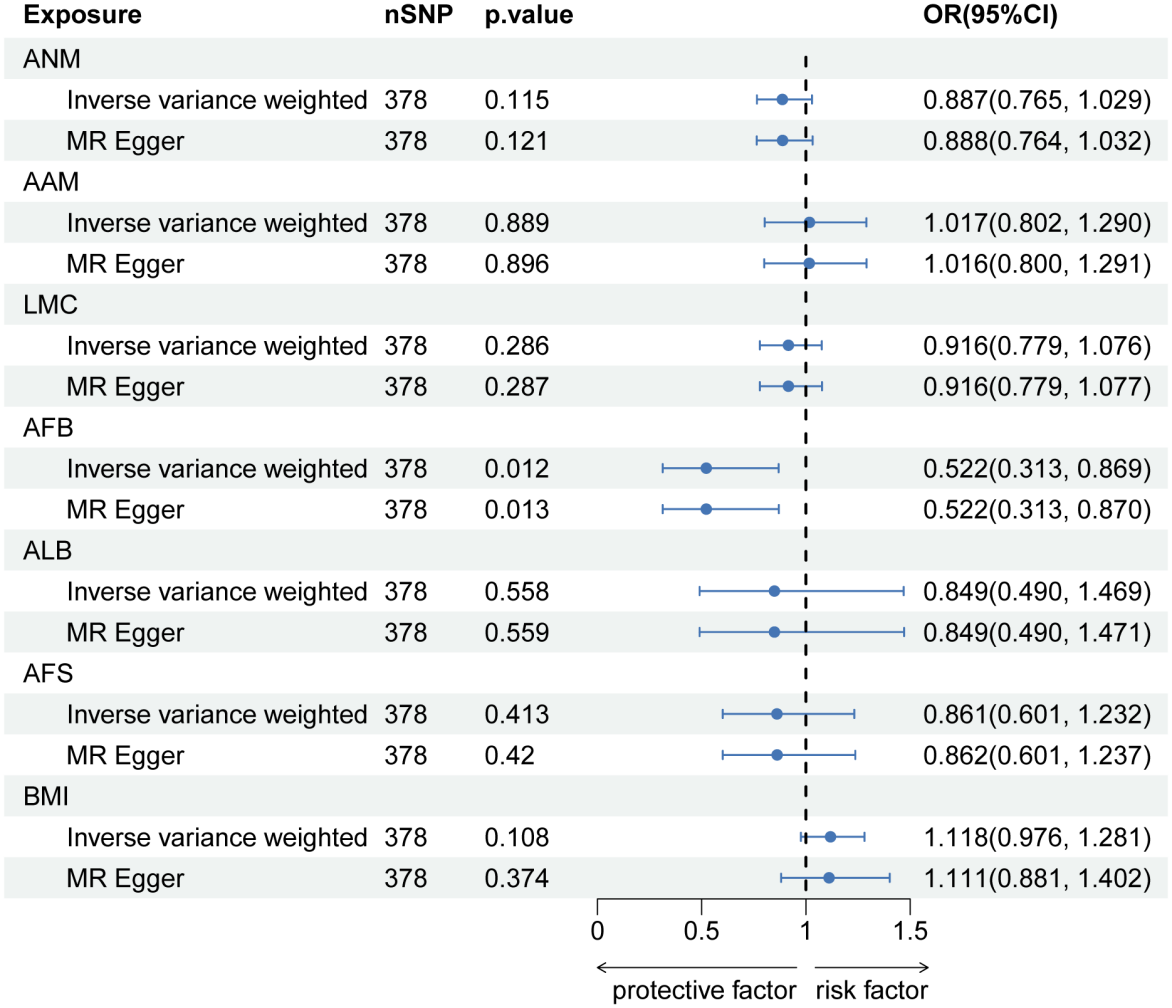
Causal estimates of Hormonal and Reproductive Factors on LBP in MVMR.

In MVMR analysis adjusting for BMI, AFB (OR=0.522, 95% CI: 0.313-0.869; p=0.012) exhibited a significant association with LBP. The MR-Lasso test results remained unaffected by the removal of heterogeneous SNPs. Nevertheless, associations between AAM, ALB, and AFS with LBP did not persist after further adjustment for BMI. Detailed MVMR results are presented in **Figure 3**.

### MR analysis of LBP on each feature related to hormonal and reproductive factors

**Figure 4:**
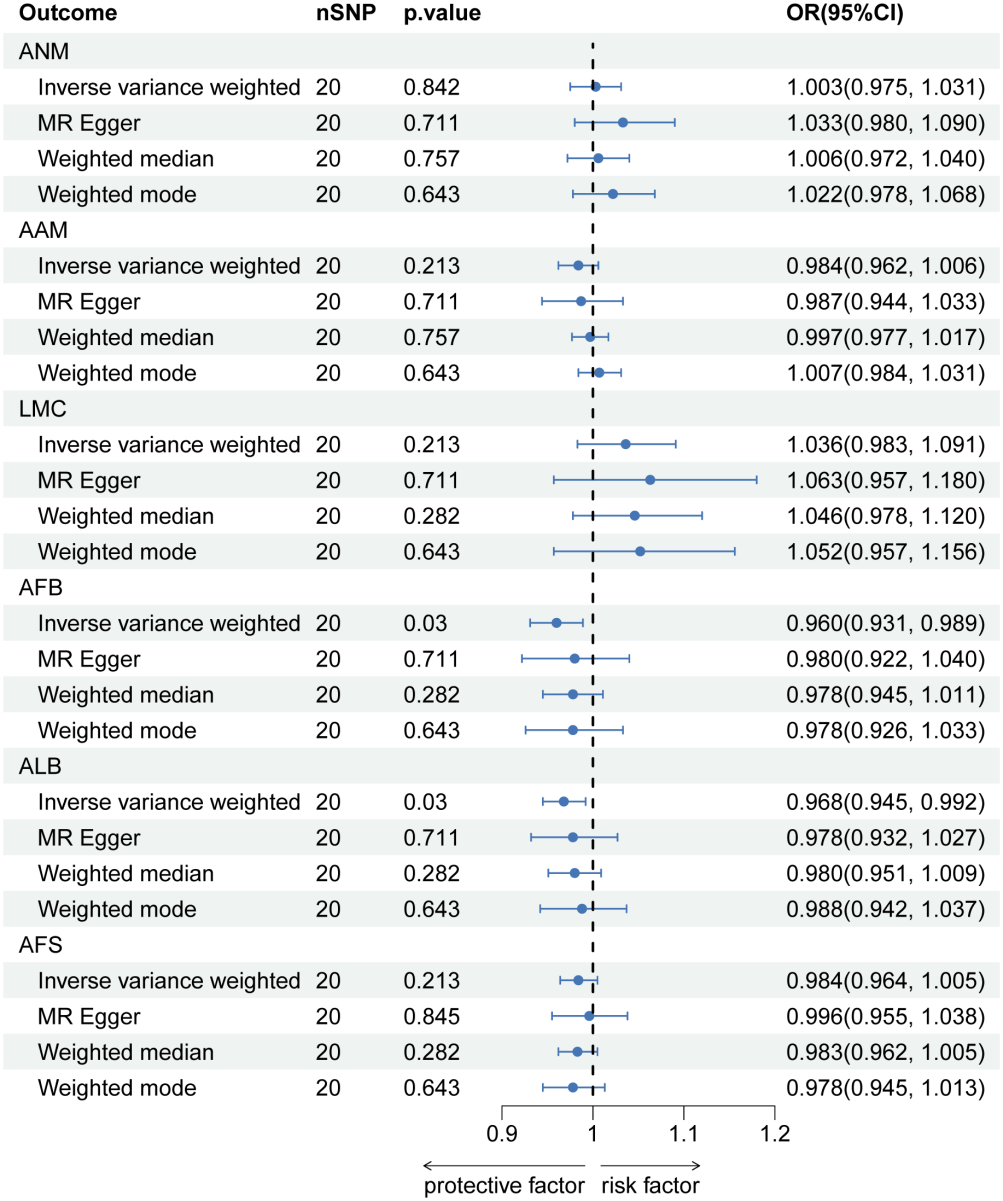
The causal relationship of genetically predicted LBP and Hormonal and Reproductive Factors.

In the reverse MR analysis, there is a causal negative relationship between LBP and AFB (OR=0.960, 95% CI: 0.931-0.989; p=0.030), as well as ALB (OR=0.968, 95% CI: 0.945-0.992; p=0.030). And no causal relationship was found between LBP and ANM (OR=1.003, 95% CI: 0.975-1.031; p=0.842), AAM (OR=0.984, 95% CI: 0.962-1.006; p=0.213), LMC (OR=1.036, 95% CI: 0.983-0.962; p=0.213) and AFS (OR=0.984, 95% CI: 0.964-1.005; p=0.213) (**Figure 4**). Information on pleiotropy and heterogeneity is referred to in **Table 3**. In addition, funnel plots, scatter plots and leave-one-out plots are shown in the **Supplementary Figures S4**-**S6**.

**Table 3:** Sensitivity analysis of LBP causally linked to hormonal and reproductive factors.

Exposure	Outcome	Pleiotropy		Heterogeneity	
		Horizontal pleiotropy (Egger intercept)	Horizontal pleiotropy (*p*-value)	Heterogeneity (*Q*)	Heterogeneity (*p*-value)
LBP	ANM	-0.004	0.217	28.511	0.074
	AAM	-4.38E-04	0.855	59.342	4.92E-06
	LMC	-0.003	0.582	25.735	0.138
	AFB	-0.002	0.454	41.314	0.002
	ALB	-0.001	0.622	25.842	0.135
	AFS	-0.001	0.534	40.063	0.001

ANM, age at menopause; AAM, age at menarche; LMC, length of menstrual cycle; AFB, age at first birth; ALB, Age at last live birth; AFS, Age first had sexual intercourse; BMI, Body mass index; LBP, low back pain.

### MR analysis of each feature related to hormonal and reproductive factors on LBP (validation analysis)

**Figure 5:**
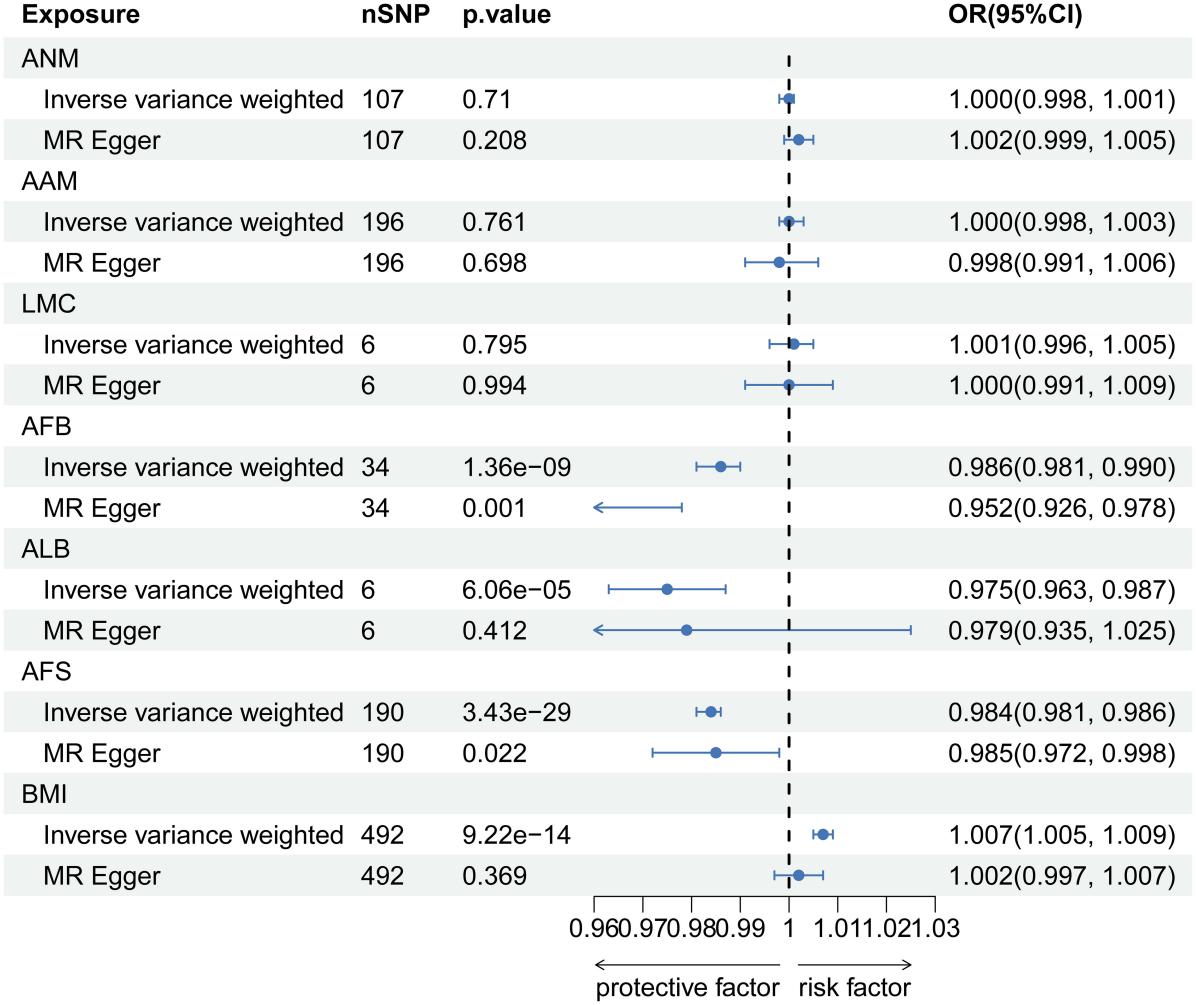
The causal relationship of genetically predicted Hormonal and Reproductive Factors and LBP (replication analysis).

After implementing the Bonferroni correction, the results from MR indicated a noteworthy association between a decreased risk of LBP and AAM, AFB, ALB and AFS. Nevertheless, no significant association was observed between ANM and LMC with LBP (**Figure 5**). Scatter plots, funnel plots and leave-one-out plots illustrating the association between reproductive and hormonal factors and LBP were presented in **Supplementary Figures S7**-**S9**. Heterogeneity and pleiotropy are depicted in **Table 4**.

**Table 4:** Sensitivity analysis of hormonal and reproductive factors causally linked to LBP (validation analysis).

Exposure	Outcome	Pleiotropy		Heterogeneity	
		Horizontal pleiotropy (Egger intercept)	Horizontal pleiotropy (*p*-value)	Heterogeneity (*Q*)	Heterogeneity (*p*-value)
ANM	LBP	-1.18E-04	0.097	112.910	0.305
AAM		4.10E-05	0.594	256.622	0.002
LMC		3.97E-05	0.899	3.281	0.657
AFB		0.001	0.015	26.194	0.794
ALB		-9.84E-05	0.873	6.580	0.254
AFS		-1.79E-05	0.873	201.169	0.259

ANM, age at menopause; AAM, age at menarche; LMC, length of menstrual cycle; AFB, age at first birth; ALB, Age at last live birth; AFS, Age first had sexual intercourse; BMI, Body mass index; LBP, low back pain.

## Discussion

Our study utilized a two-sample MR analysis to evaluate the potential causal effects of six hormonal and reproductive factors on the development of LBP. We uncovered novel insights regarding the influence of AAM, AFB, ALB and AFS on LBP. Through Bonferroni correction, we identified a negative causal relationship between these factors and the aforementioned spinal conditions. Specifically, early menarche, early age at first birth, early age at last live birth and early age first had sexual intercourse may elevate the risk of LBP. The verification results were consistent with the initial findings. After controlling for BMI, the association between AFB and LBP persisted, while the correlation between AAM, ALB, AFS and LBP did not endure. These insights underscore the importance of investigating hormonal and reproductive factors in spinal health, providing valuable directions for future research and clinical applications. We also recommend enhanced monitoring of women with these characteristics to proactively manage LBP.

Numerous observational studies have substantiated the connection between hormonal factors, reproductive factors and LBP. Nevertheless, there remains uncertainty regarding the potential influence of ANM, AAM, LMC, AFB, AFS and ALB on the development of LBP. The outcomes of the longitudinal cohort investigation aligned with our findings, affirming that an earlier AAM onset was associated with an increased likelihood of experiencing LBP (19). Other studies have also noted a positive association, with a cross-sectional study of more than 298,000 women discovering a positive link between early menarche and LBP (p<0.001) (32). Onset of menarche at age less than 11 years has been linked to a higher risk of experiencing LBP, as indicated by findings from both cross-sectional and cohort studies (18). However, it has also been shown that no association was found between ANM or AAM and risk of LBP (33). The existence of these contradictions could be attributed to potential bias in traditional epidemiological methods caused by confounding variables. Thus, employing MR methods could elucidate causality at the genetic level.

Many studies have shown that the prevalence of LBP in women was not significantly correlated with age, and the prevalence of LBP in the postmenopausal period was significantly different from that in the premenopausal period (34, 35). However, the Mexican study revealed that women with back pain were more likely to be older (36). Adera et al. conducted a population-based cross-sectional study that elucidates a noteworthy correlation between premature menopause and an escalated susceptibility to LBP (37). Our findings at the genetic level provide evidence that ANM was not causally associated with LBP, corroborating prior studies. Instead, the occurrence of LBP and IVDD in menopausal women might be related to a rapid decrease in androgen levels. Scholarly investigations have predominantly utilized menarche as a parameter in delineating pubertal onset. However, pubertal development was a complex process that entails a spectrum of changes across various bodily systems (38). Furthermore, researchers concur that the commencement of menarche may not be the optimal indicator, as a substantial portion of growth and the emergence of secondary sexual characteristics precede its occurrence (39, 40). Prolonged and heightened exposure to estrogen over an extended period was postulated as an additional contributory factor to the increased susceptibility to LBP among women displaying early onset of menarche (18, 41).

A cross-sectional study showed that younger maternal age at the time of first birth (especially <20 years) was associated with chronic LBP, which was similar to our results (21). Meanwhile, in a prospective study, a statistically significant distinction was noted in the prevalence of LBP during pregnancy between younger and older women (42). Meanwhile, Heuch et al. have reported an association between the incidence of lumbar discomfort and advancing age, as well as the cumulative instances of pregnancies (43). Our investigation revealed an observation wherein a heightened susceptibility to dorsal discomfort was discerned among youthful females. The MR method employed mitigates biases arising from various factors, including confounding, through genetic allelic assignment principles. This method corroborates, at the genetic level, the notion that an early AFB constitutes a risk factor for LBP. This phenomenon could stem from elevated hormone levels that impact the soft tissues supporting the spine, potentially leading to enduring laxity in joints and ligaments (43–46). This correlation aligns with an elevated risk of LBP observed in women undergoing hormone replacement therapy or using oral contraceptives (17, 20). Additionally, younger women demonstrate heightened sensitivity to hormonal variations in estrogen and relaxin, leading to more pronounced collagen relaxation (47, 48). This sensitivity may elucidate the increased risk of LBP among women giving birth at a younger age. Moreover, compression of the uterus on the developing spine during the first childbirth in younger girls may contribute to the onset of low back pain (43).

The prospective study by Brynhildsen et al. found that hormonal fluctuations during the menstrual cycle do not influence LBP (21). In contrast, Wijnhoven et al. identified a link between chronic LBP and irregular or prolonged menstrual cycles (20). Our study aligns with Brynhildsen et al.’s conclusion that shorter menstrual cycles are not associated with an increased risk of lower back pain LBP. This suggests that the menstrual cycle length is not a risk factor for LBP.

Several studies have documented the increasing severity of IVDD in women as they age (49–51), with a notably more rapid degeneration observed in females after the age of 60 compared to males (52). Epidemiological evidence supports the notion that disc degeneration correlates with age (53). This phenomenon was similarly observed by De Schepper et al. (54). The role of estrogen in IVD metabolism and its expression in annulus fibrosus and nucleus pulposus cells may explain these observations (55). IVD is the primary cause of LBP, with hormone levels playing a crucial role. Further investigation is needed to understand the specific mechanism of action.

Our study possesses several strengths. It marks the inaugural application of MR to investigate the causal relationship between hormonal and reproductive factors and LBP. Encompassing six distinct reproductive characteristics, our study offers a comprehensive understanding of the reproductive period. Utilizing data from a diverse range of cohorts enhances the reliability of our findings and minimizes overlap. Employing the principle of random allele assignment, we conducted a Bidirectional MR study to validate the robustness of these results. Furthermore, we corroborated the reliability of our conclusions through MVMR, with adjustments made for BMI.

However, the exclusive reliance on European GWAS data may limit the generalizability of our findings to other ethnic or geographic populations. Besides, the inclusion of both genders in the outcome data might also weaken the observed associations. The inclusion of both genders in the dataset introduced gender heterogeneity and potential bias. Ideally, the association between SNPs and outcome estimates should display gender heterogeneity. However, in the LBP GWAS database we used, with women comprising over 60%, it represents a predominantly female-led GWAS, thereby minimizing the likelihood of bias. Future MR studies should consider validating these results within female-only samples by appropriate stratification.

## Conclusions

In conclusion, our study explored the causal relationship between ANM, AAM, LMC, AFB, AFS, ALB and the prevalence of LBP. We found that early menarche, early age at first birth, early age at last live birth and early age first had sexual intercourse may decrease the risk of LBP. These insights enhance our understanding of LBP risk factors, offering valuable guidance for screening, prevention, and treatment strategies for at-risk women.

## Data availability statement

The original contributions presented in the study are included in the article/**Supplementary Material**. Further inquiries can be directed to the corresponding authors.

## Ethics statement

Ethical approval was unnecessary due to the public nature of the GWAS data.

## Author contributions

DC: Conceptualization, Funding acquisition, Investigation, Supervision, Visualization, Writing – original draft, Writing – review & editing. JZ: Conceptualization, Investigation, Methodology, Visualization, Writing – original draft, Writing – review & editing. JHL: Visualization, Writing – review & editing, Data curation. CL: Data curation, Visualization, Writing – review & editing. ZGZ: Formal analysis, Writing – review & editing. XR: Software, Writing – review & editing. JW: Formal analysis, Writing – review & editing. JFL: Formal analysis, Writing – review & editing. HC: Investigation, Writing – review & editing. FW: Investigation, Writing – review & editing. XL: Investigation, Writing – review & editing. MG: Methodology, Writing – review & editing. ZYZ: Conceptualization, Supervision, Writing – review & editing. YX: Conceptualization, Supervision, Writing – review & editing. SL: Conceptualization, Investigation, Supervision, Writing – original draft, Writing – review & editing.

## Supplementary material

Supplementary Figure S1 Scatter plot for LBP.

Supplementary Figure S2 Funnel plots for LBP.

Supplementary Figure S3 MR leave-one-out sensitivity analysis.

Supplementary Figure S4 Funnel plots for Hormonal and Reproductive Factors.

Supplementary Figure S5 reversed MR leave-one-out sensitivity analysis.

Supplementary Figure S6 Scatter plot for Hormonal and Reproductive Factors.

Supplementary Figure S7 Scatter plot for LBP (replication analysis).

Supplementary Figure S8 Funnel plots for LBP (replication analysis).

Supplementary Figure S9 MR leave-one-out sensitivity analysis (replication analysis).

Supplementary Table S1 SNPs of ANM for LBP in MR.

Supplementary Table S2 SNPs of AAM for LBP in MR.

Supplementary Table S3 SNPs of LMC for LBP in MR.

Supplementary Table S4 SNPs of AFB for LBP in MR.

Supplementary Table S5 SNPs of ALB for LBP in MR.

Supplementary Table S6 SNPs of AFS for LBP in MR.

Supplementary Table S7 SNPs of BMI for LBP in MR.

Supplementary Table S8 The SNPs selected for BMI factors in MVMR analysis.

Supplementary Table S9 SNPs of LBP for Hormonal and Reproductive Factors in MR.
