# Supplementary material for: A causal examination of the correlation between hormonal and reproductive factors and low back pain
Source: Front Endocrinol (Lausanne). 2024 May 10;15:1326761. doi: 10.3389/fendo.2024.1326761 (PMC11116661; doi:10.3389/fendo.2024.1326761)
Supplement: Supplementary Figure S1 — Scatter plot for LBP. [file DataSheet_1.pdf]

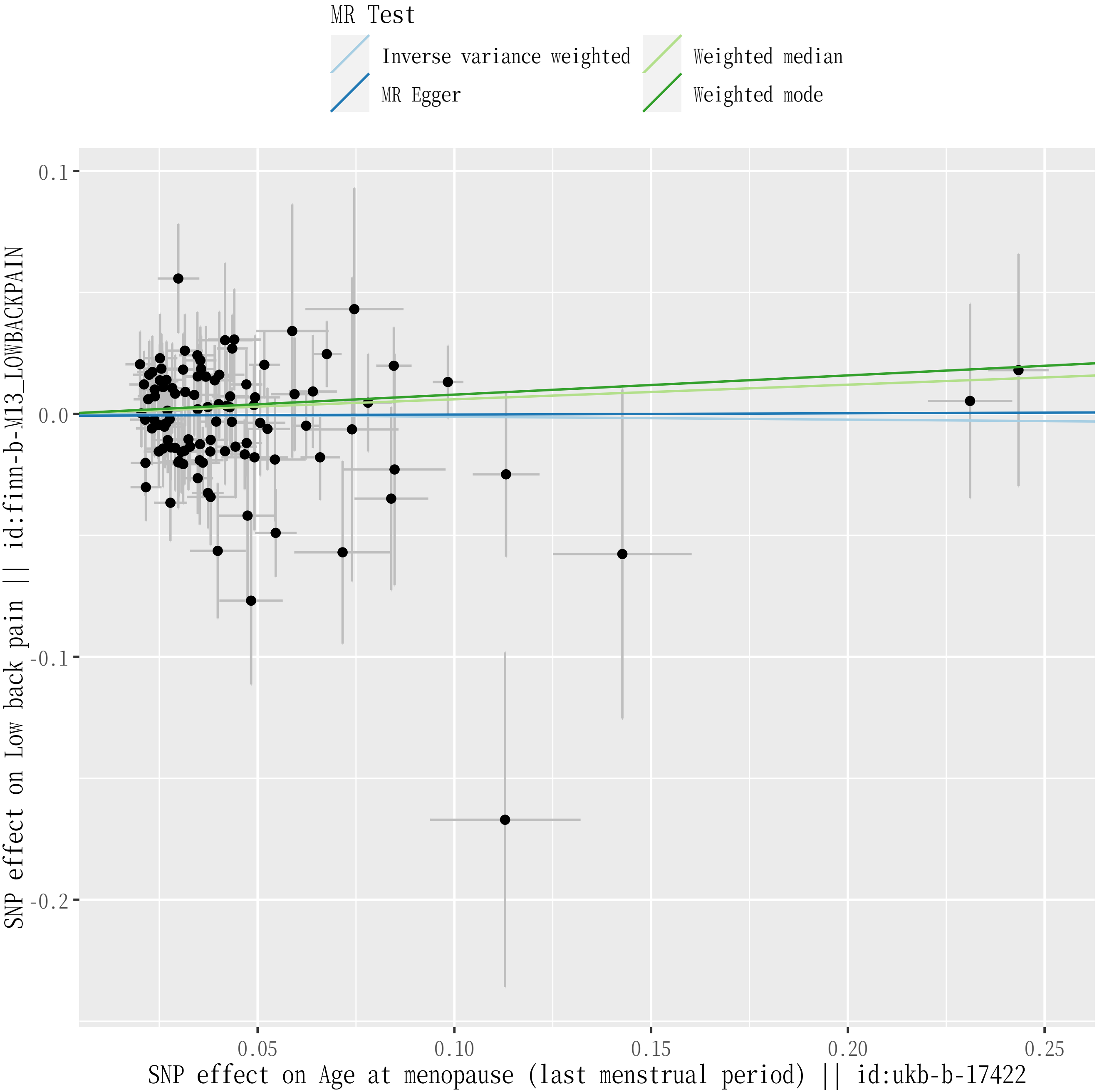

MR Test
Inverse variance weighted
MR Egger
Weighted median
Weighted mode
0.1
0.0
-0.1
-0.2
SNP effect on Low back pain || id:finn-b-M13_LOWBACKPAIN
0.05
0.10
0.15
0.20
0.25
SNP effect on Age at menopause (last menstrual period) || id:ukb-b-17422

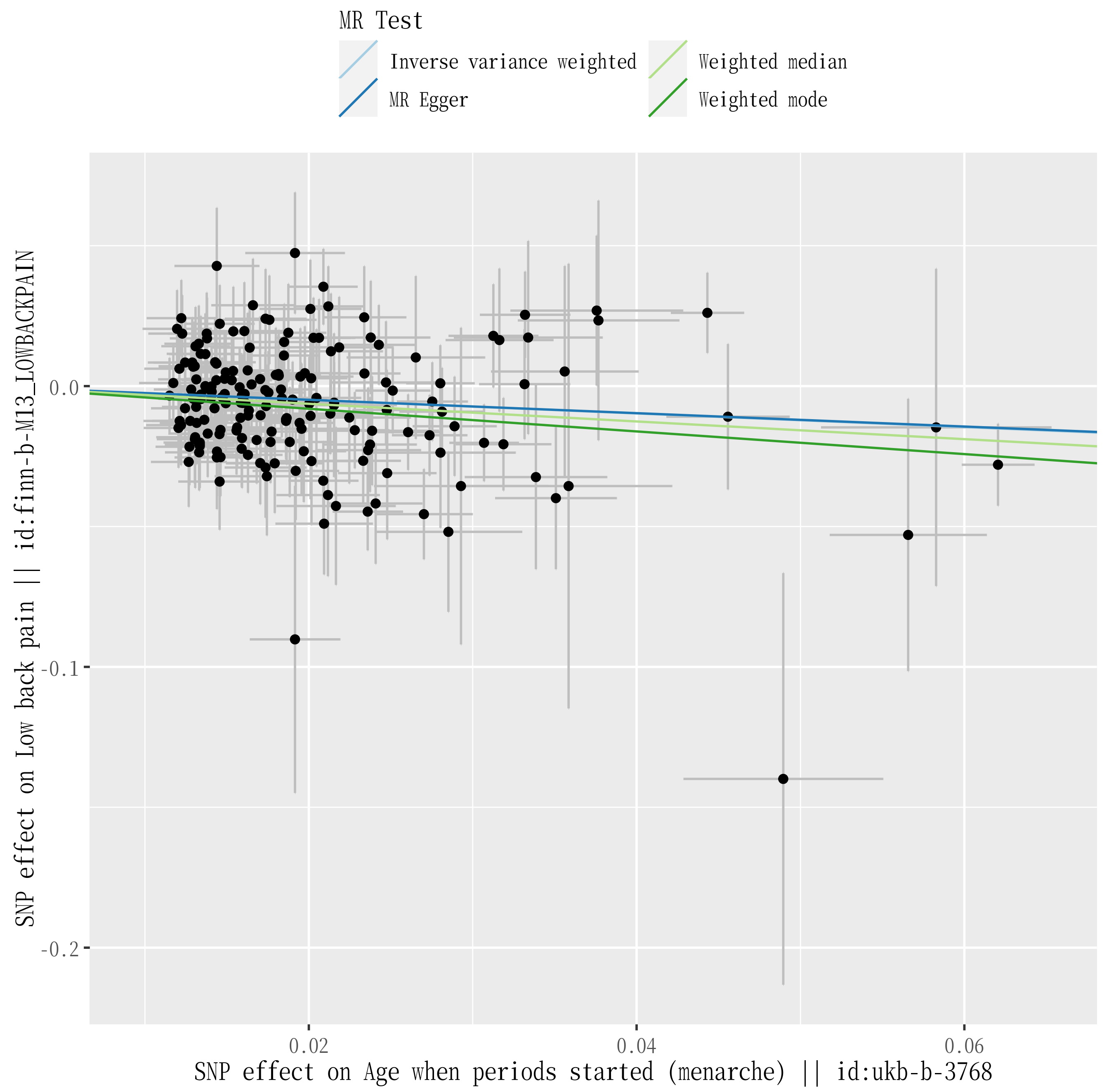

MR Test
Inverse variance weighted
MR Egger
Weighted median
Weighted mode
0.0
-0.1
-0.2
0.02
0.04
0.06
SNP effect on Low back pain || id:finn-b-M13_LOWBACKPAIN
SNP effect on Age when periods started (menarche) || id:ukb-b-3768

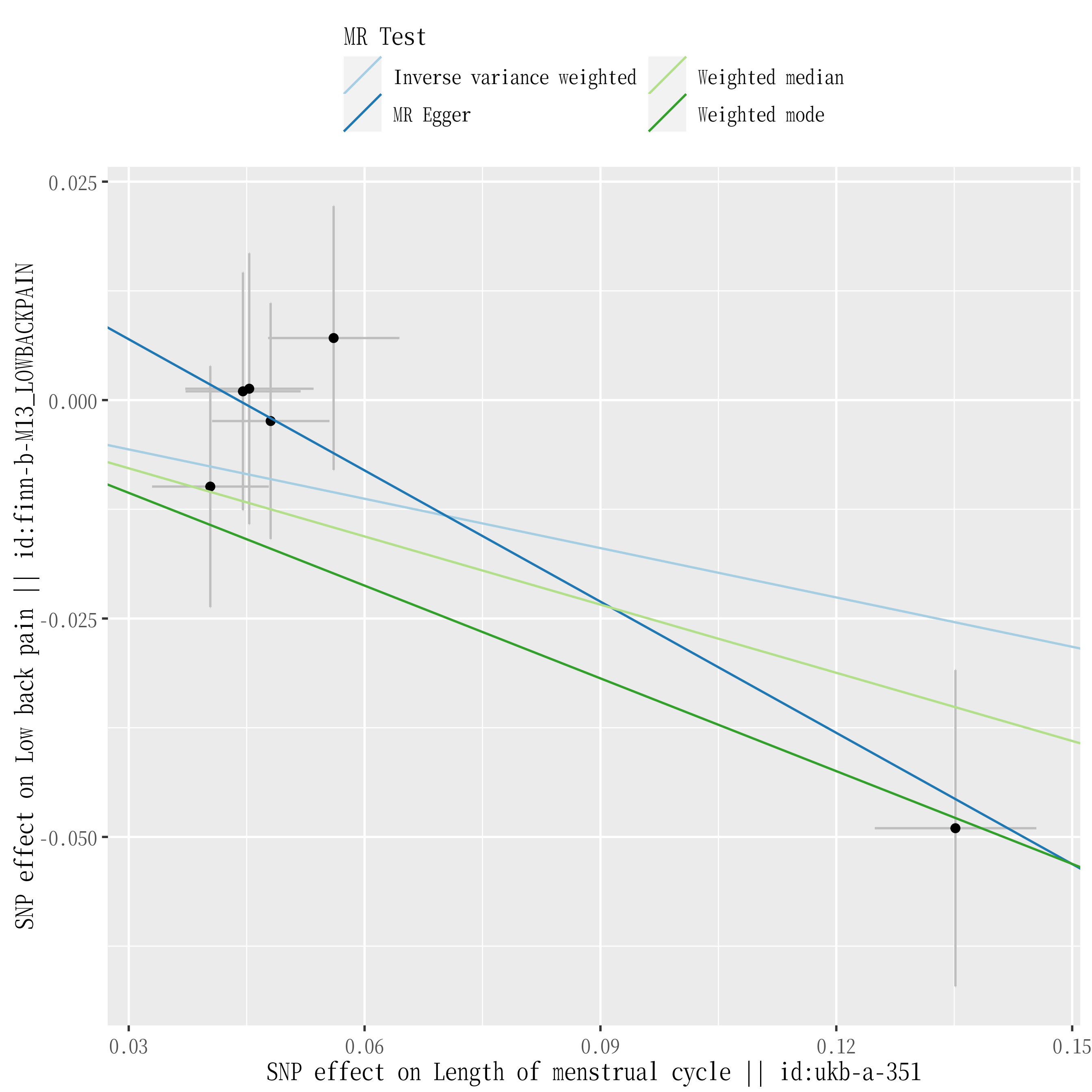

MR Test
Inverse variance weighted
Weighted median
MR Egger
Weighted mode
0.025
0.000
-0.025
-0.050
0.03
0.06
0.09
0.12
0.15
SNP effect on Low back pain || id:finn-b-M13_LOWBACKPAIN
SNP effect on Length of menstrual cycle || id:ukb-a-351

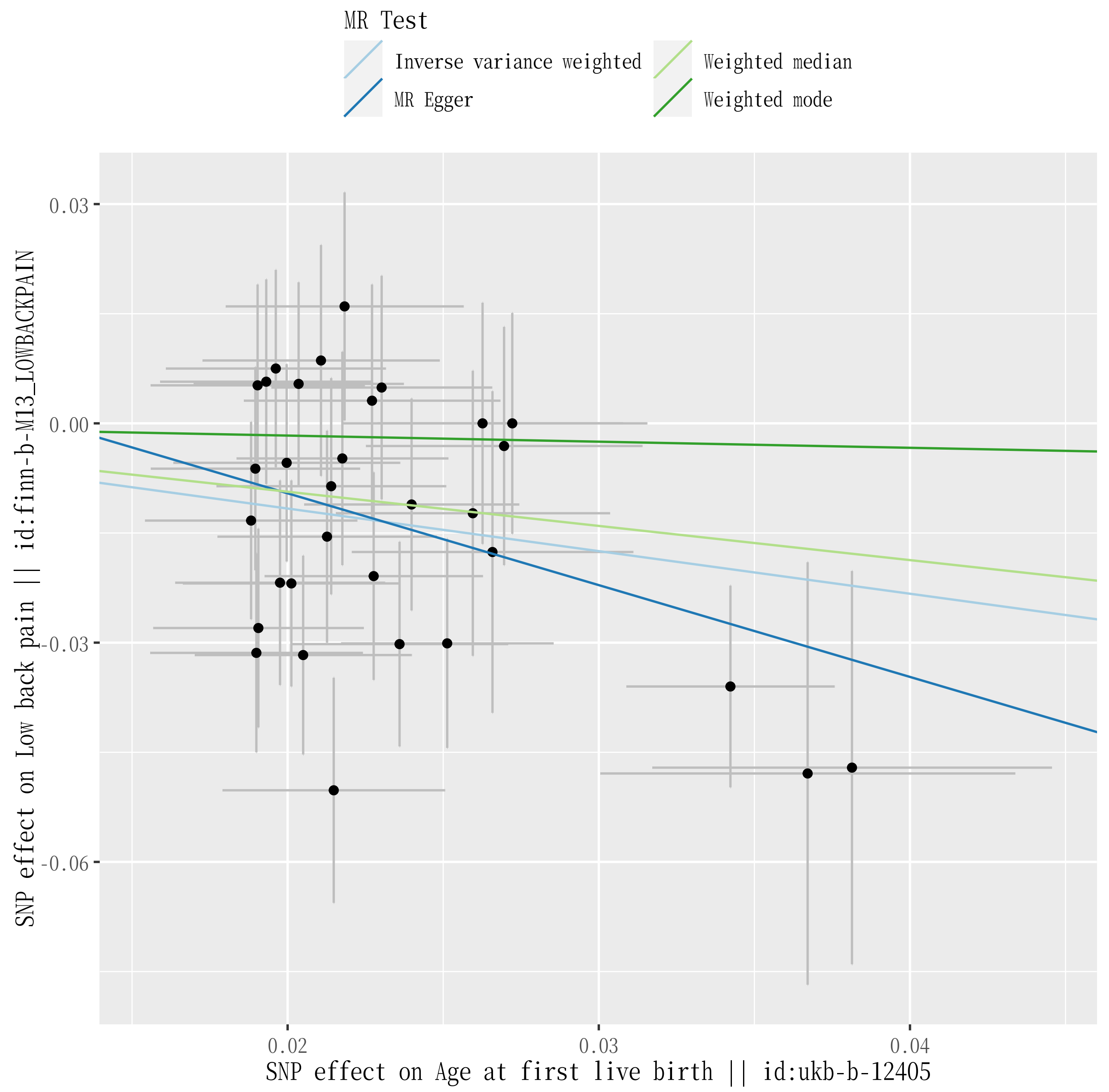

MR Test
Inverse variance weighted
MR Egger
Weighted median
Weighted mode
0.03
0.00
-0.03
-0.06
SNP effect on Low back pain || id:finn-b-M13_LOWBACKPAIN
0.02
0.03
0.04
SNP effect on Age at first live birth || id:ukb-b-12405

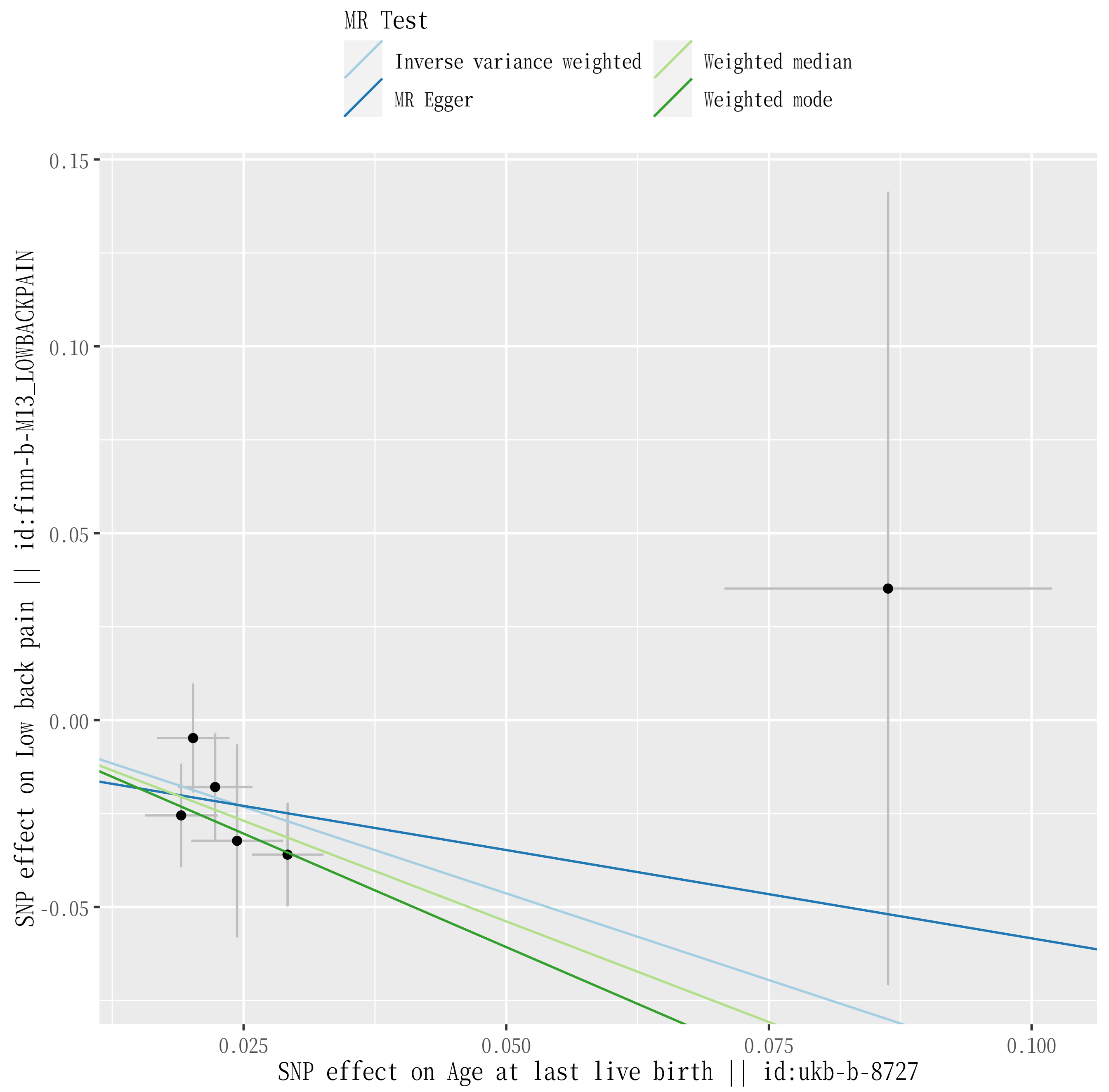
MR Test
Inverse variance weighted
MR Egger
Weighted median
Weighted mode
0.15
0.10
0.05
0.00
-0.05
0.025
0.050
0.075
0.100
SNP effect on Low back pain || id:finn-b-M13_LOWBACKPAIN
SNP effect on Age at last live birth || id:ukb-b-8727

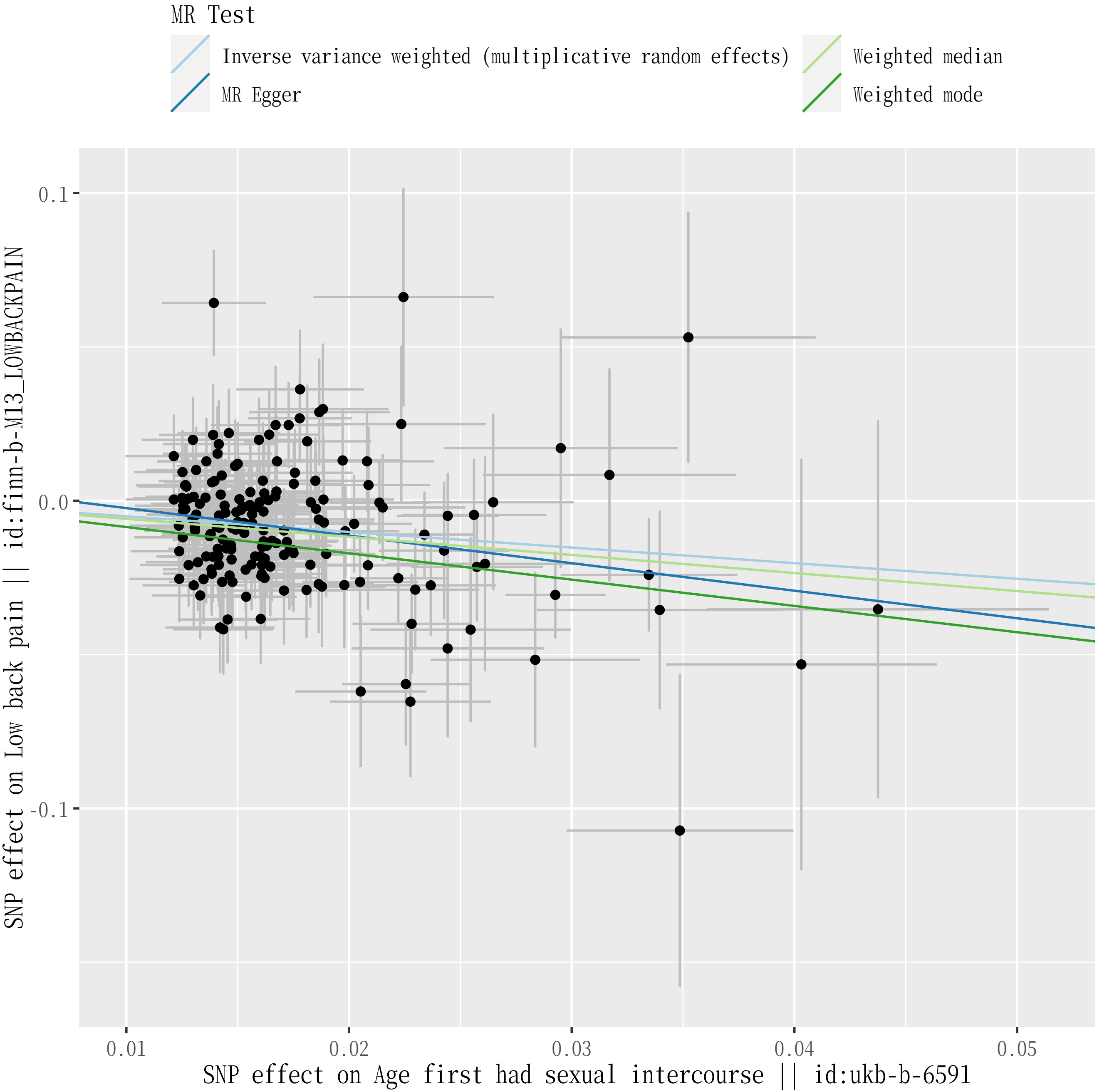
MR Test
Inverse variance weighted (multiplicative random effects)
MR Egger
Weighted median
Weighted mode
0.1
0.0
-0.1
0.01
0.02
0.03
0.04
0.05
SNP effect on Low back pain || id:finn-b-M13_LOWBACKPAIN
SNP effect on Age first had sexual intercourse || id:ukb-b-6591

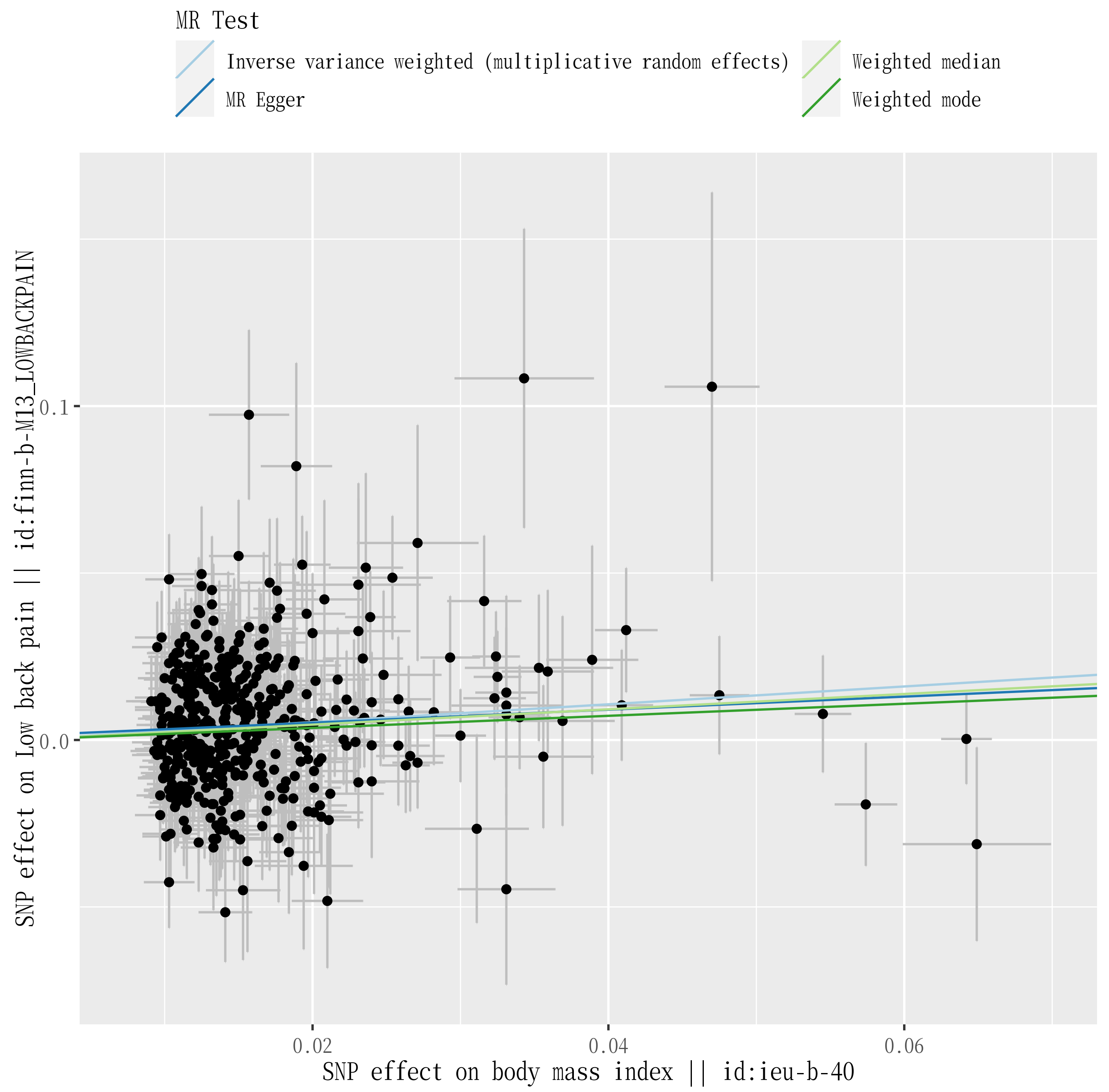
MR Test
Inverse variance weighted (multiplicative random effects)
MR Egger
Weighted median
Weighted mode
SNP effect on Low back pain || id:finn-b-M13_LOWBACKPAIN
0.1
0.0
0.02
0.04
0.06
SNP effect on body mass index || id:ieu-b-40
