# Supplementary material for: A causal examination of the correlation between hormonal and reproductive factors and low back pain
Source: Front Endocrinol (Lausanne). 2024 May 10;15:1326761. doi: 10.3389/fendo.2024.1326761 (PMC11116661; doi:10.3389/fendo.2024.1326761)
Supplement: Supplementary Figure S2 — Funnel plots for LBP. [file DataSheet_2.pdf]

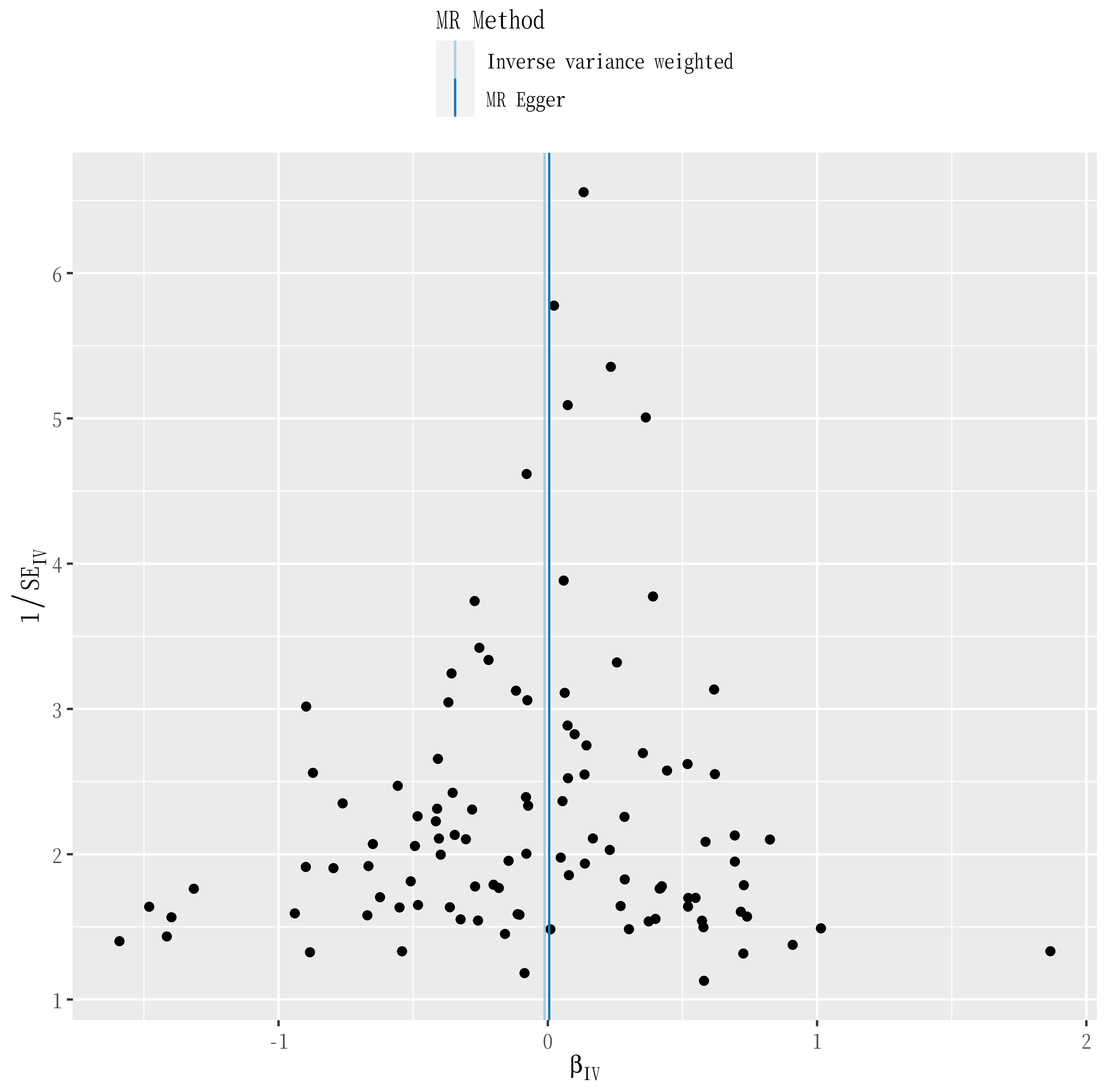

MR Method
Inverse variance weighted
MR Egger
6
5
4
3
2
1
$1/SE_{IV}$
-1
0
1
2
β_{IV}

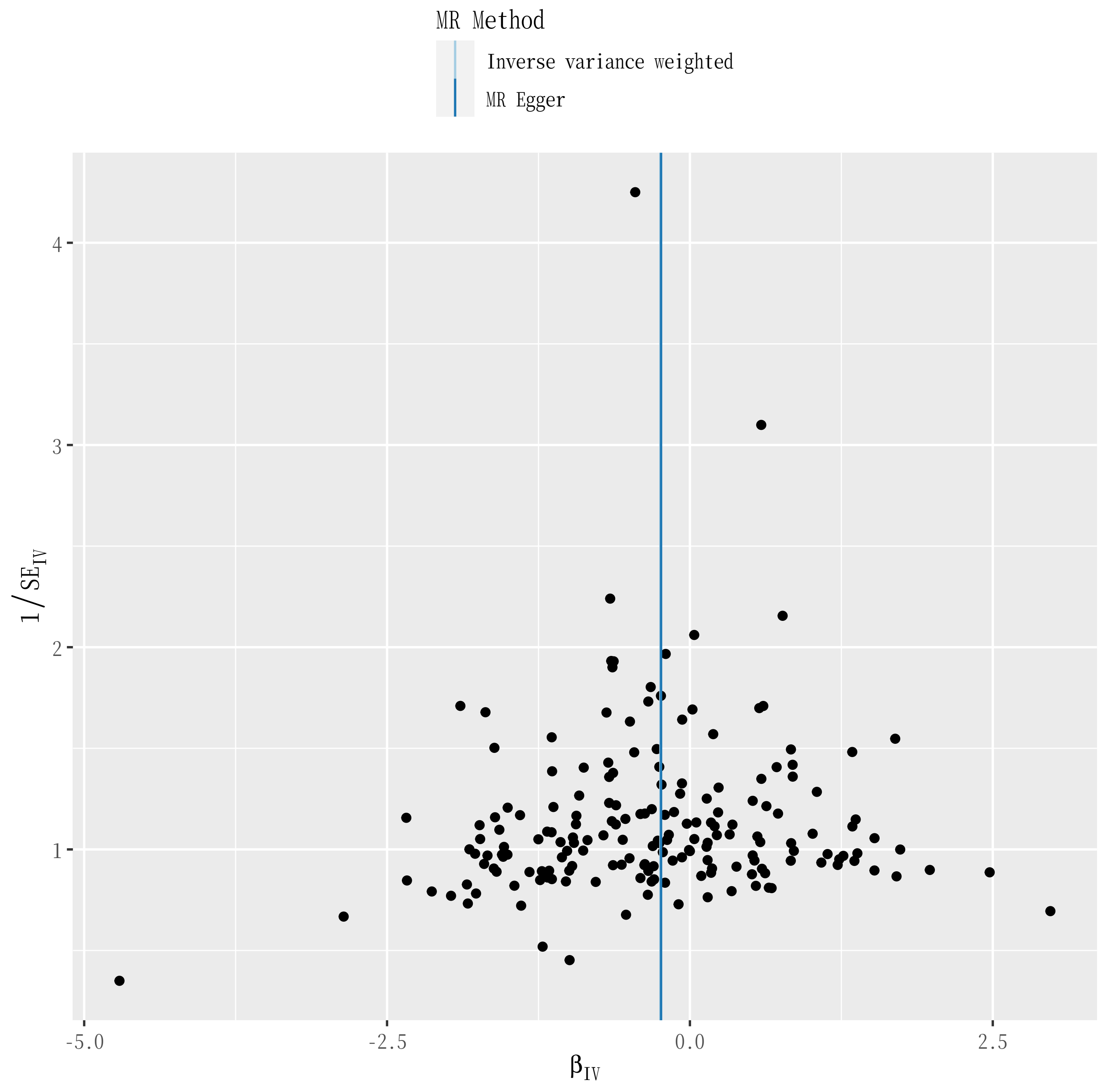
MR Method
Inverse variance weighted
MR Egger
4
3
2
1
$1/SE_{IV}$
-5.0
-2.5
0.0
2.5
β_{IV}

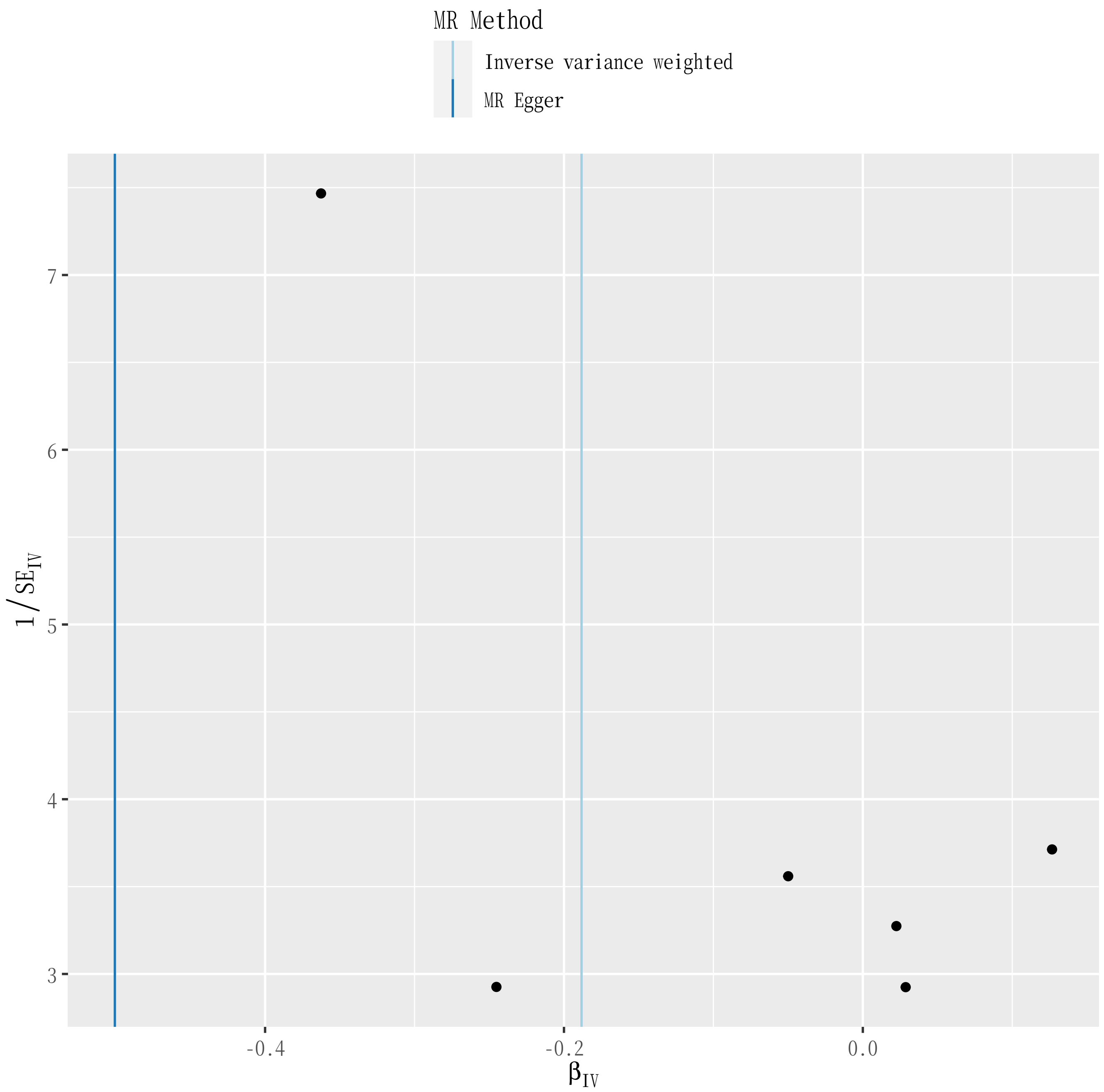
MR Method
Inverse variance weighted
MR Egger
$1/SE_{IV}$
7
6
5
4
3
-0.4
-0.2
0.0
β_{IV}

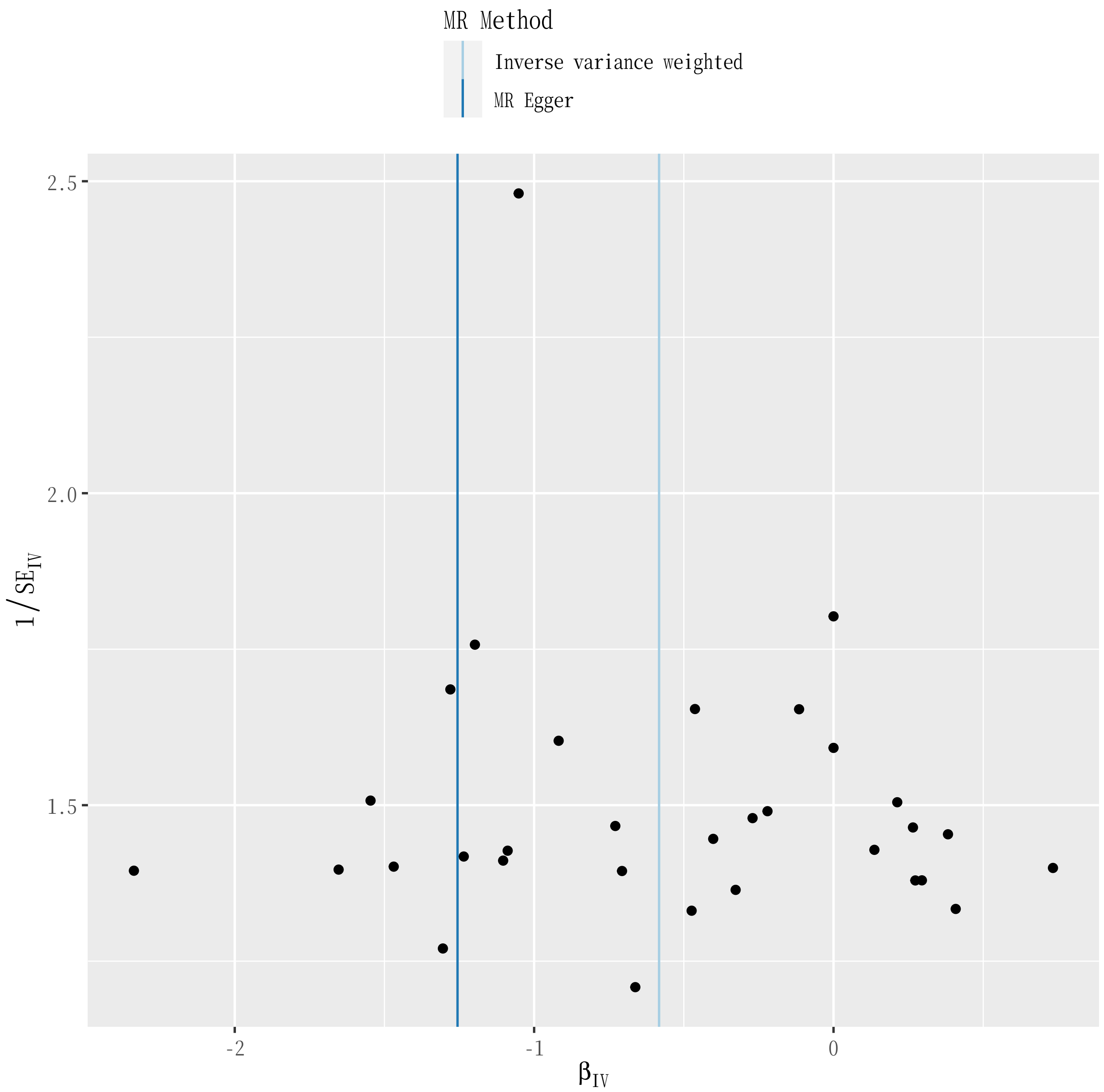

MR Method
Inverse variance weighted
MR Egger
2.5
2.0
1.5
1/SE_IV
-2
-1
0
β_IV

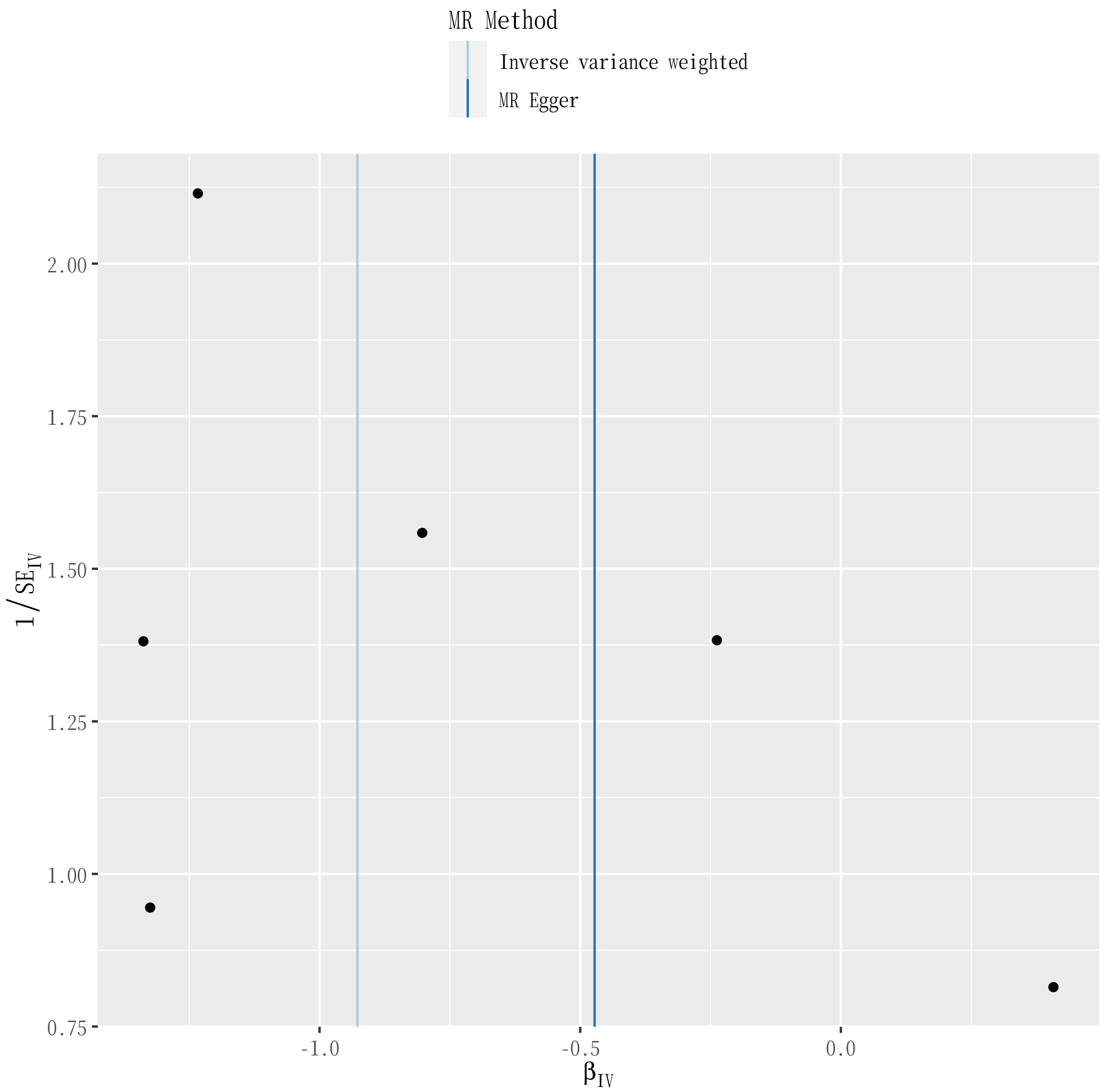
MR Method
Inverse variance weighted
MR Egger
2.00
1.75
1.50
1.25
1.00
0.75
1/SE_IV
-1.0
-0.5
0.0
β_IV

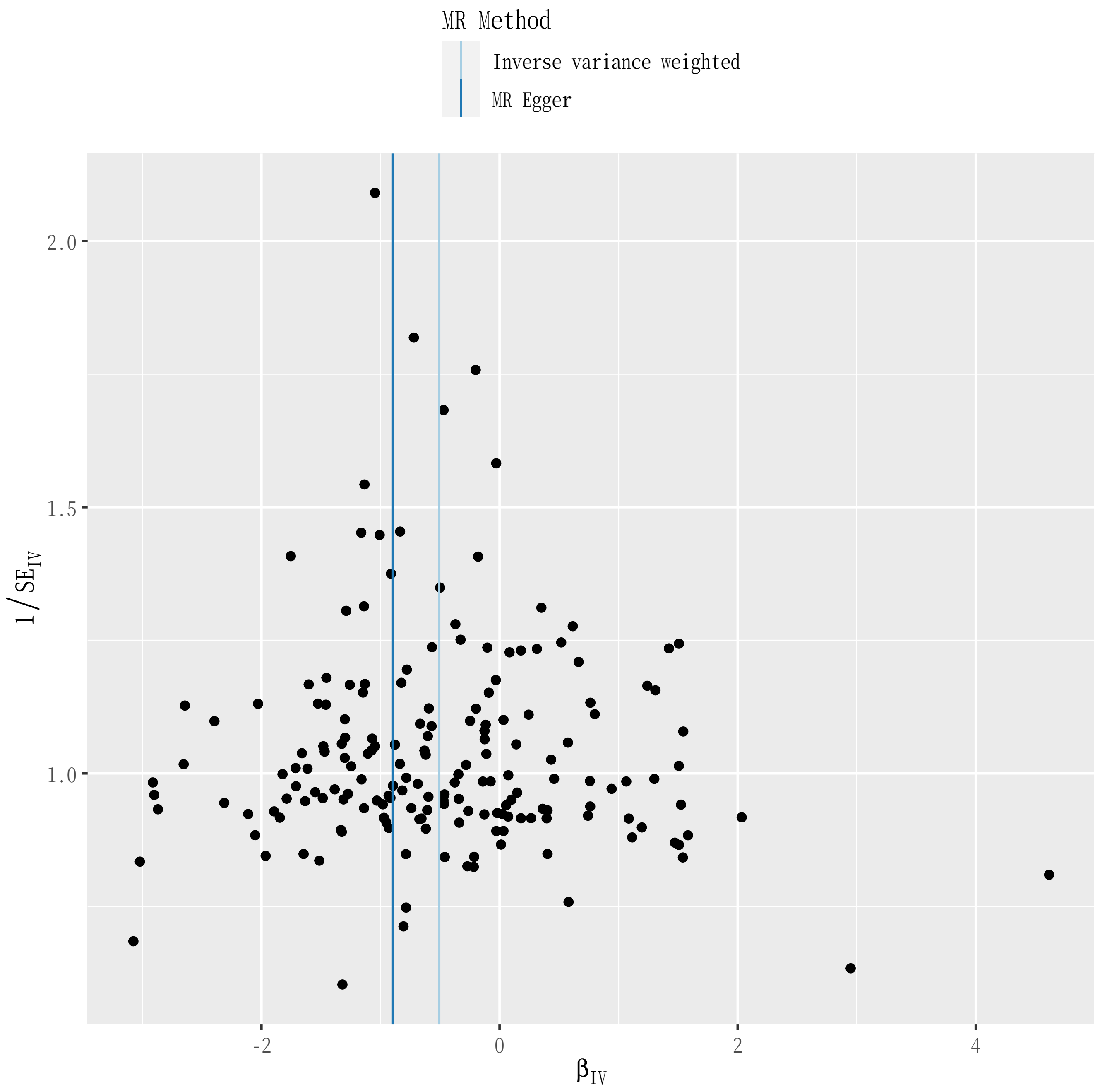

MR Method
Inverse variance weighted
MR Egger
2.0
1.5
1.0
$1/SE_{IV}$
-2
0
2
4
β_{IV}

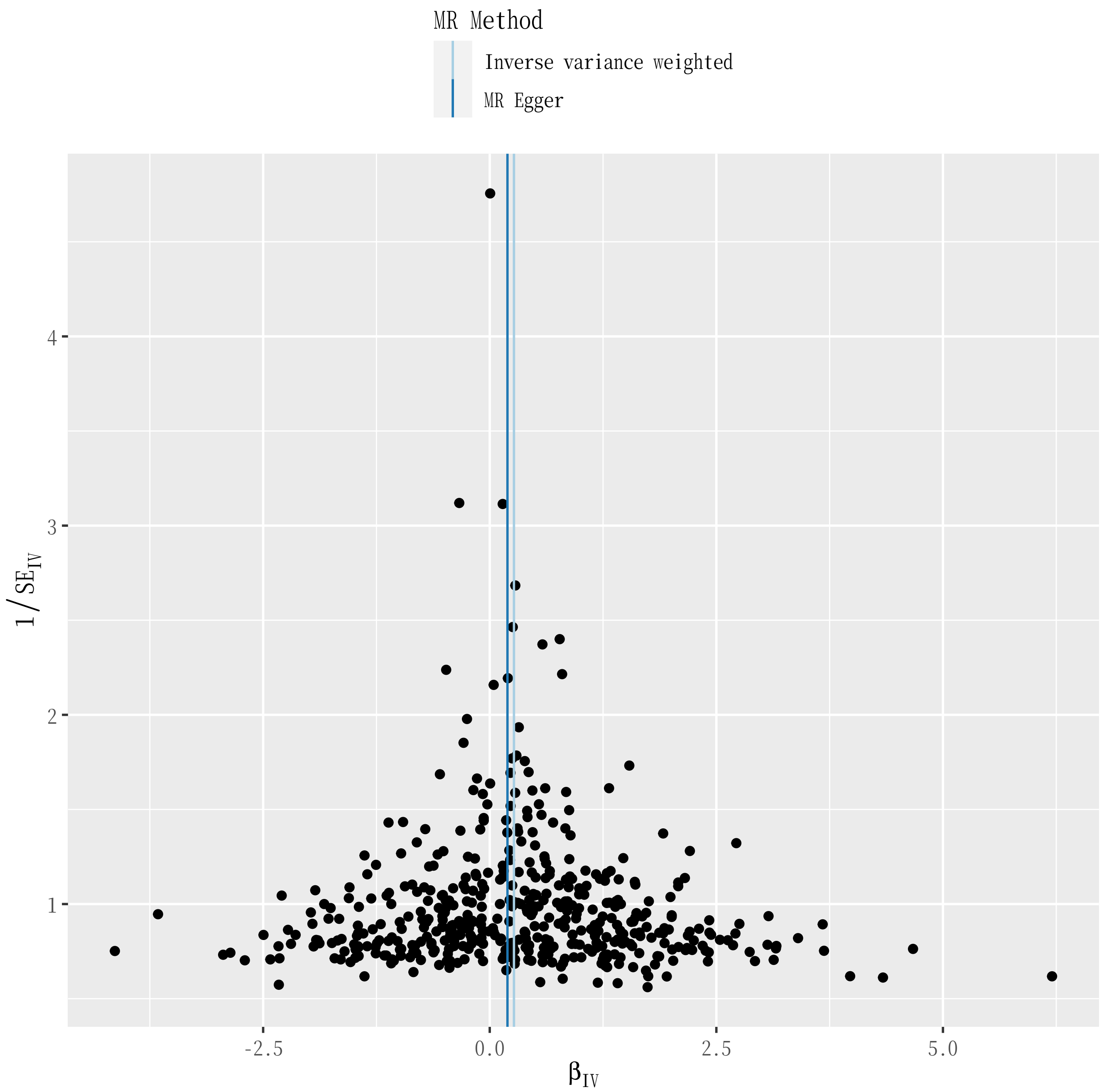
MR Method
Inverse variance weighted
MR Egger
1/SE_IV
4
3
2
1
-2.5
0.0
2.5
5.0
β_IV
