# Supplementary material for: A causal examination of the correlation between hormonal and reproductive factors and low back pain
Source: Front Endocrinol (Lausanne). 2024 May 10;15:1326761. doi: 10.3389/fendo.2024.1326761 (PMC11116661; doi:10.3389/fendo.2024.1326761)
Supplement: Supplementary Figure S3 — MR leave-one-out sensitivity analysis. [file DataSheet_3.pdf]

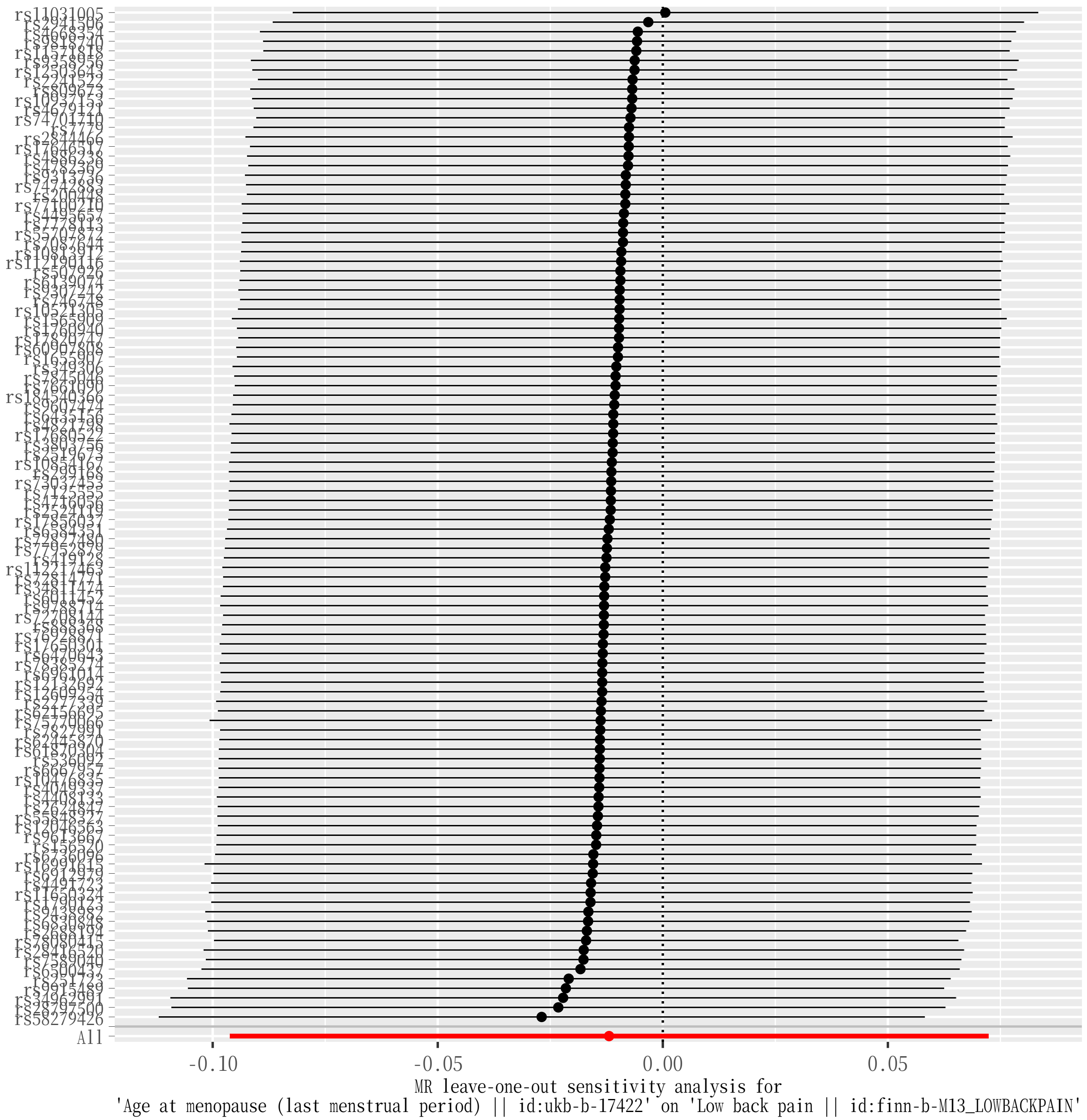

rs11031005
rs58279426
All
-0.10
-0.05
0.00
0.05
MR leave-one-out sensitivity analysis for
'Age at menopause (last menstrual period) || id:ukb-b-17422' on 'Low back pain || id:finn-b-M13_LOWBACKPAIN'

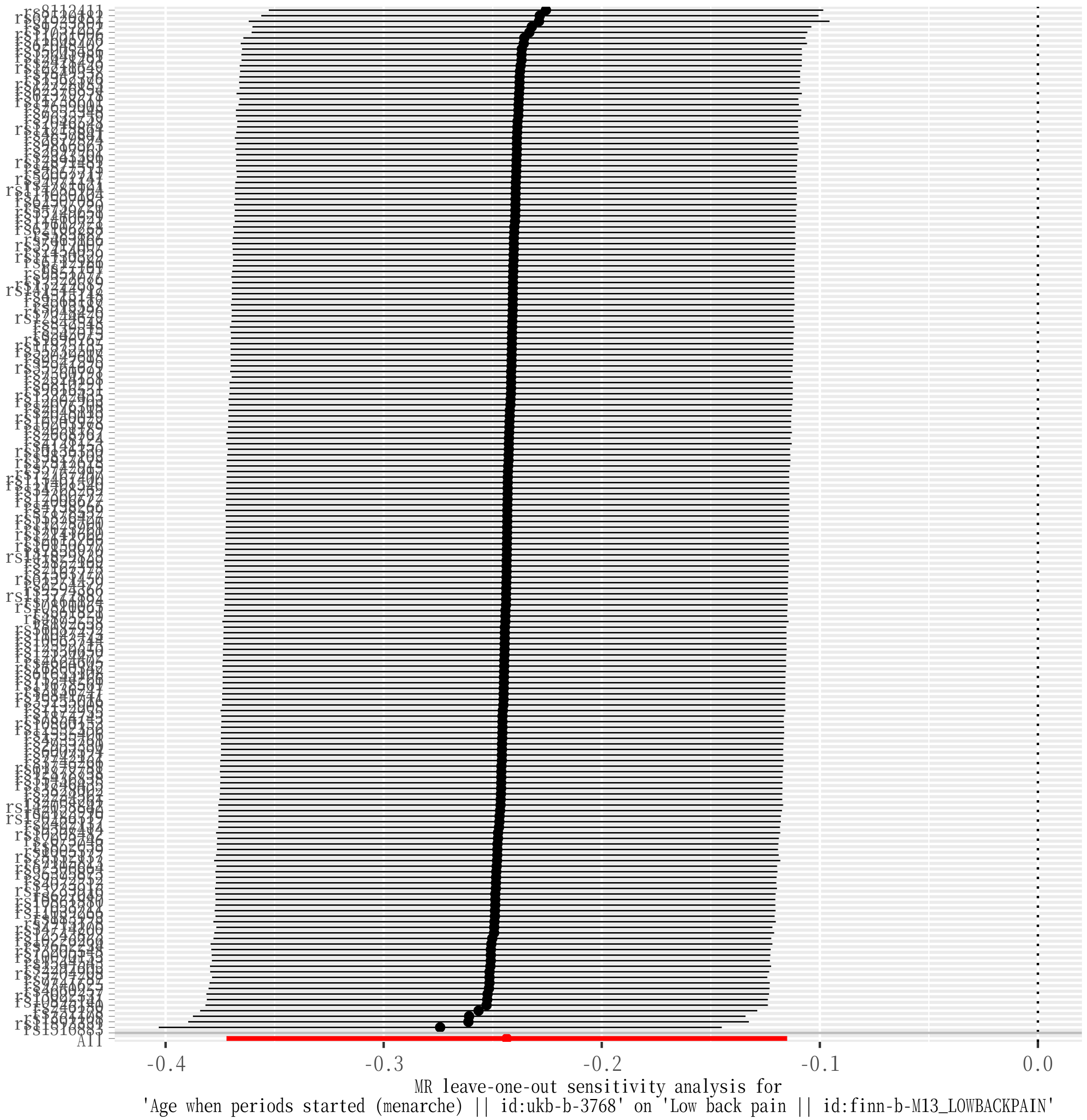
All
-0.4
-0.3
-0.2
-0.1
0.0
MR leave-one-out sensitivity analysis for
'Age when periods started (menarche) || id:ukb-b-3768' on 'Low back pain || id:finn-b-M13_LOWBACKPAIN'

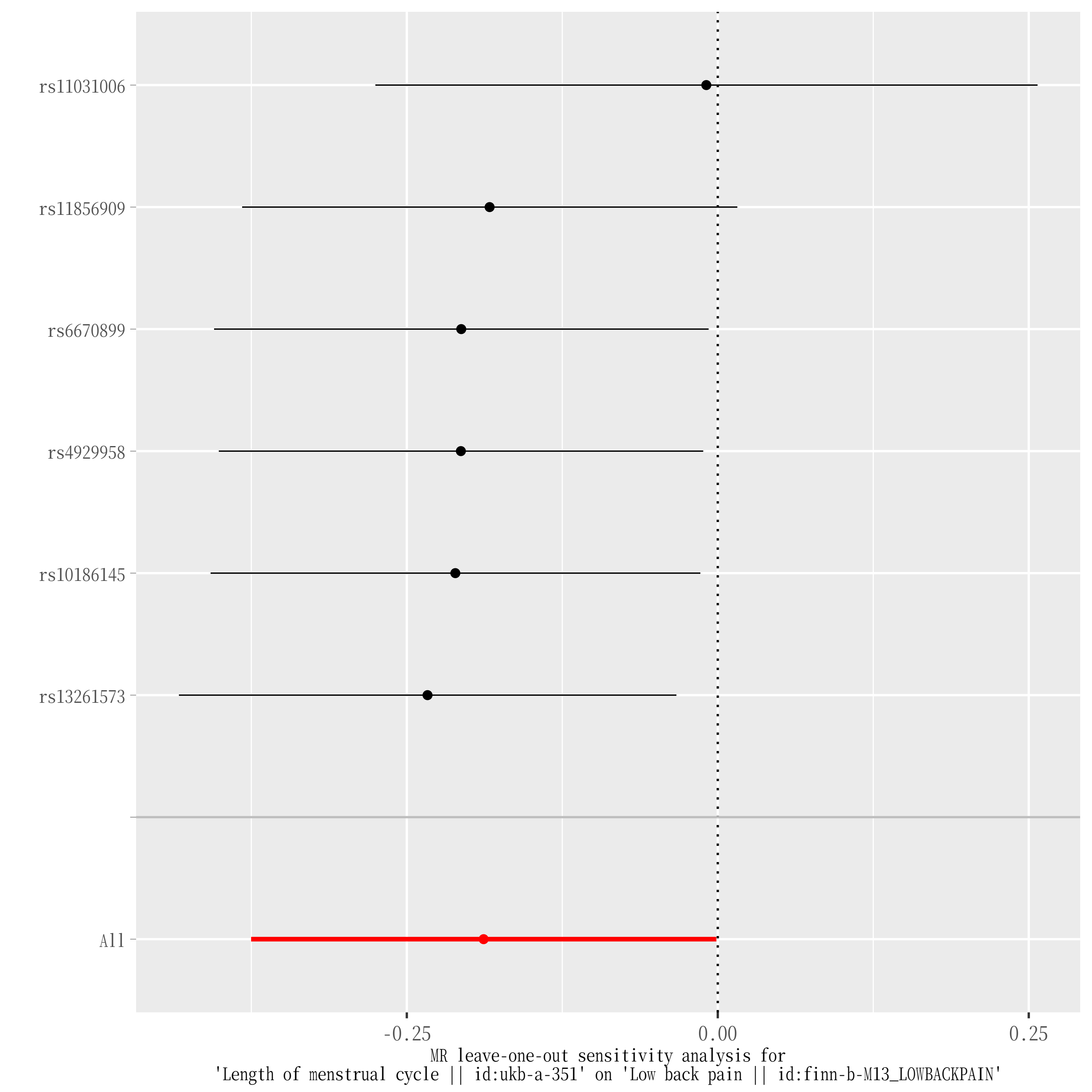
rs11031006
rs11856909
rs6670899
rs4929958
rs10186145
rs13261573
All
-0.25
0.00
0.25
MR leave-one-out sensitivity analysis for
'Length of menstrual cycle || id:ukb-a-351' on 'Low back pain || id:finn-b-M13_LOWBACKPAIN'

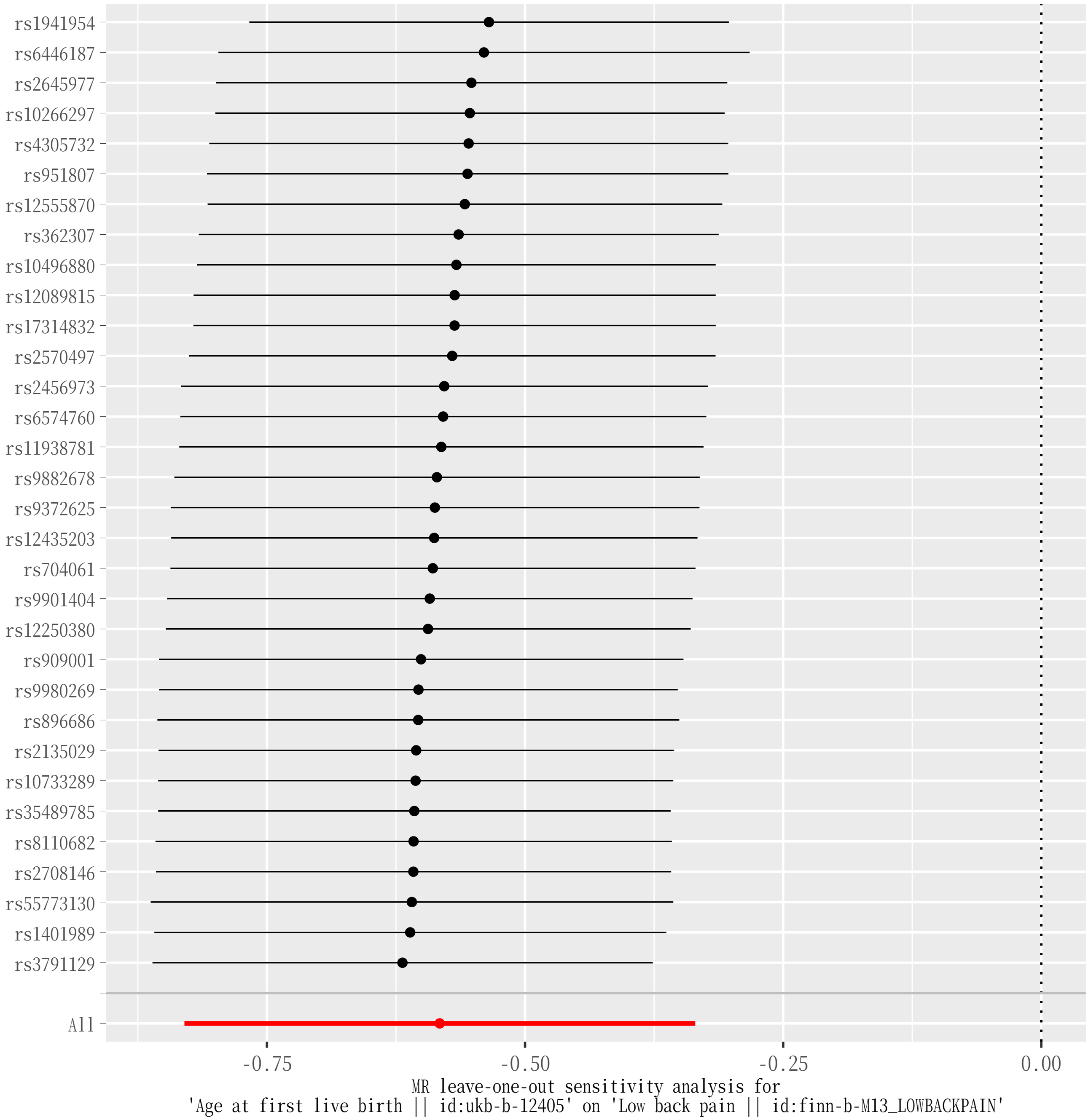
rs1941954
rs6446187
rs2645977
rs10266297
rs4305732
rs951807
rs12555870
rs362307
rs10496880
rs12089815
rs17314832
rs2570497
rs2456973
rs6574760
rs11938781
rs9882678
rs9372625
rs12435203
rs704061
rs9901404
rs12250380
rs909001
rs9980269
rs896686
rs2135029
rs10733289
rs35489785
rs8110682
rs2708146
rs55773130
rs1401989
rs3791129
All
-0.75
-0.50
-0.25
0.00
MR leave-one-out sensitivity analysis for
'Age at first live birth || id:ukb-b-12405' on 'Low back pain || id:finn-b-M13_LOWBACKPAIN'

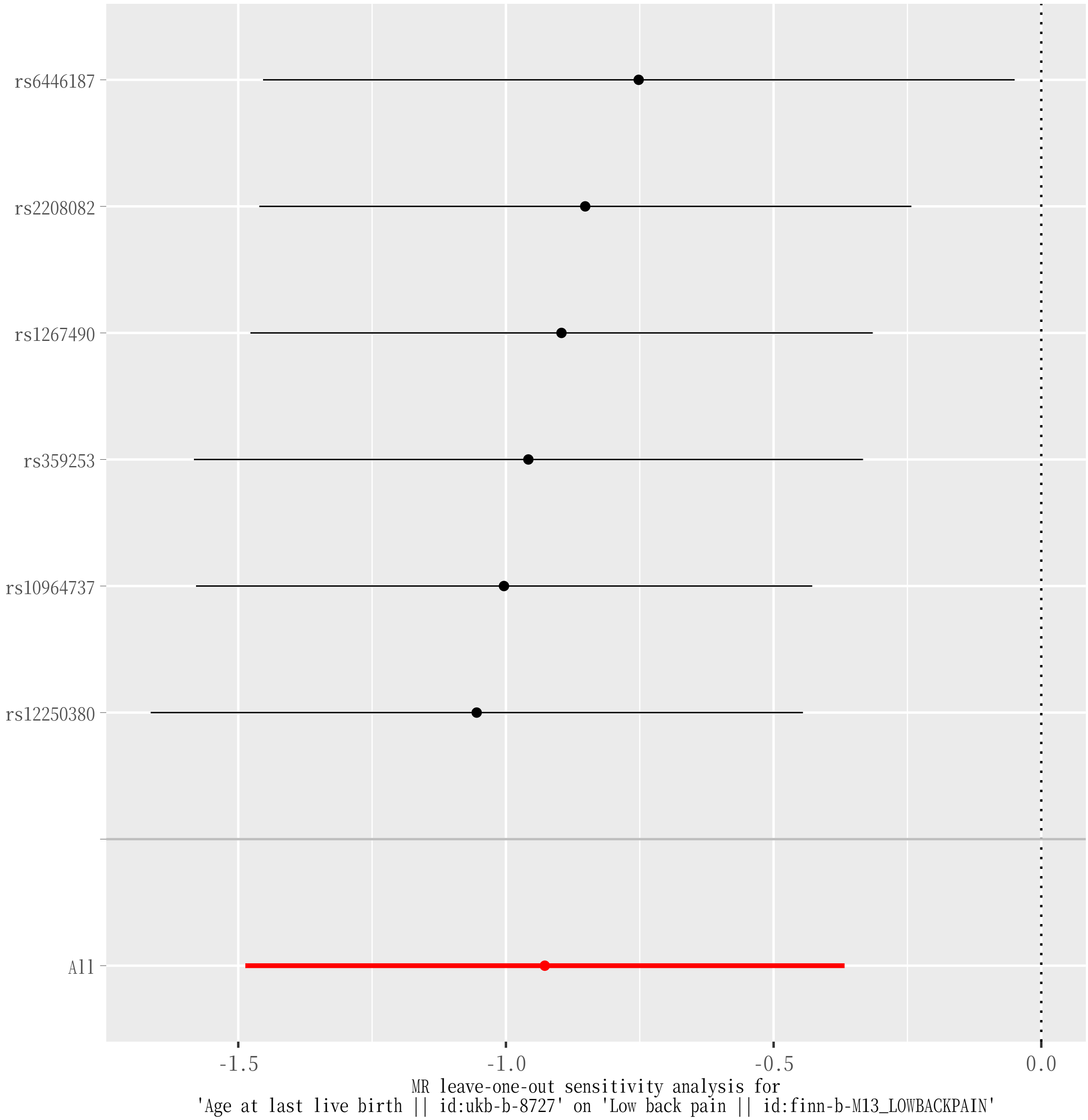

rs6446187
rs2208082
rs1267490
rs359253
rs10964737
rs12250380
All
-1.5
-1.0
-0.5
0.0
MR leave-one-out sensitivity analysis for
'Age at last live birth || id:ukb-b-8727' on 'Low back pain || id:finn-b-M13_LOWBACKPAIN'

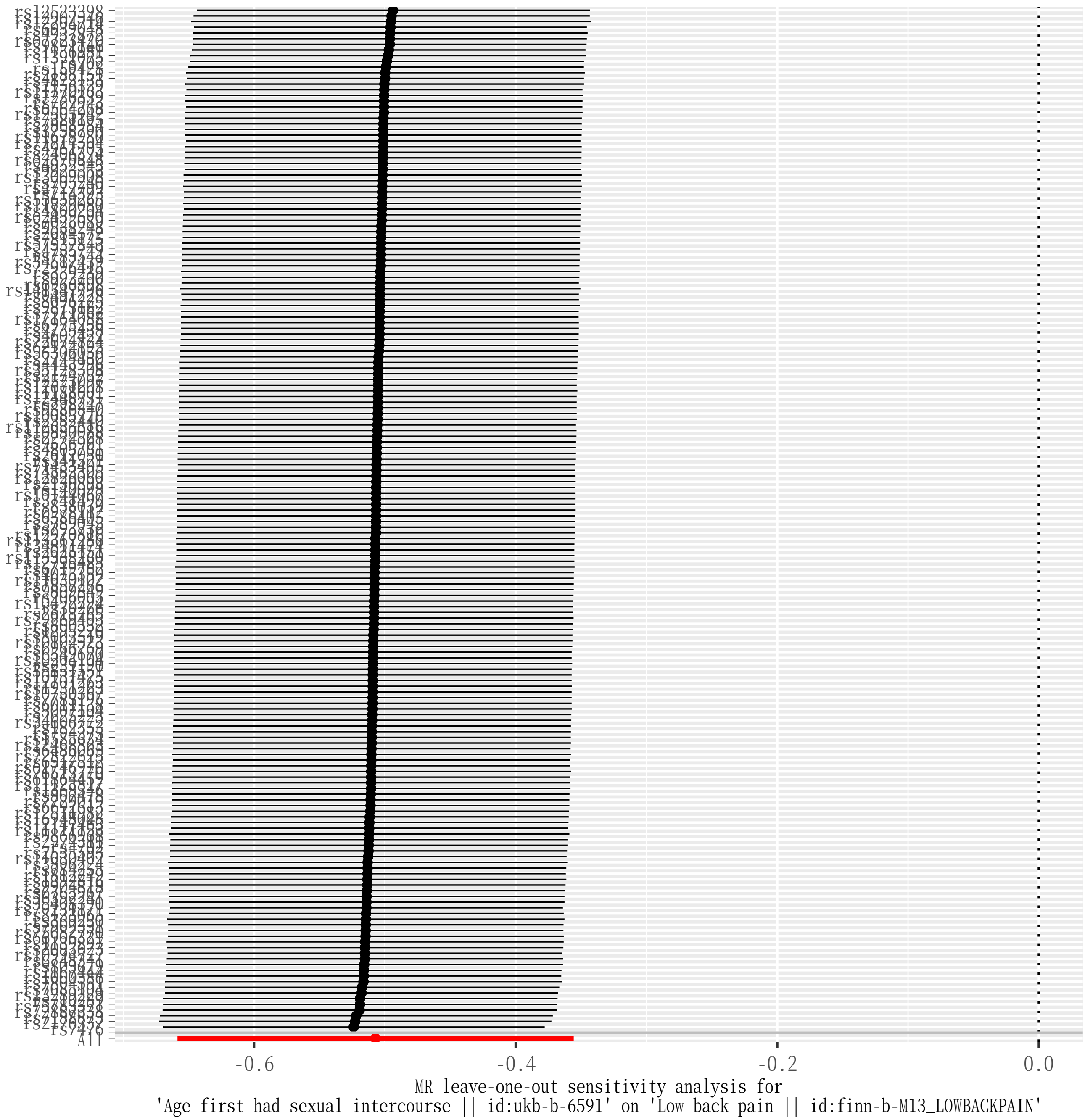
All
-0.6
-0.4
-0.2
0.0
MR leave-one-out sensitivity analysis for
'Age first had sexual intercourse || id:ukb-b-6591' on 'Low back pain || id:finn-b-M13_LOWBACKPAIN'

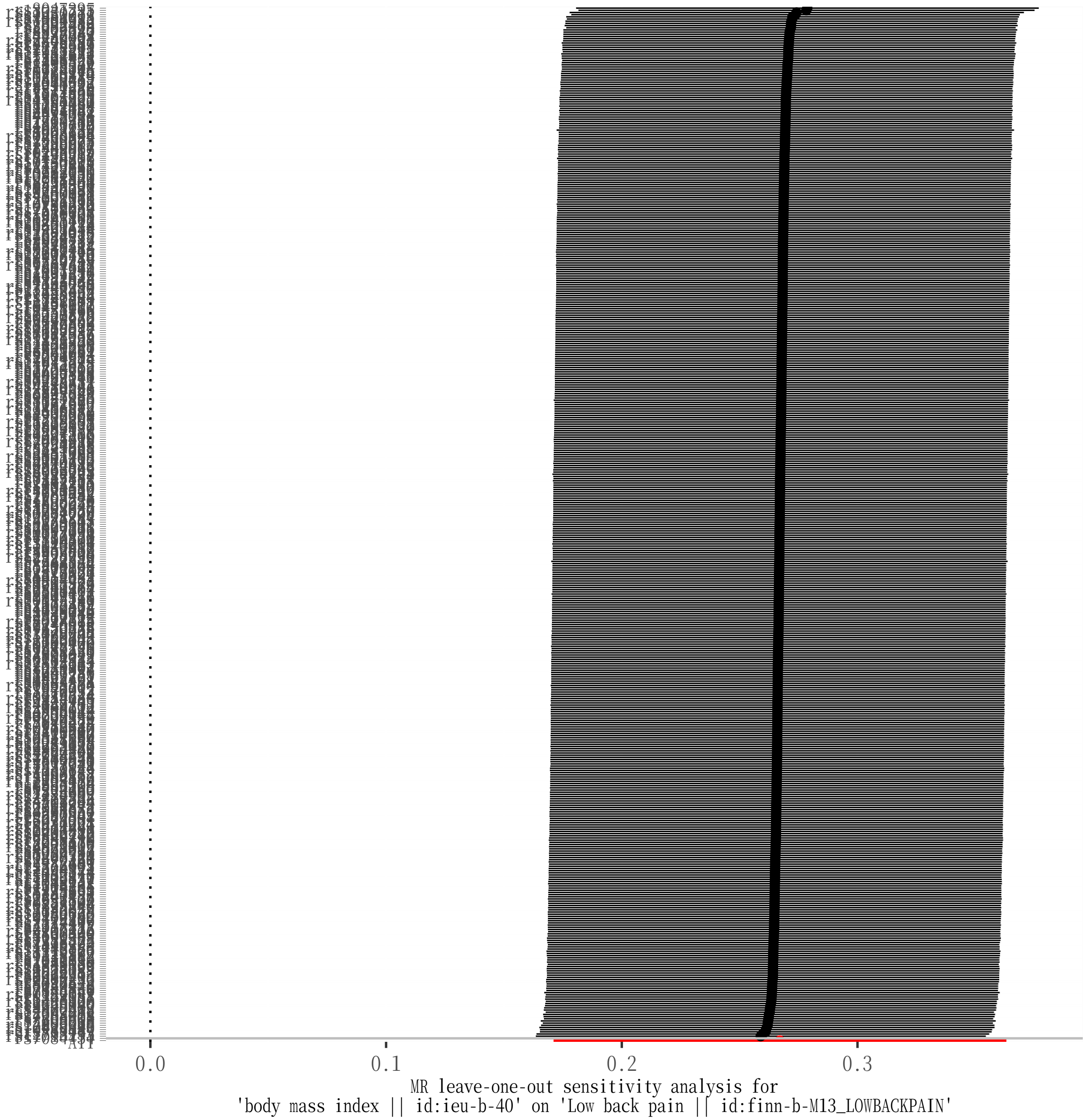
All
0.0
0.1
0.2
0.3
MR leave-one-out sensitivity analysis for
'body mass index || id:ieu-b-40' on 'Low back pain || id:finn-b-M13_LOWBACKPAIN'
