# Supplementary material for: A causal examination of the correlation between hormonal and reproductive factors and low back pain
Source: Front Endocrinol (Lausanne). 2024 May 10;15:1326761. doi: 10.3389/fendo.2024.1326761 (PMC11116661; doi:10.3389/fendo.2024.1326761)
Supplement: Supplementary Figure S4 — Funnel plots for Hormonal and Reproductive Factors. [file DataSheet_4.pdf]

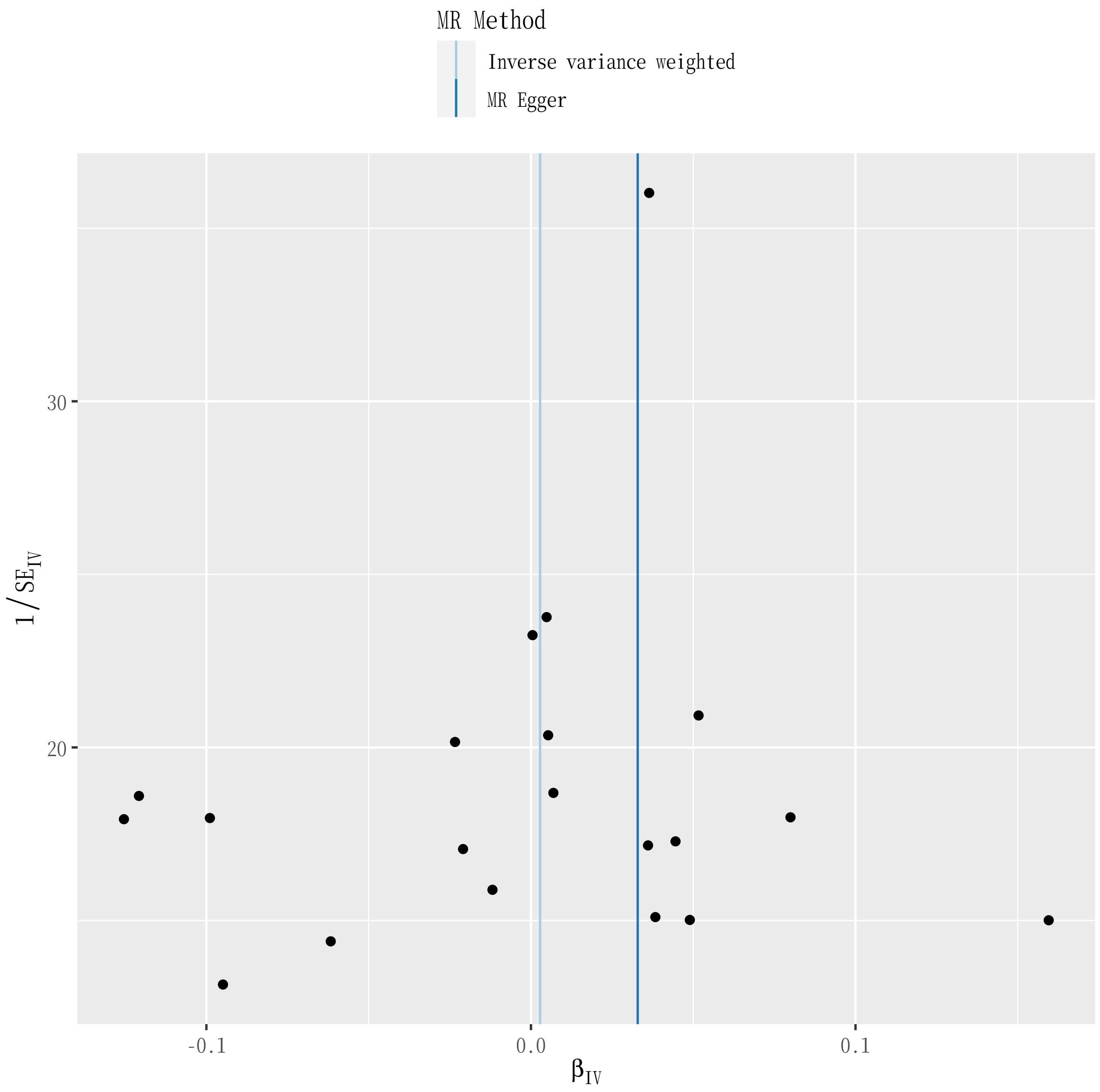
MR Method
Inverse variance weighted
MR Egger
30
20
-0.1
0.0
0.1
1/SE_IV
β_IV

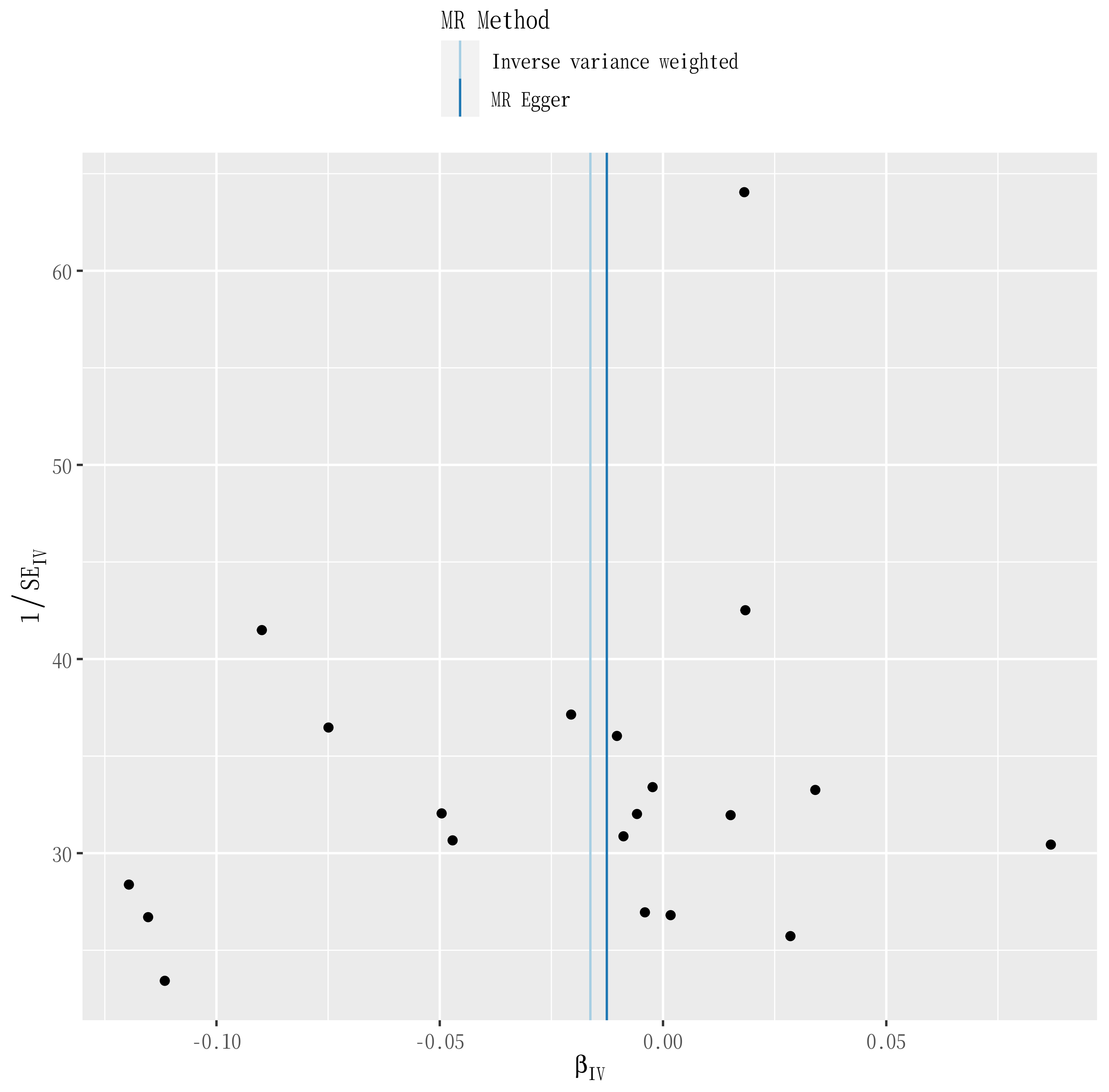
MR Method
Inverse variance weighted
MR Egger
60
50
40
30
1/SE_IV
-0.10
-0.05
0.00
0.05
β_IV

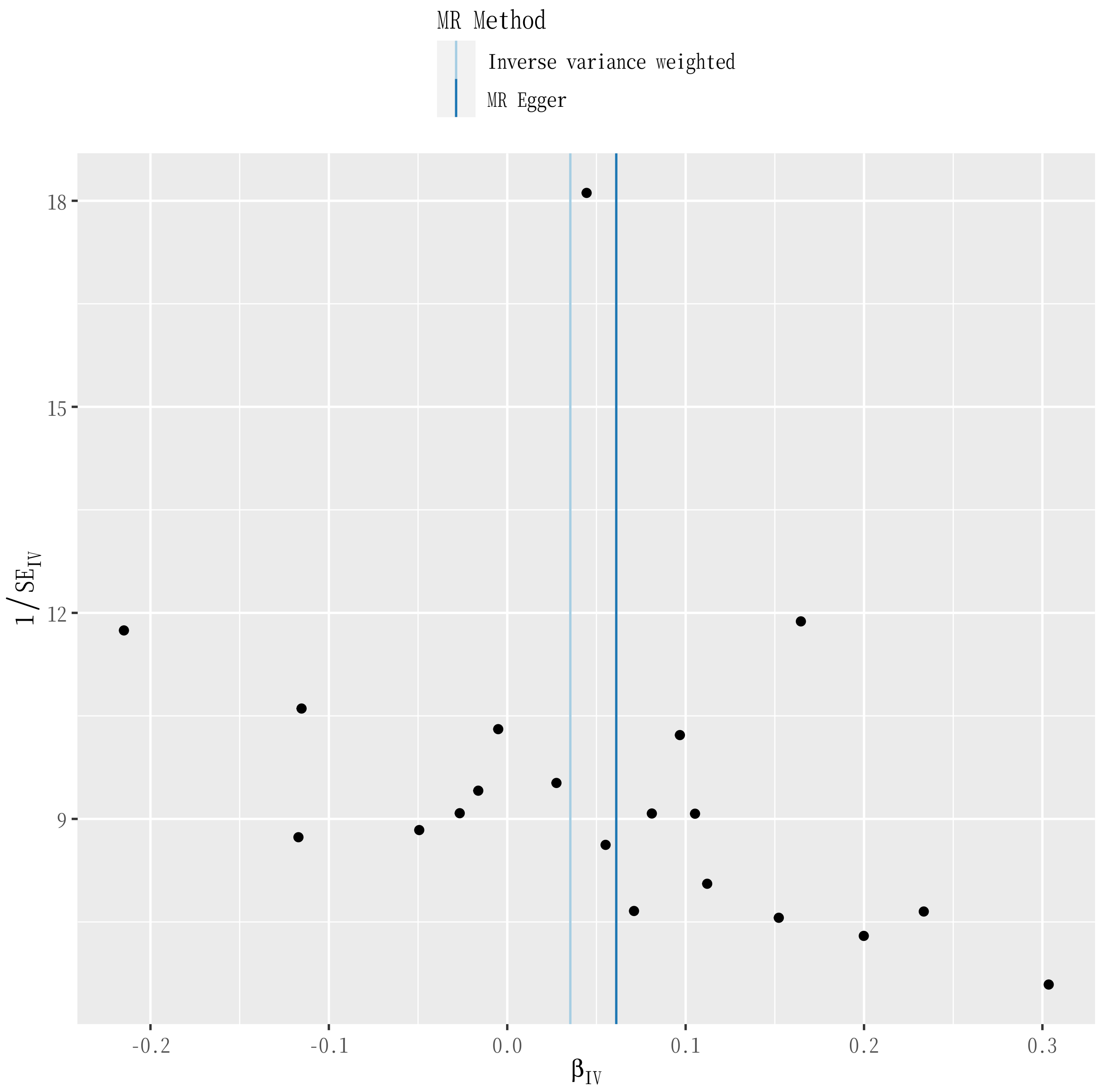
MR Method
Inverse variance weighted
MR Egger
18
15
12
9
$1/SE_{IV}$
-0.2
-0.1
0.0
0.1
0.2
0.3
β_{IV}

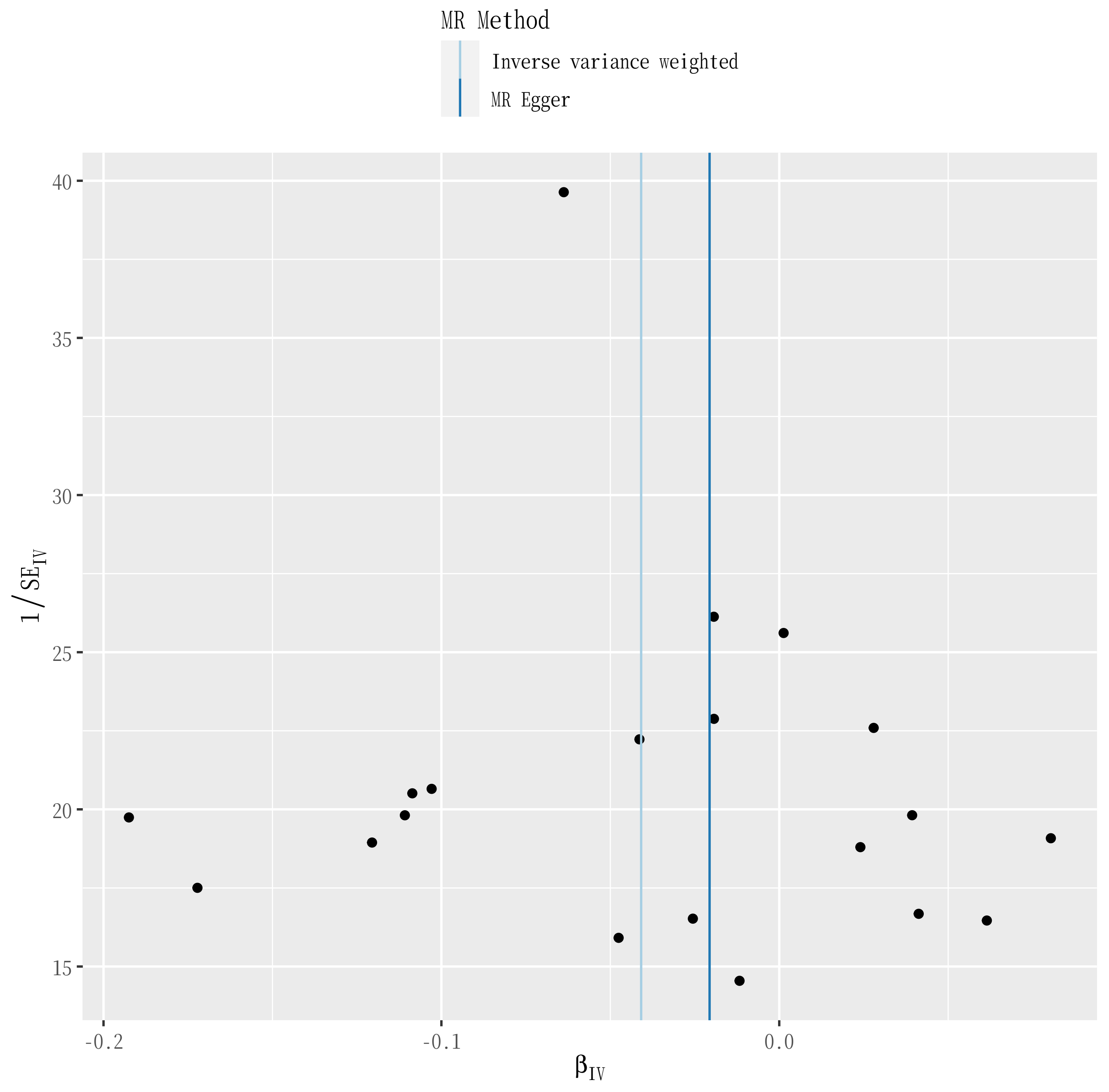

MR Method
Inverse variance weighted
MR Egger
40
35
30
25
20
15
1/SE_IV
-0.2
-0.1
0.0
β_IV

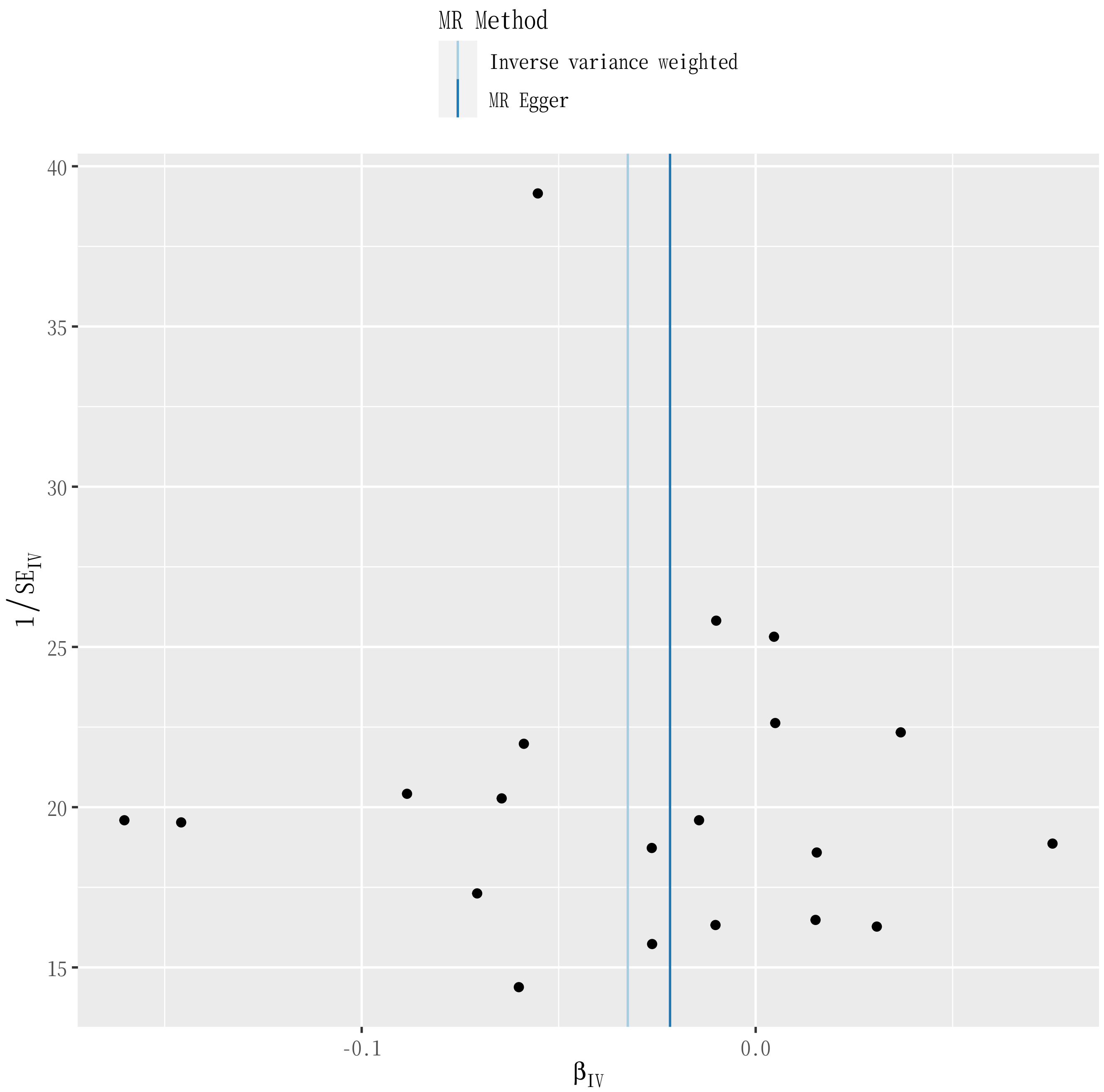

MR Method
Inverse variance weighted
MR Egger
40
35
30
25
20
15
1/SE_IV
-0.1
0.0
β_IV

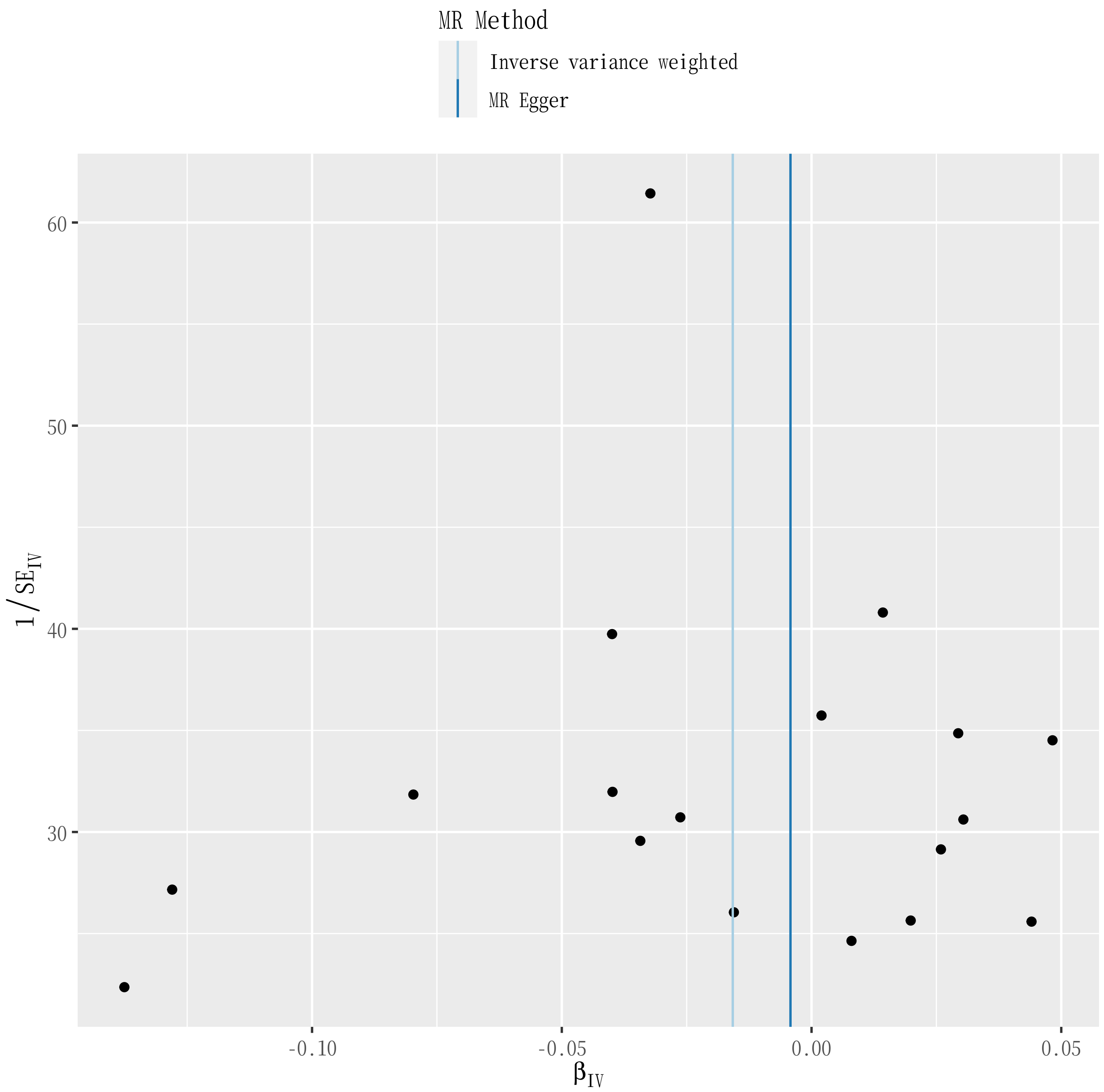
MR Method
Inverse variance weighted
MR Egger
60
50
40
30
-0.10
-0.05
0.00
0.05
β_{IV}
$1/SE_{IV}$

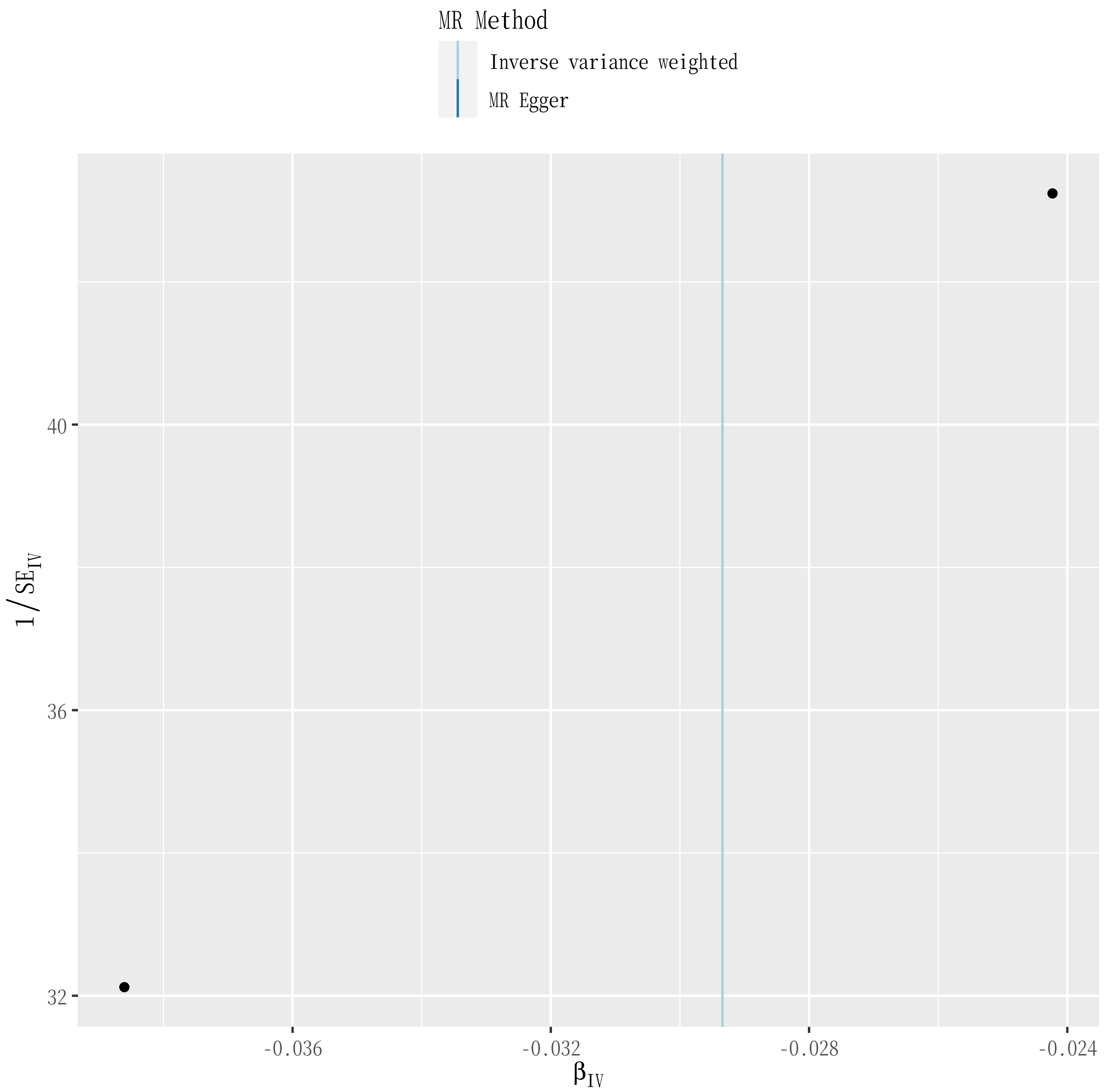

MR Method
Inverse variance weighted
MR Egger
40
36
32
$1/SE_{IV}$
-0.036
-0.032
-0.028
-0.024
β_{IV}
