# Supplementary material for: A causal examination of the correlation between hormonal and reproductive factors and low back pain
Source: Front Endocrinol (Lausanne). 2024 May 10;15:1326761. doi: 10.3389/fendo.2024.1326761 (PMC11116661; doi:10.3389/fendo.2024.1326761)
Supplement: Supplementary Figure S5 — reversed MR leave-one-out sensitivity analysis. [file DataSheet_5.pdf]

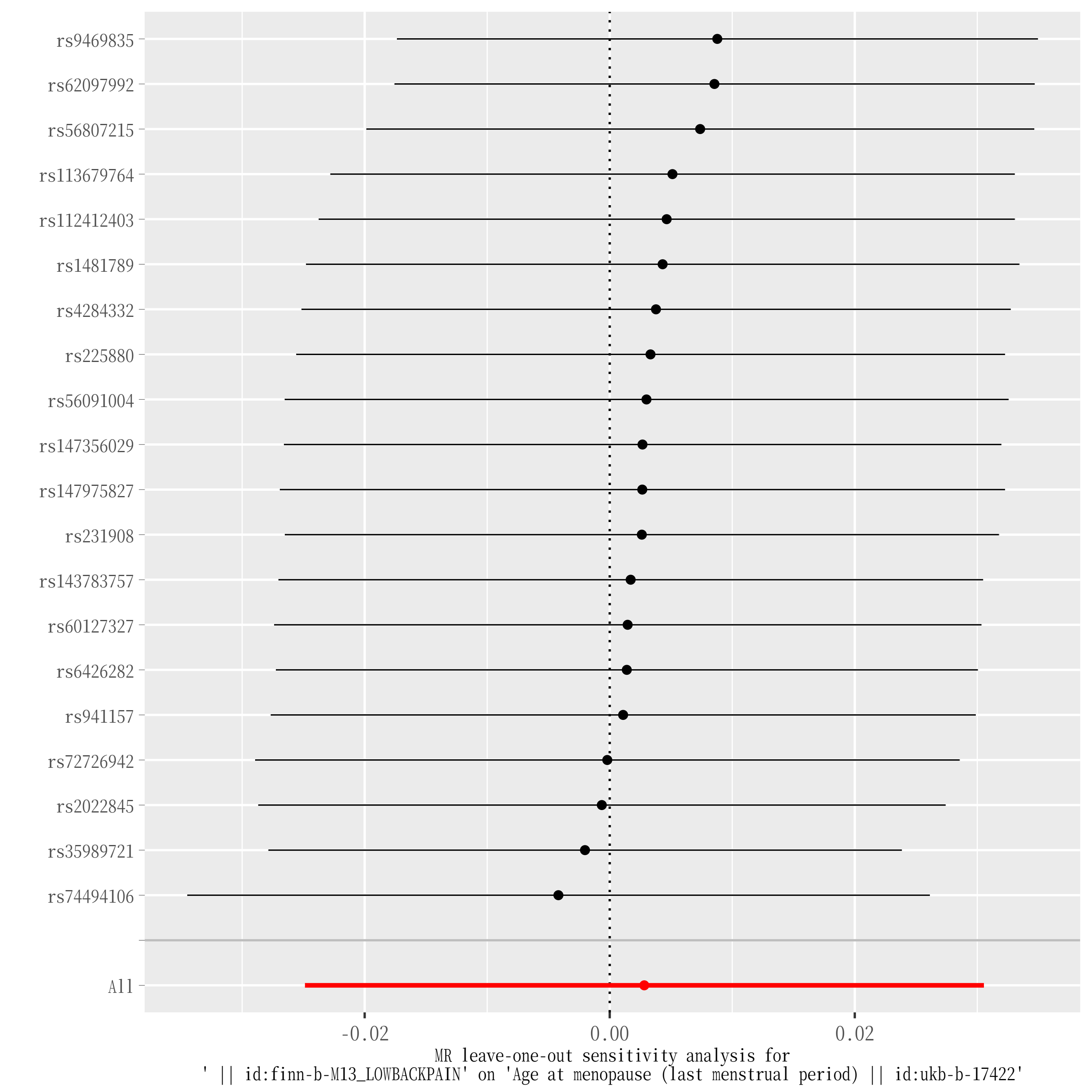

rs9469835
rs62097992
rs56807215
rs113679764
rs112412403
rs1481789
rs4284332
rs225880
rs56091004
rs147356029
rs147975827
rs231908
rs143783757
rs60127327
rs6426282
rs941157
rs72726942
rs2022845
rs35989721
rs74494106
All
-0.02
0.00
0.02
MR leave-one-out sensitivity analysis for
' || id:finn-b-M13_LOWBACKPAIN' on 'Age at menopause (last menstrual period) || id:ukb-b-17422'

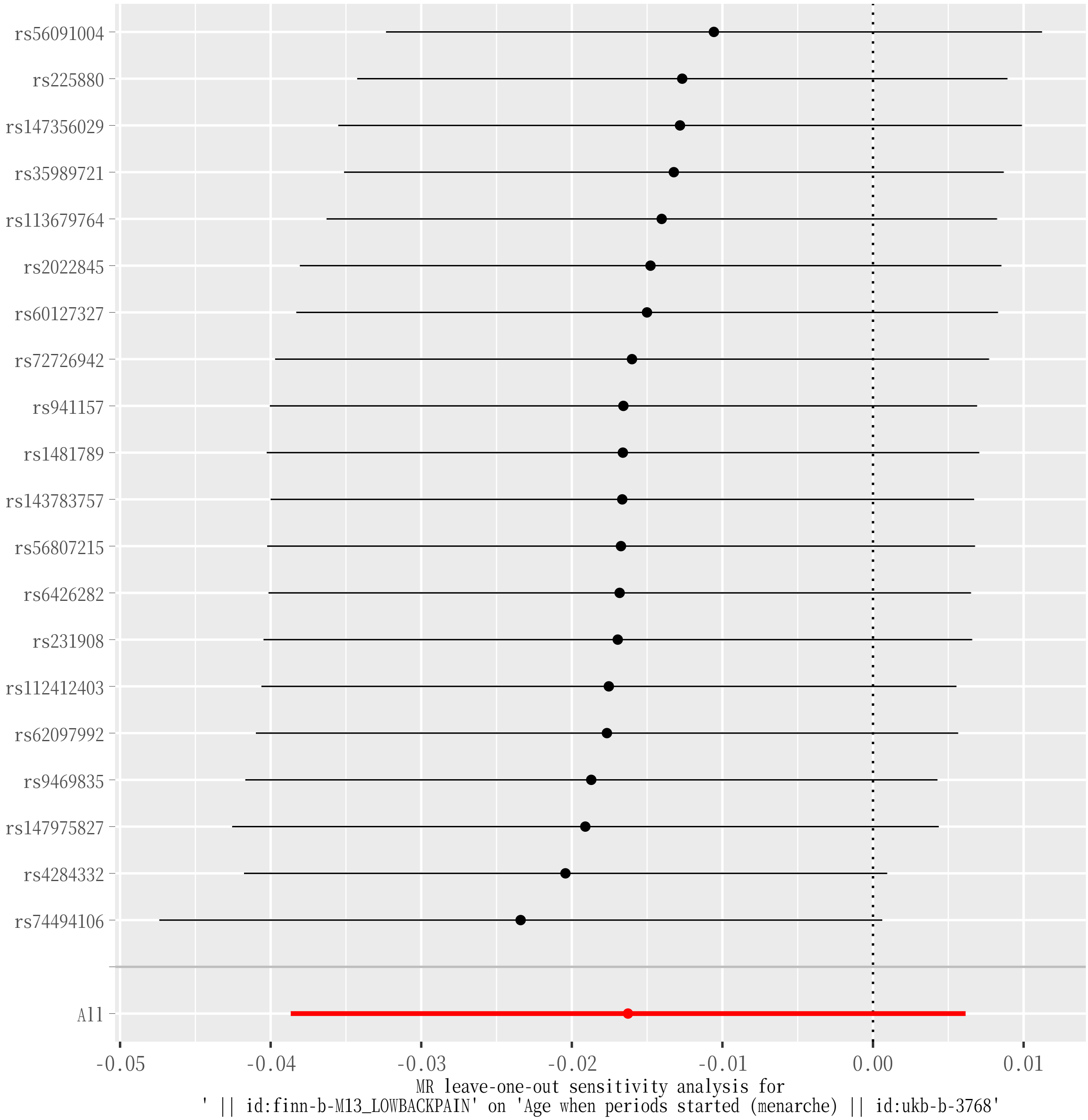
rs56091004
rs225880
rs147356029
rs35989721
rs113679764
rs2022845
rs60127327
rs72726942
rs941157
rs1481789
rs143783757
rs56807215
rs6426282
rs231908
rs112412403
rs62097992
rs9469835
rs147975827
rs4284332
rs74494106
All
-0.05
-0.04
-0.03
-0.02
-0.01
0.00
0.01
MR leave-one-out sensitivity analysis for
' || id:finn-b-M13_LOWBACKPAIN' on 'Age when periods started (menarche) || id:ukb-b-3768'

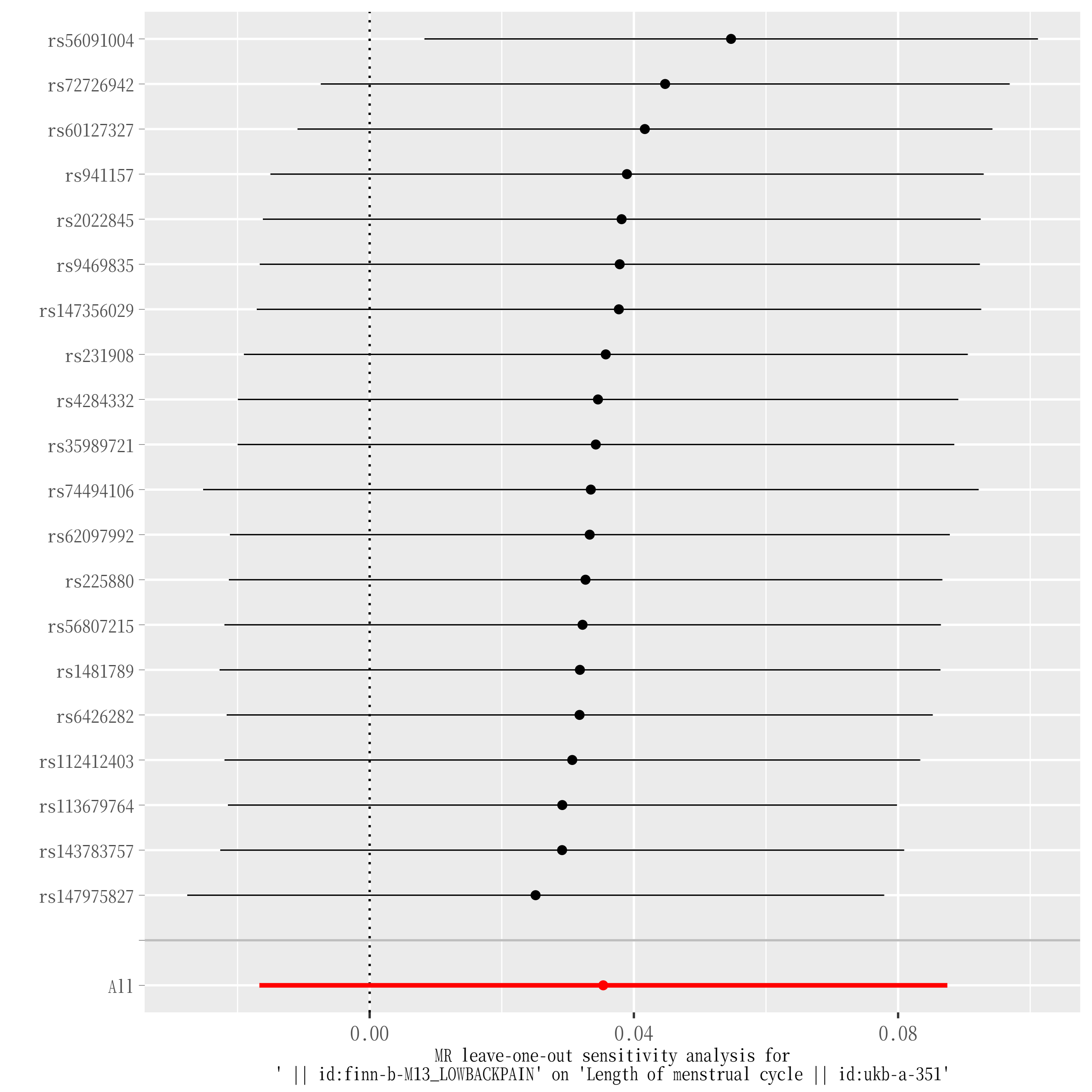
rs56091004
rs72726942
rs60127327
rs941157
rs2022845
rs9469835
rs147356029
rs231908
rs4284332
rs35989721
rs74494106
rs62097992
rs225880
rs56807215
rs1481789
rs6426282
rs112412403
rs113679764
rs143783757
rs147975827
All
0.00
0.04
0.08
MR leave-one-out sensitivity analysis for
' || id:finn-b-M13_LOWBACKPAIN' on 'Length of menstrual cycle || id:ukb-a-351'

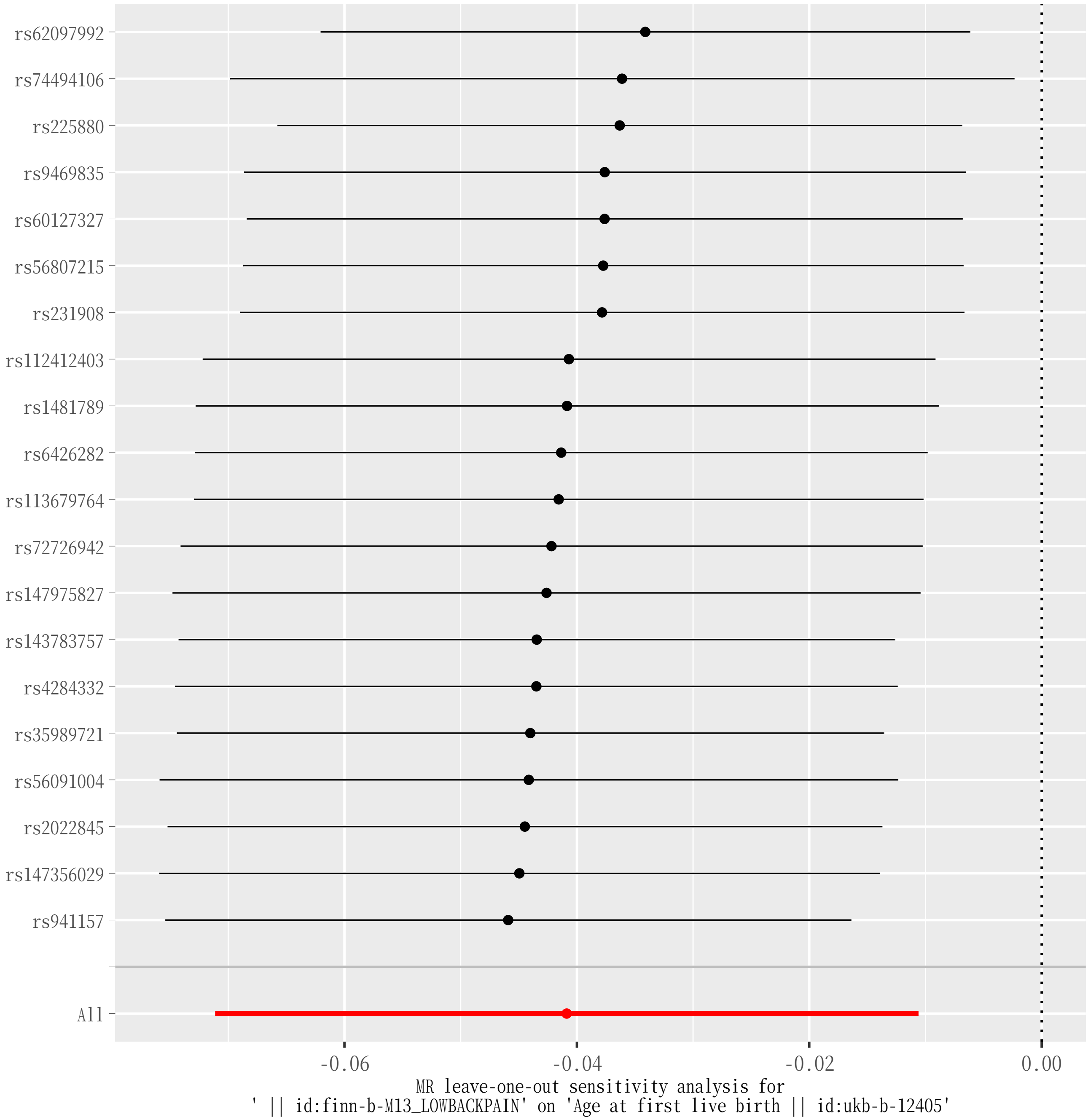
rs62097992
rs74494106
rs225880
rs9469835
rs60127327
rs56807215
rs231908
rs112412403
rs1481789
rs6426282
rs113679764
rs72726942
rs147975827
rs143783757
rs4284332
rs35989721
rs56091004
rs2022845
rs147356029
rs941157
All
-0.06
-0.04
-0.02
0.00
MR leave-one-out sensitivity analysis for
' || id:finn-b-M13_LOWBACKPAIN' on 'Age at first live birth || id:ukb-b-12405'

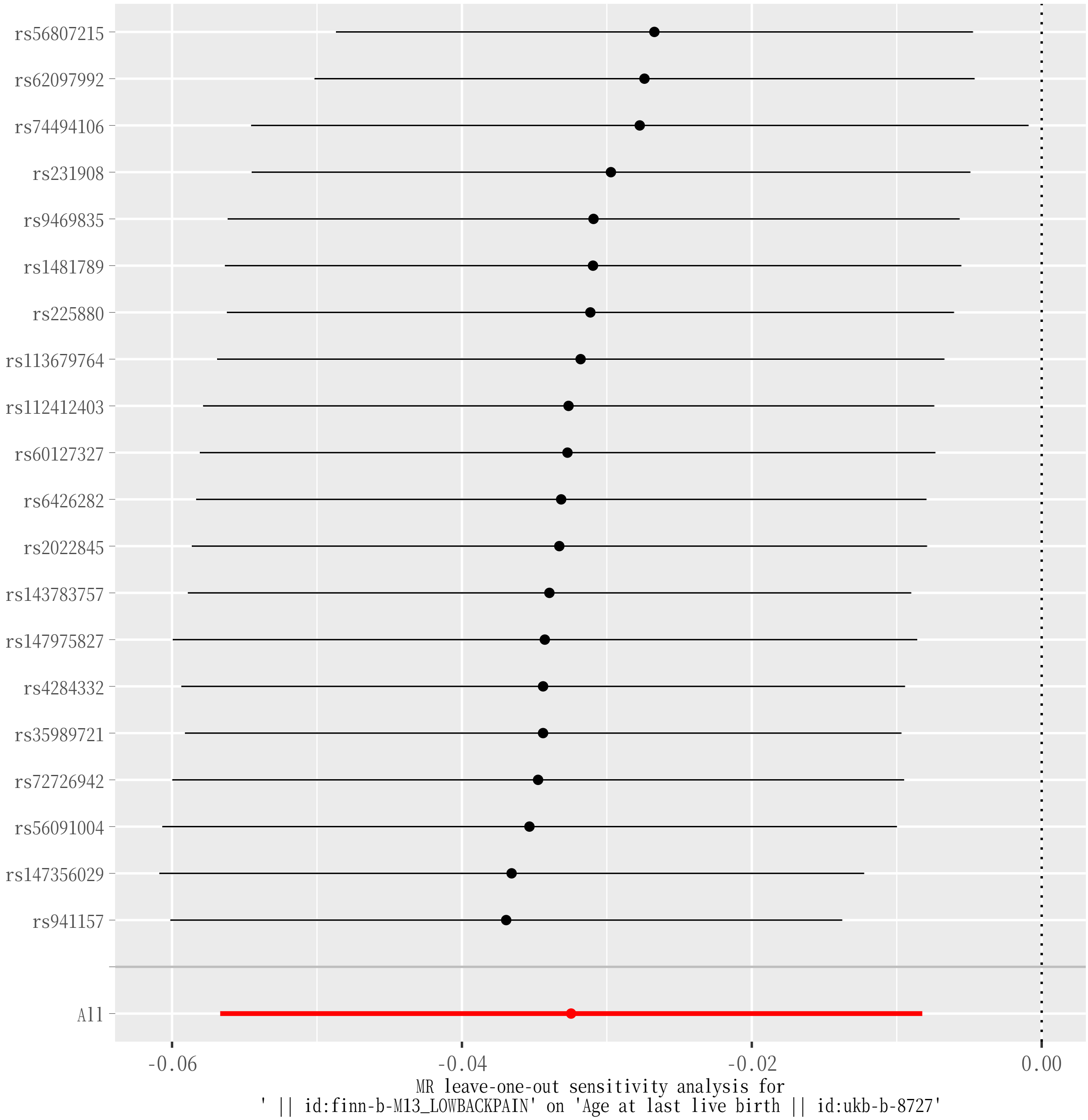

rs56807215
rs62097992
rs74494106
rs231908
rs9469835
rs1481789
rs225880
rs113679764
rs112412403
rs60127327
rs6426282
rs2022845
rs143783757
rs147975827
rs4284332
rs35989721
rs72726942
rs56091004
rs147356029
rs941157
All
-0.06
-0.04
-0.02
0.00
MR leave-one-out sensitivity analysis for
' || id:finn-b-M13_LOWBACKPAIN' on 'Age at last live birth || id:ukb-b-8727'

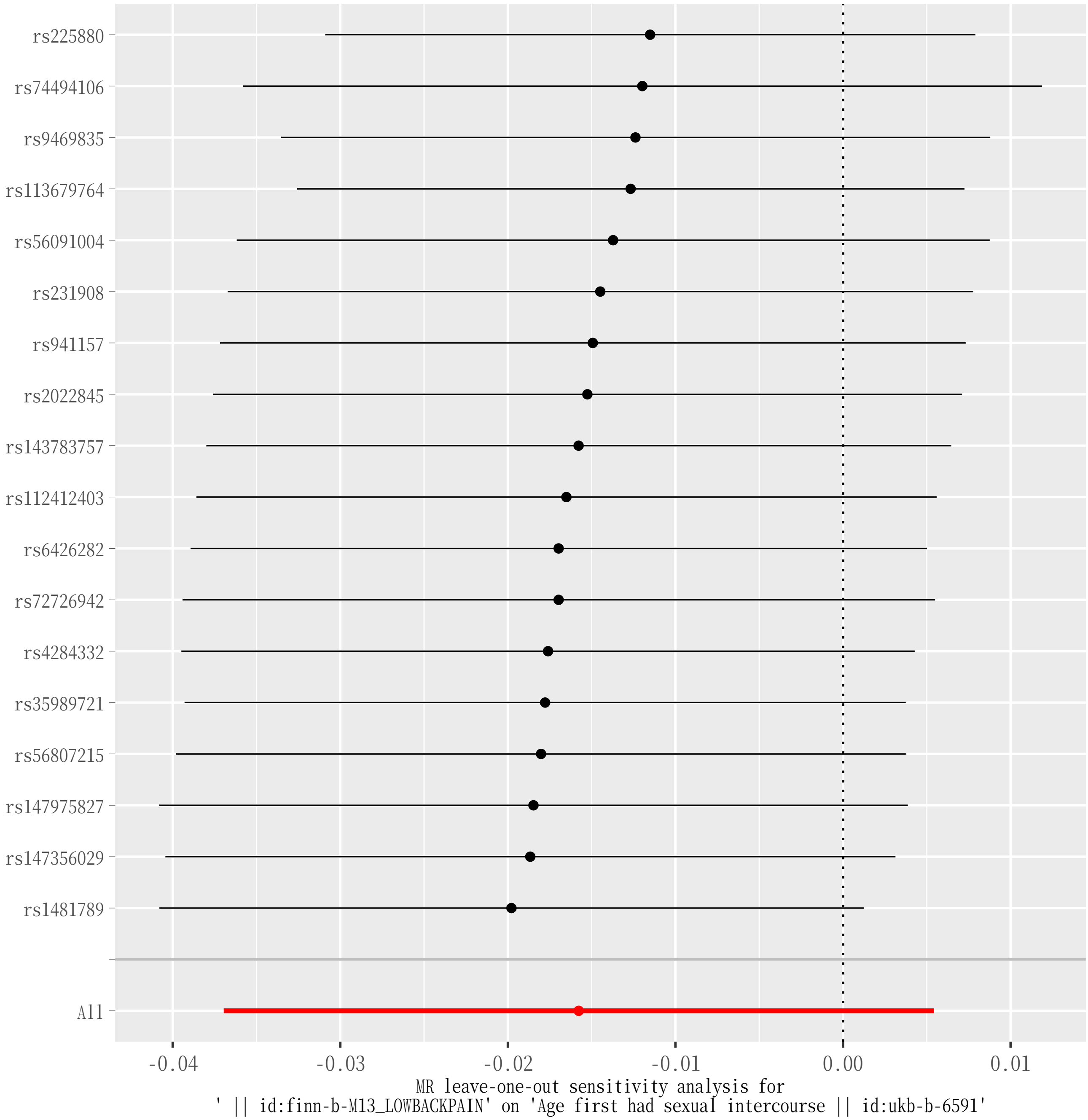
rs225880
rs74494106
rs9469835
rs113679764
rs56091004
rs231908
rs941157
rs2022845
rs143783757
rs112412403
rs6426282
rs72726942
rs4284332
rs35989721
rs56807215
rs147975827
rs147356029
rs1481789
All
-0.04
-0.03
-0.02
-0.01
0.00
0.01
MR leave-one-out sensitivity analysis for
' || id:finn-b-M13_LOWBACKPAIN' on 'Age first had sexual intercourse || id:ukb-b-6591'

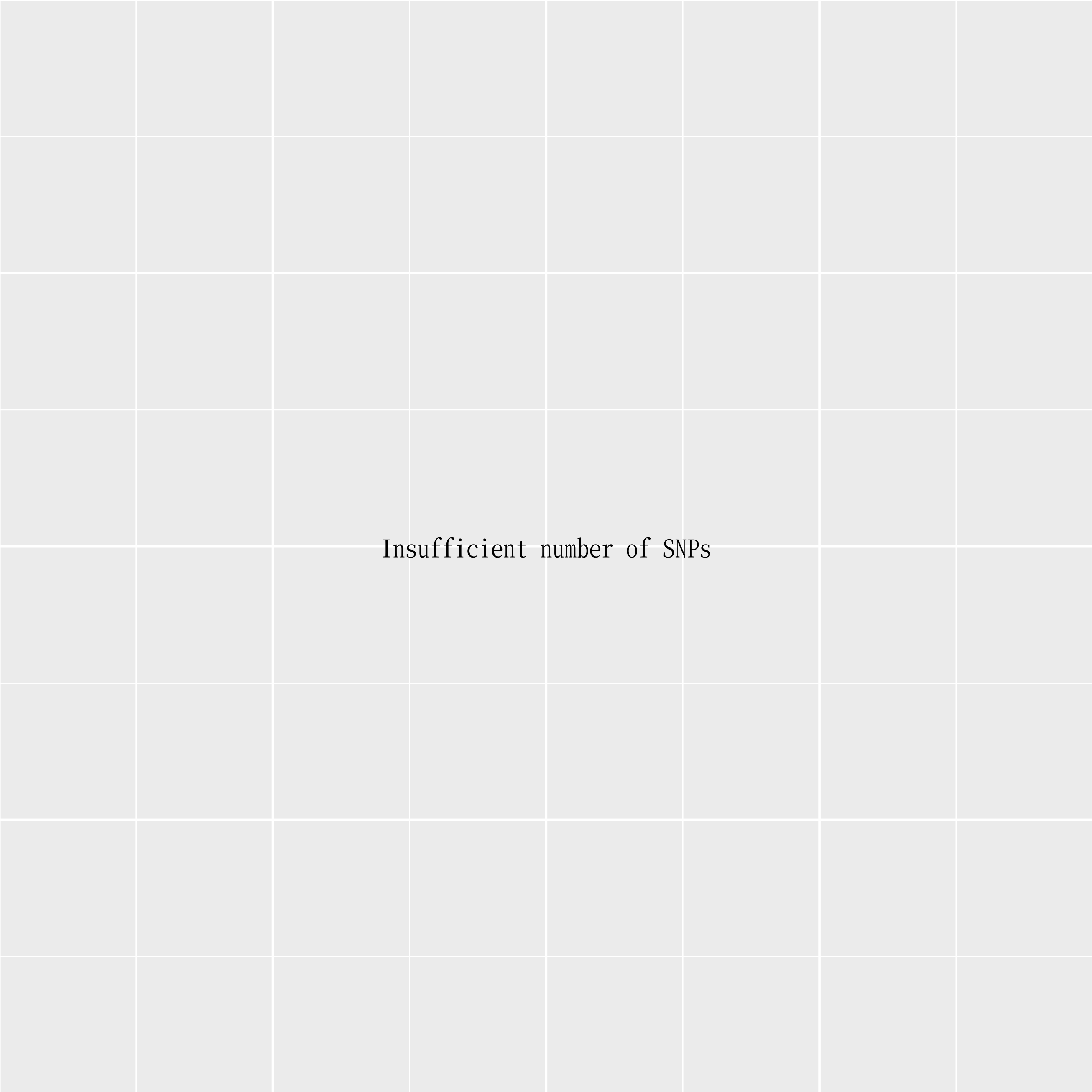
Insufficient number of SNPs
