# Supplementary material for: A causal examination of the correlation between hormonal and reproductive factors and low back pain
Source: Front Endocrinol (Lausanne). 2024 May 10;15:1326761. doi: 10.3389/fendo.2024.1326761 (PMC11116661; doi:10.3389/fendo.2024.1326761)
Supplement: Supplementary Figure S6 — Scatter plot for Hormonal and Reproductive Factors. [file DataSheet_6.pdf]

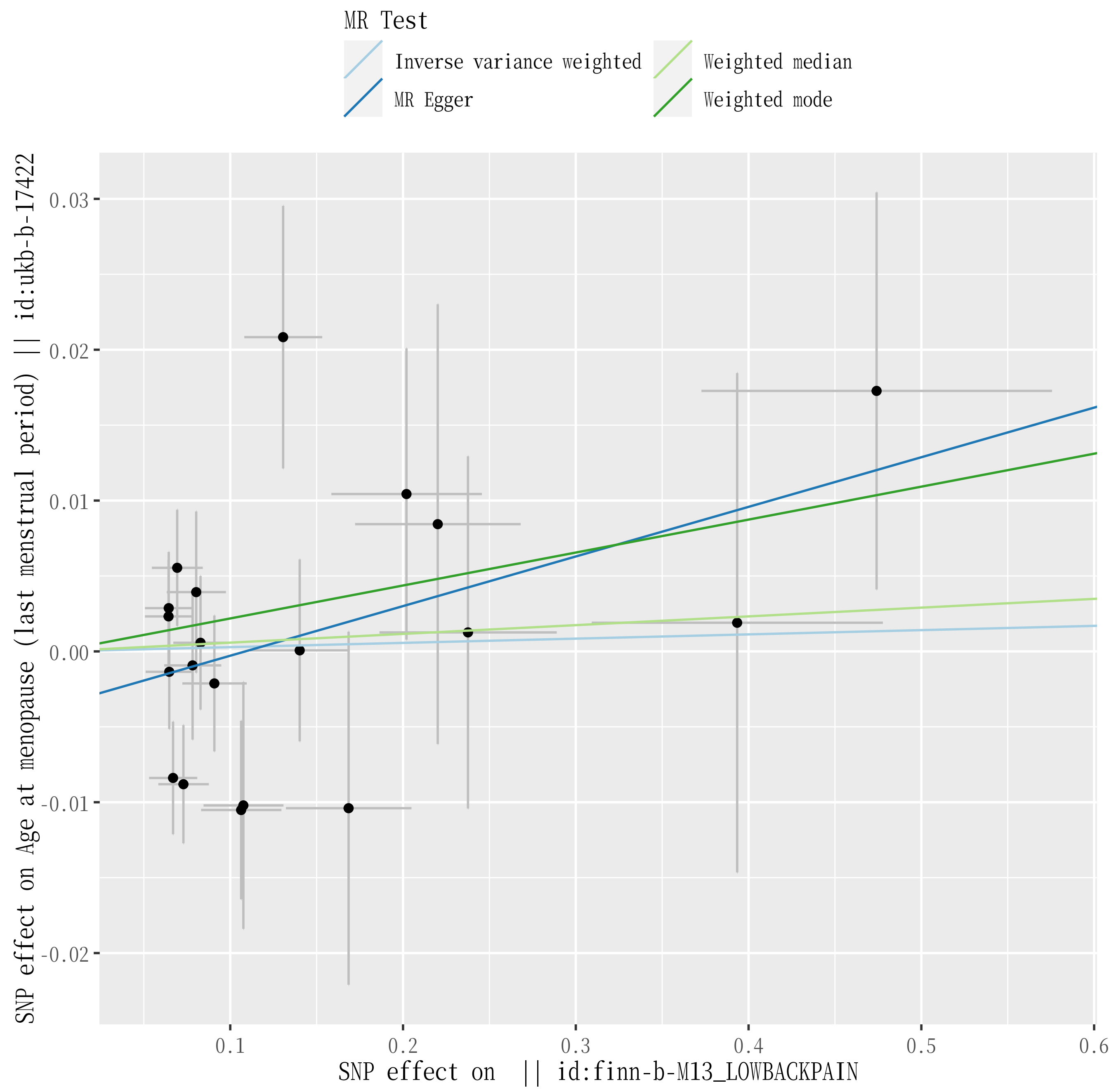

MR Test
Inverse variance weighted
MR Egger
Weighted median
Weighted mode
0.03
0.02
0.01
0.00
-0.01
-0.02
SNP effect on Age at menopause (last menstrual period) || id:ukb-b-17422
0.1
0.2
0.3
0.4
0.5
0.6
SNP effect on || id:finn-b-M13_LOWBACKPAIN

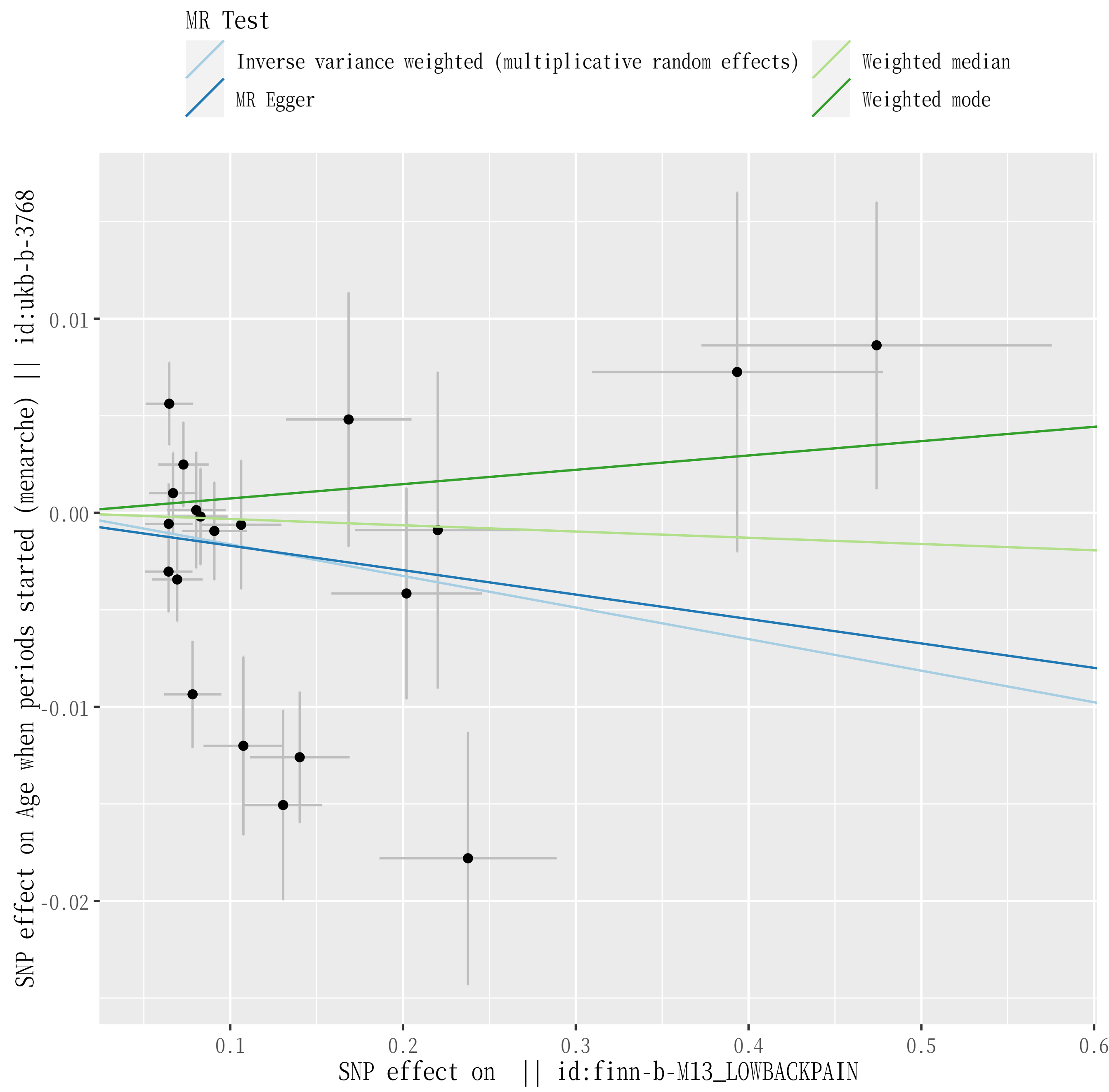
MR Test
Inverse variance weighted (multiplicative random effects)
MR Egger
Weighted median
Weighted mode
0.01
0.00
-0.01
-0.02
0.1
0.2
0.3
0.4
0.5
0.6
SNP effect on Age when periods started (menarche) || id:ukb-b-3768
SNP effect on || id:finn-b-M13_LOWBACKPAIN

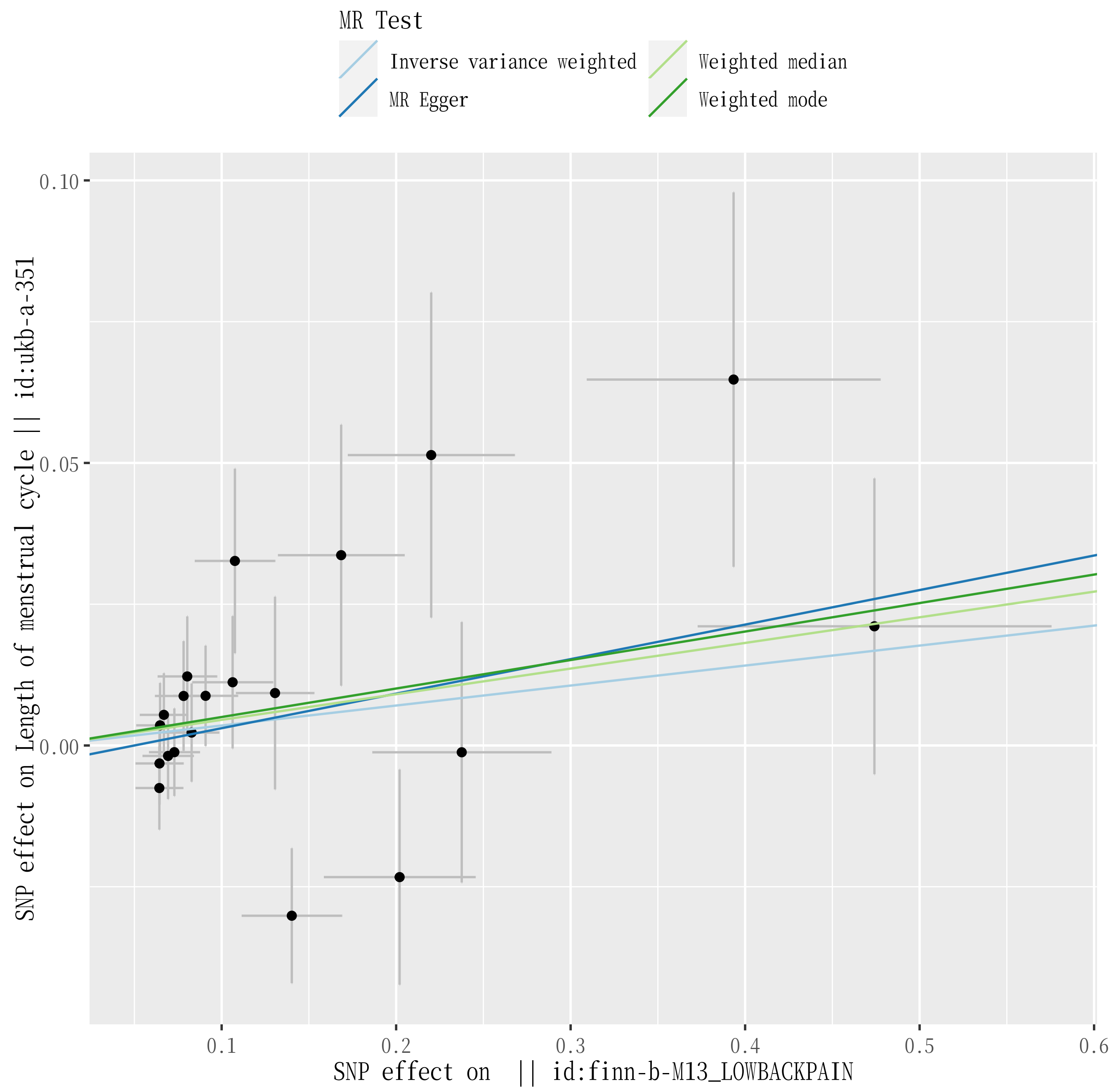
MR Test
Inverse variance weighted
Weighted median
MR Egger
Weighted mode
0.10
0.05
0.00
0.1
0.2
0.3
0.4
0.5
0.6
SNP effect on Length of menstrual cycle || id:ukb-a-351
SNP effect on || id:finn-b-M13_LOWBACKPAIN

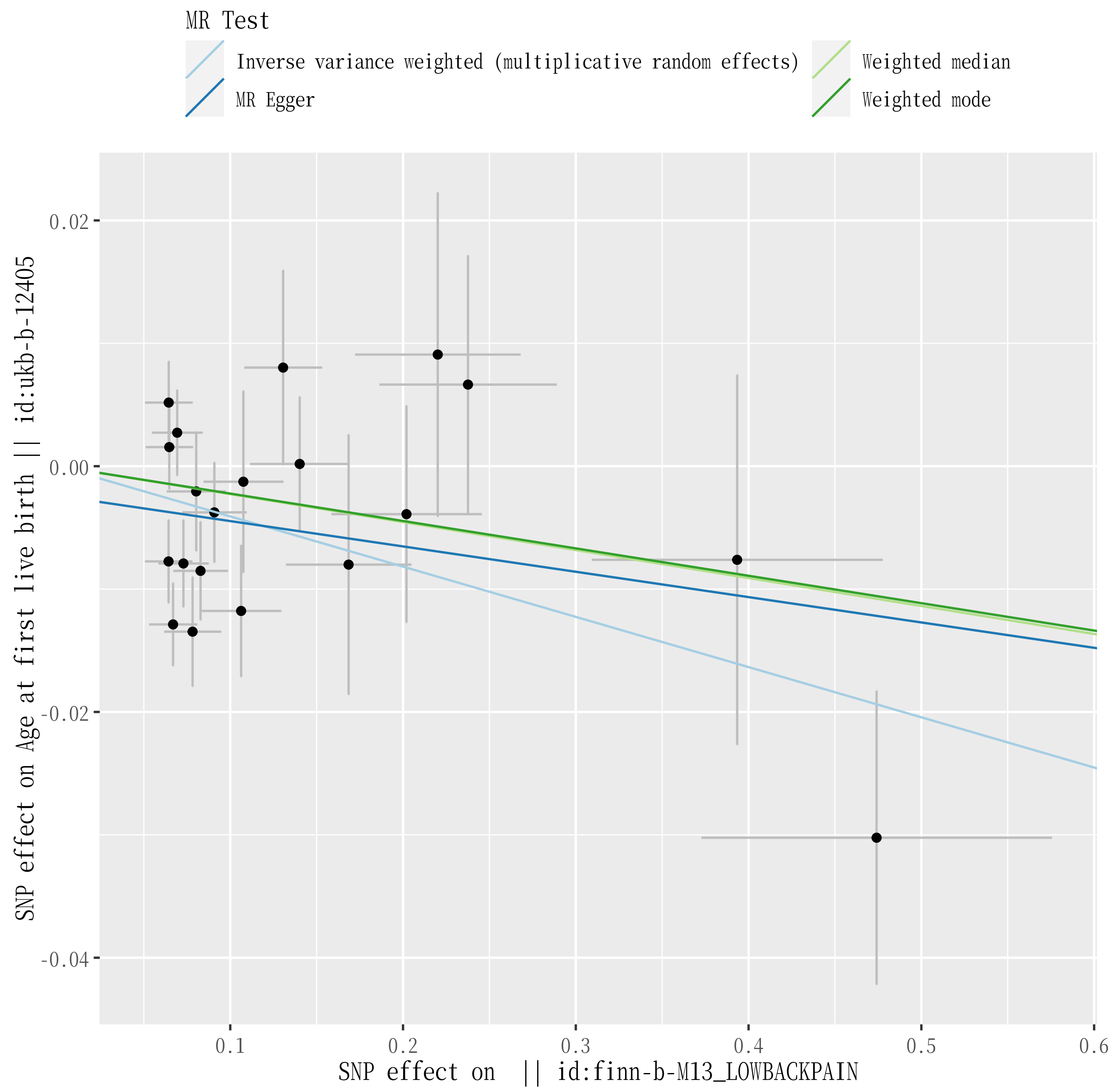
MR Test
Inverse variance weighted (multiplicative random effects)
MR Egger
Weighted median
Weighted mode
0.02
0.00
-0.02
-0.04
0.1
0.2
0.3
0.4
0.5
0.6
SNP effect on Age at first live birth || id:ukb-b-12405
SNP effect on || id:finn-b-M13_LOWBACKPAIN

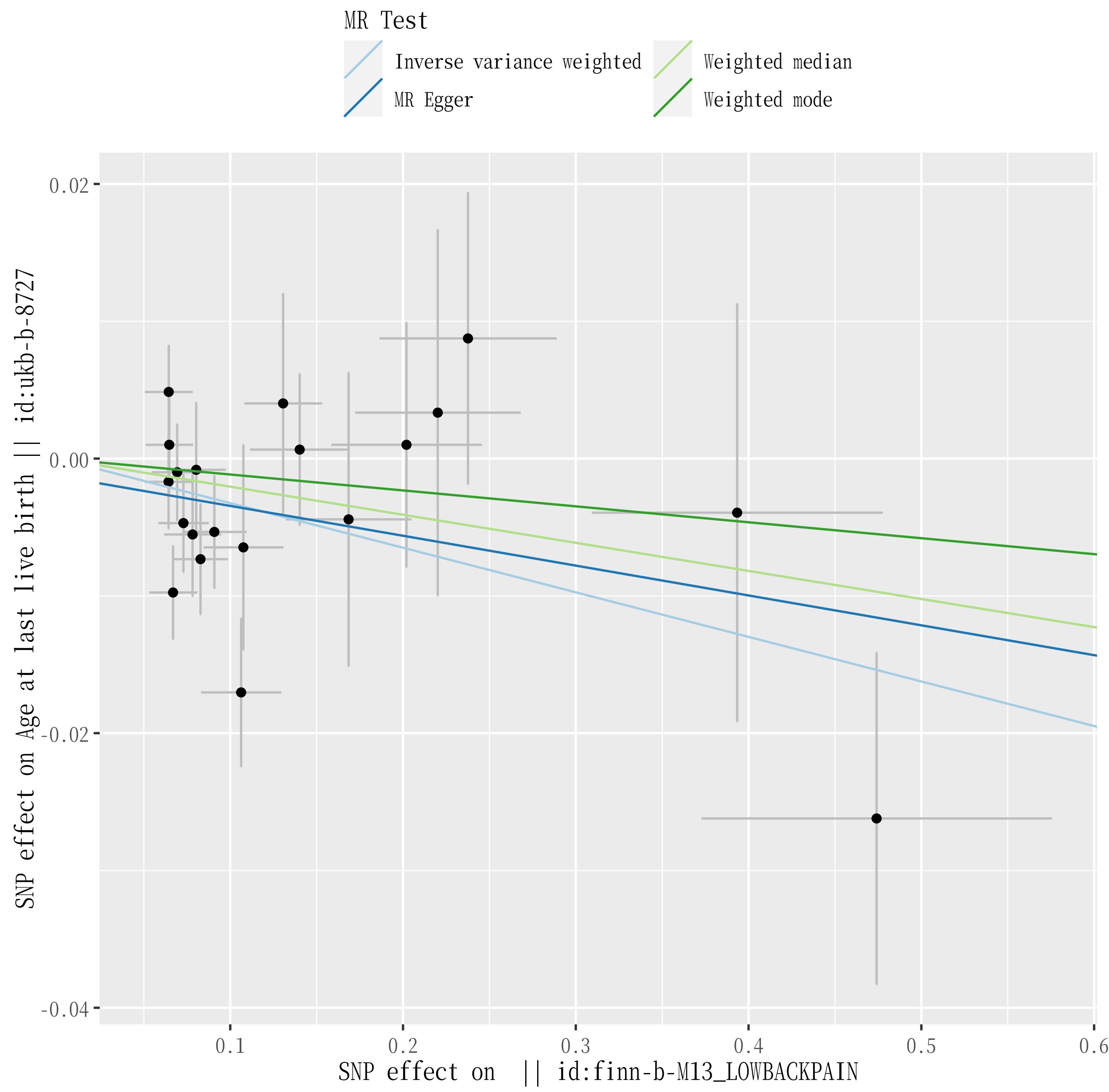
MR Test
Inverse variance weighted
MR Egger
Weighted median
Weighted mode
0.02
0.00
-0.02
-0.04
0.1
0.2
0.3
0.4
0.5
0.6
SNP effect on Age at last live birth || id:ukb-b-8727
SNP effect on || id:finn-b-M13_LOWBACKPAIN

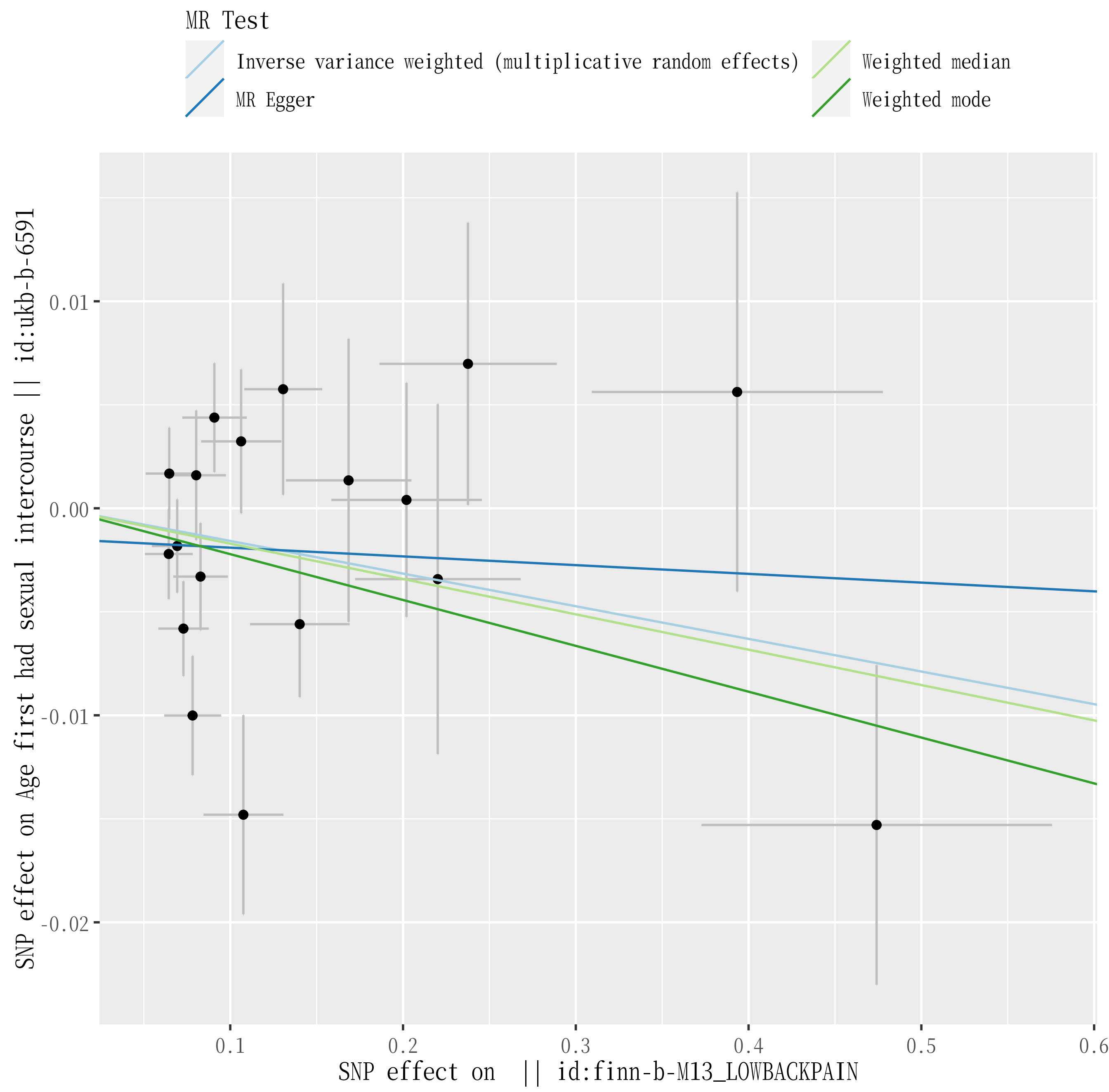

MR Test
Inverse variance weighted (multiplicative random effects)
MR Egger
Weighted median
Weighted mode
0.01
0.00
-0.01
-0.02
SNP effect on Age first had sexual intercourse || id:ukb-b-6591
0.1
0.2
0.3
0.4
0.5
0.6
SNP effect on || id:finn-b-M13_LOWBACKPAIN

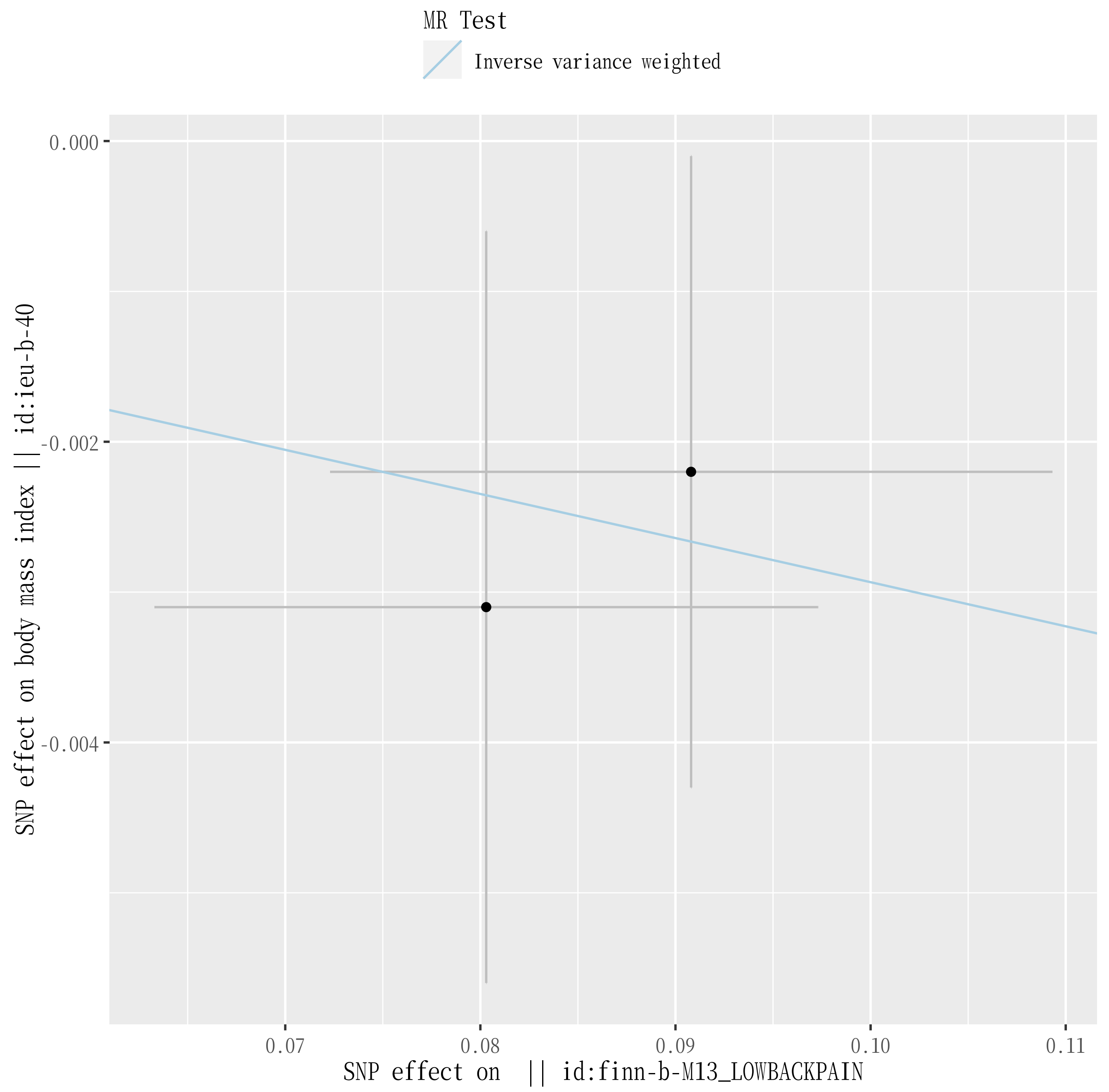
MR Test
Inverse variance weighted
0.000
-0.002
-0.004
0.07
0.08
0.09
0.10
0.11
SNP effect on body mass index || id:ieu-b-40
SNP effect on || id:finn-b-M13_LOWBACKPAIN
