# Supplementary material for: A causal examination of the correlation between hormonal and reproductive factors and low back pain
Source: Front Endocrinol (Lausanne). 2024 May 10;15:1326761. doi: 10.3389/fendo.2024.1326761 (PMC11116661; doi:10.3389/fendo.2024.1326761)
Supplement: Supplementary Figure S7 — Scatter plot for LBP (replication analysis). [file DataSheet_7.pdf]

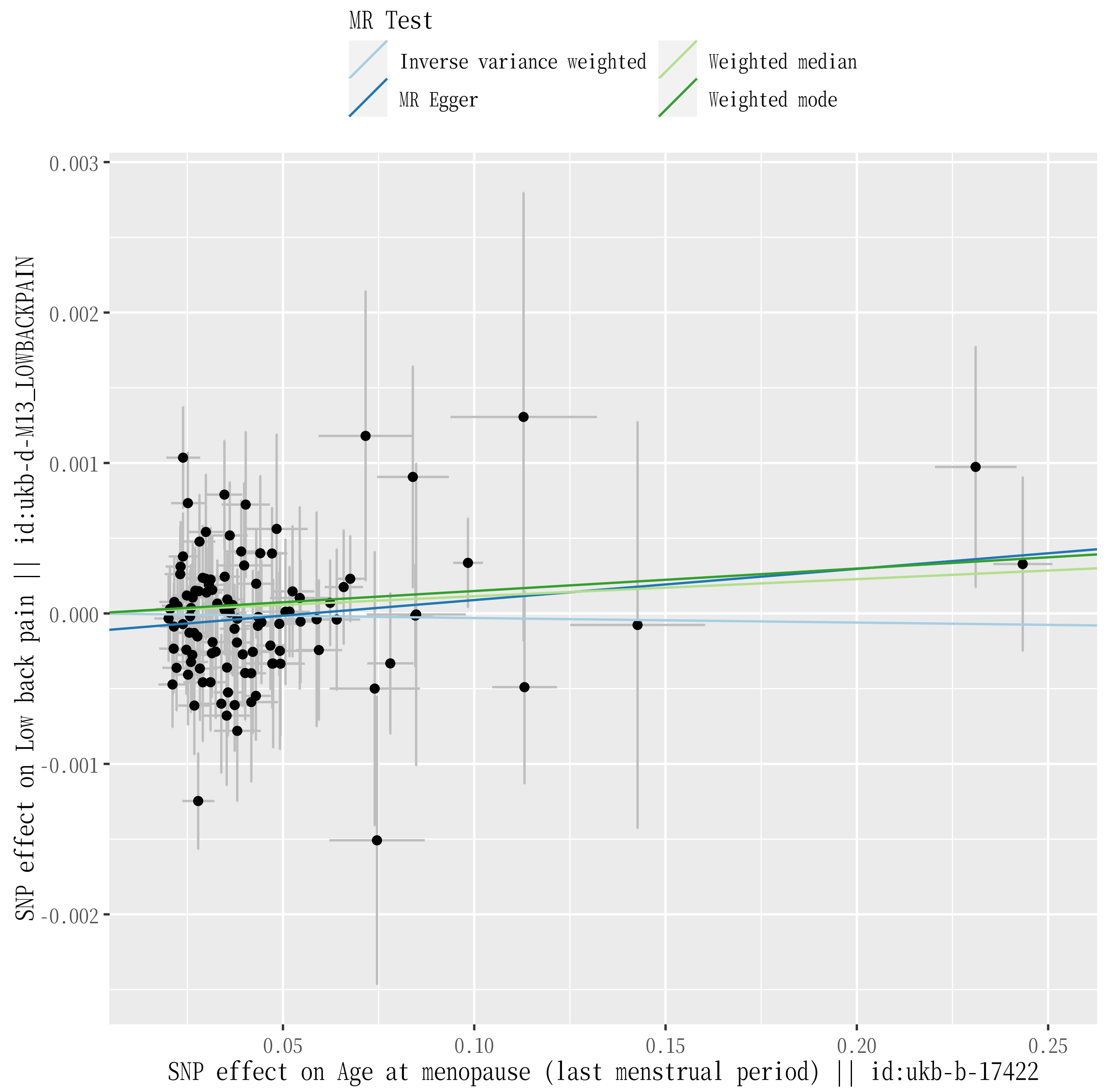
MR Test
Inverse variance weighted
MR Egger
Weighted median
Weighted mode
0.003
0.002
0.001
0.000
-0.001
-0.002
SNP effect on Low back pain || id:ukb-d-M13_LOWBACKPAIN
0.05
0.10
0.15
0.20
0.25
SNP effect on Age at menopause (last menstrual period) || id:ukb-b-17422

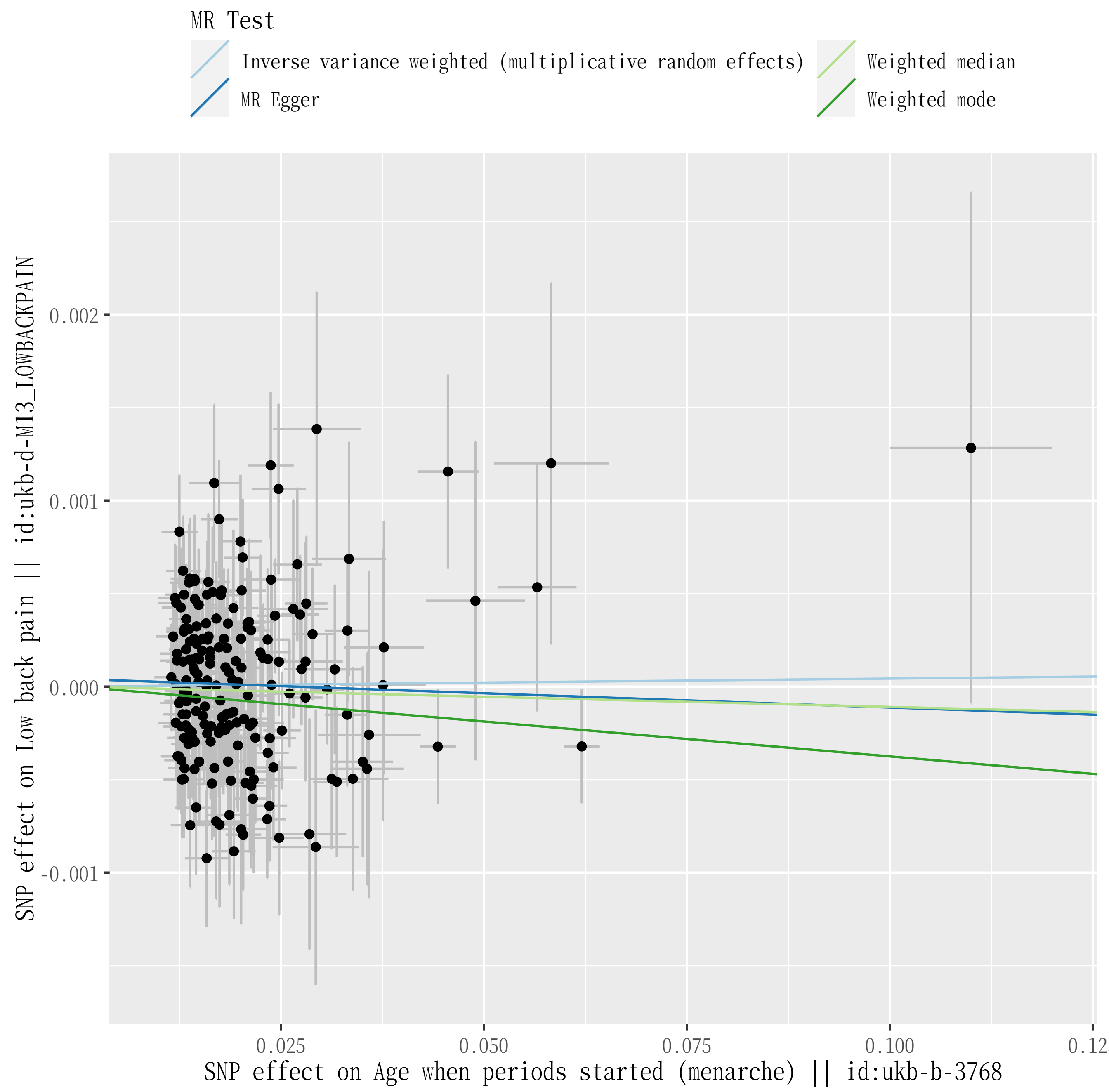
MR Test
Inverse variance weighted (multiplicative random effects)
MR Egger
Weighted median
Weighted mode
0.002
0.001
0.000
-0.001
SNP effect on Low back pain || id:ukb-d-M13_LOWBACKPAIN
0.025
0.050
0.075
0.100
SNP effect on Age when periods started (menarche) || id:ukb-b-3768

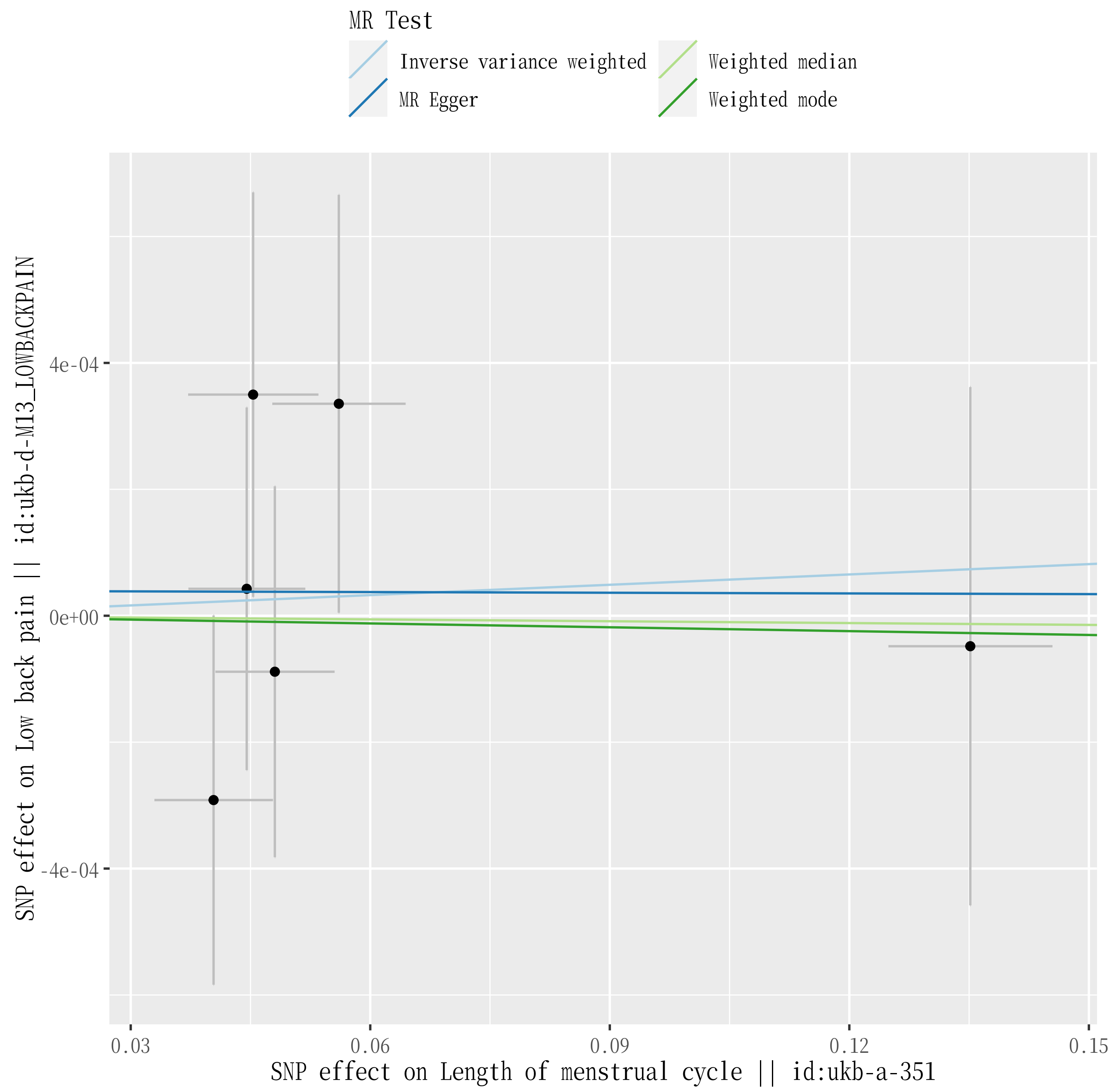
MR Test
Inverse variance weighted
MR Egger
Weighted median
Weighted mode
4e-04
0e+00
-4e-04
0.03
0.06
0.09
0.12
0.15
SNP effect on Length of menstrual cycle || id:ukb-a-351
SNP effect on Low back pain || id:ukb-d-M13_LOWBACKPAIN

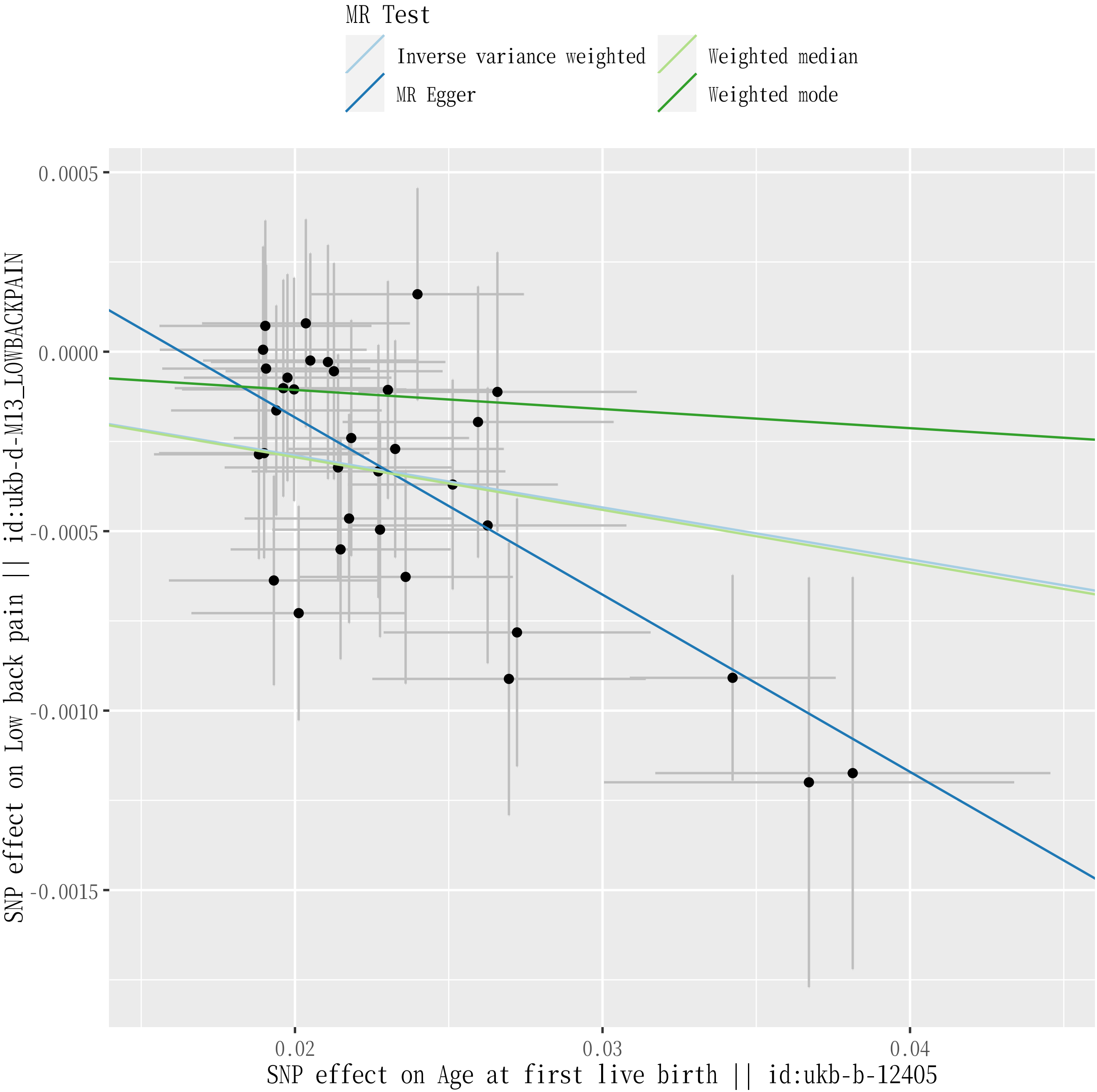
MR Test
Inverse variance weighted
MR Egger
Weighted median
Weighted mode
0.0005
0.0000
-0.0005
-0.0010
-0.0015
0.02
0.03
0.04
SNP effect on Age at first live birth || id:ukb-b-12405
SNP effect on Low back pain || id:ukb-d-M13_LOWBACKPAIN

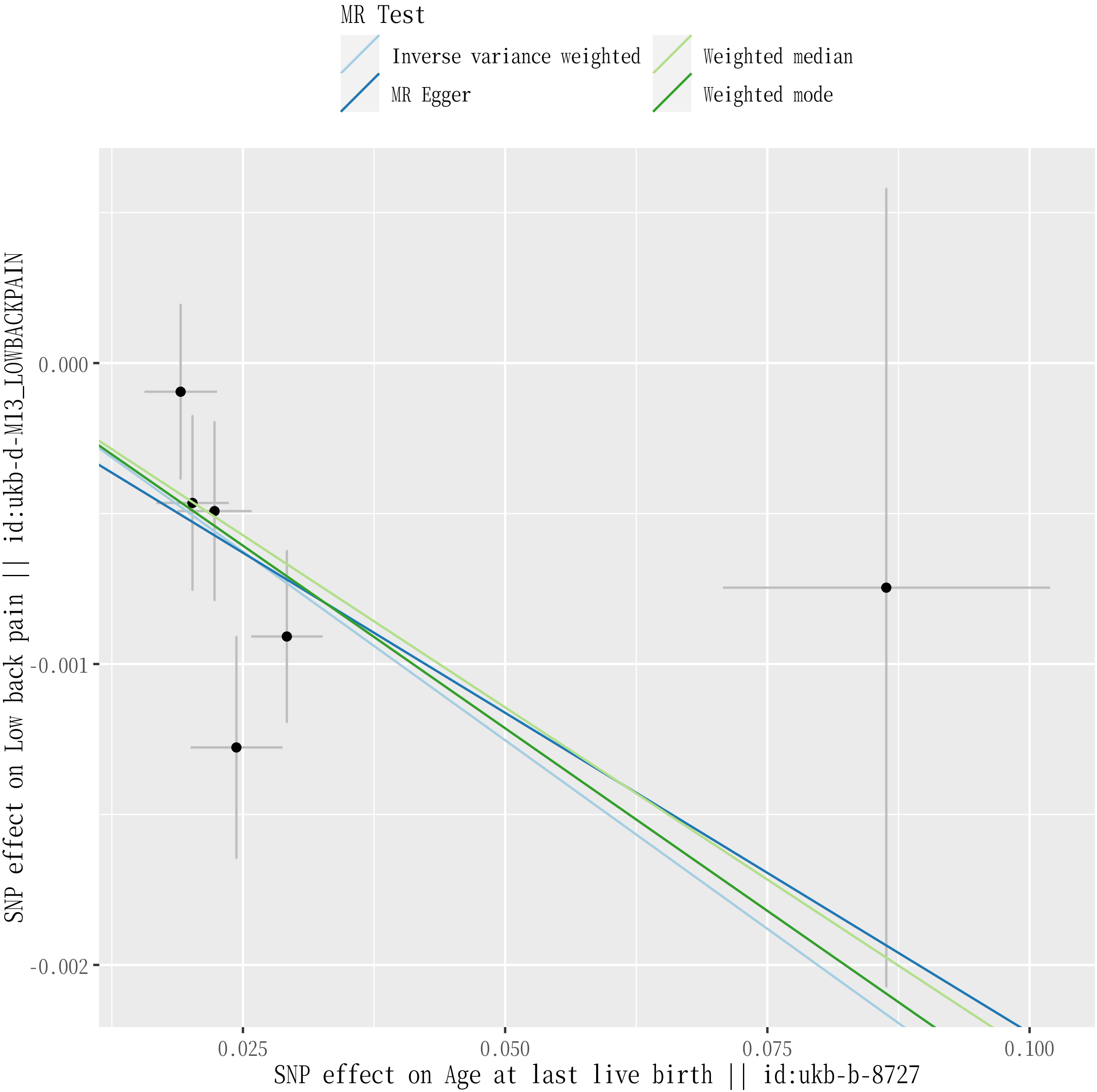
MR Test
Inverse variance weighted
MR Egger
Weighted median
Weighted mode
0.000
-0.001
-0.002
0.025
0.050
0.075
0.100
SNP effect on Age at last live birth || id:ukb-b-8727
SNP effect on Low back pain || id:ukb-d-M13_LOWBACKPAIN

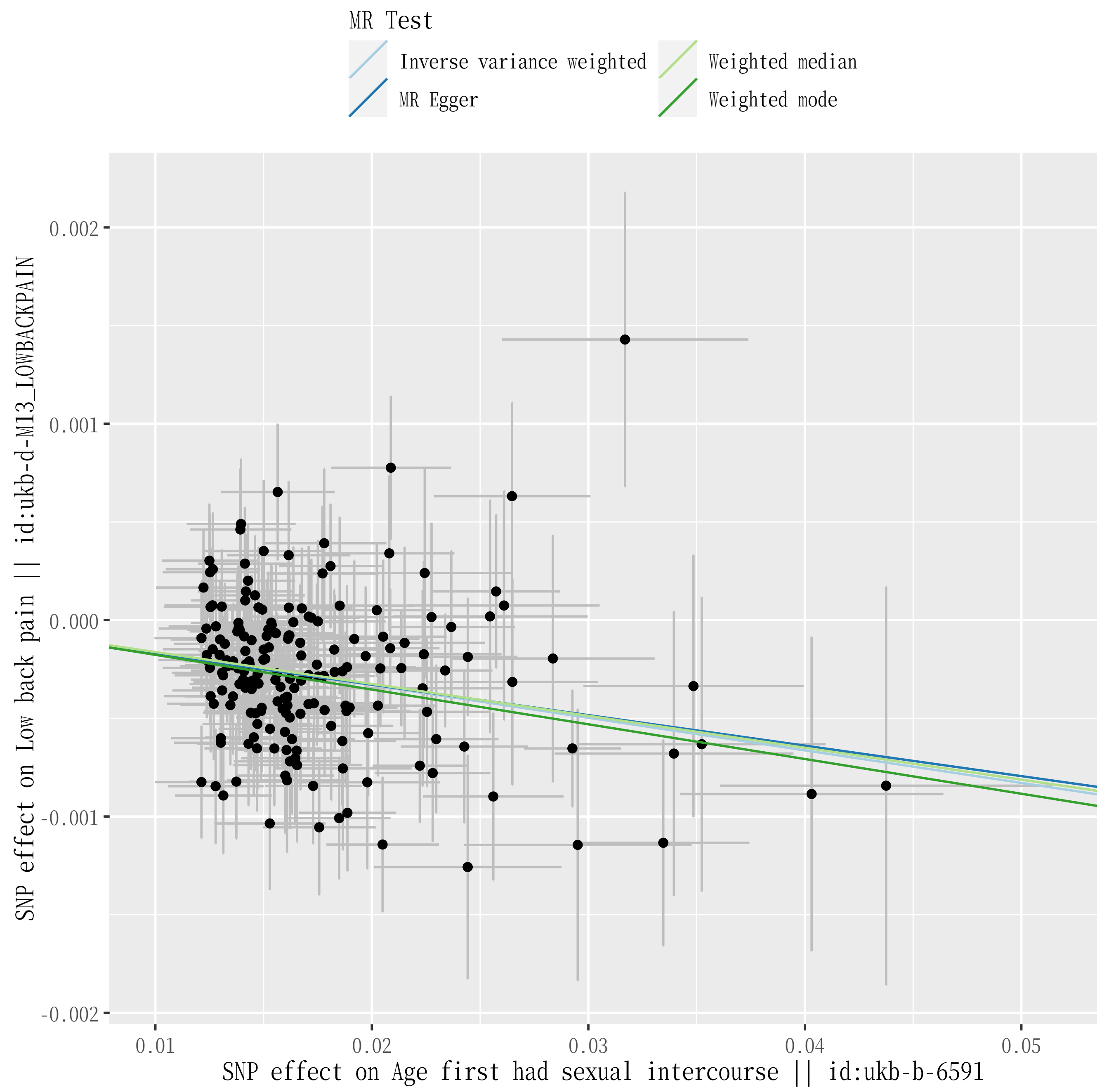
MR Test
Inverse variance weighted
MR Egger
Weighted median
Weighted mode
0.002
0.001
0.000
-0.001
-0.002
SNP effect on Low back pain || id:ukb-d-M13_LOWBACKPAIN
0.01
0.02
0.03
0.04
0.05
SNP effect on Age first had sexual intercourse || id:ukb-b-6591

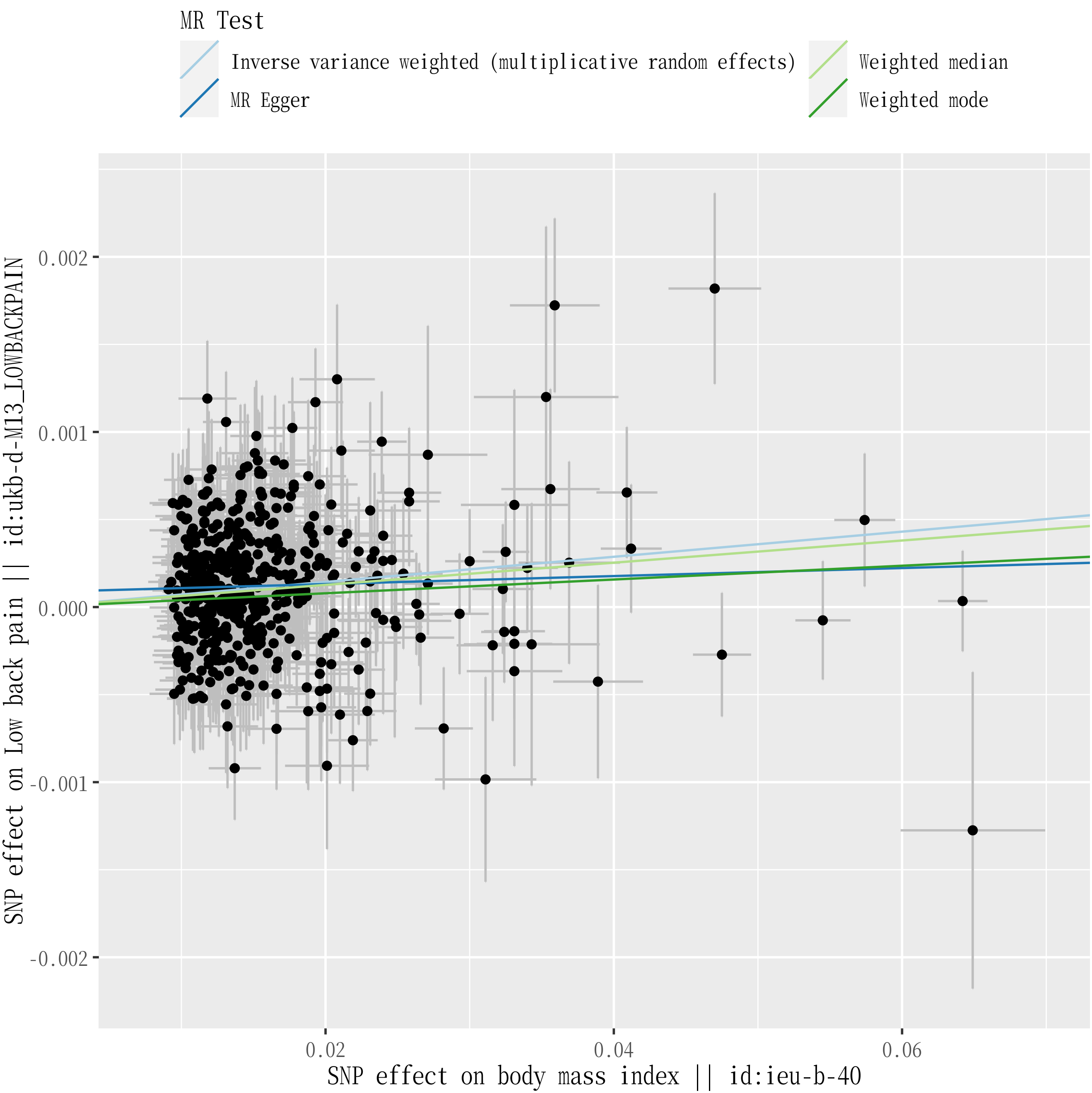

MR Test
Inverse variance weighted (multiplicative random effects)
MR Egger
Weighted median
Weighted mode
0.002
0.001
0.000
-0.001
-0.002
SNP effect on Low back pain || id:ukb-d-M13_LOWBACKPAIN
0.02
0.04
0.06
SNP effect on body mass index || id:ieu-b-40
