# Supplementary material for: A causal examination of the correlation between hormonal and reproductive factors and low back pain
Source: Front Endocrinol (Lausanne). 2024 May 10;15:1326761. doi: 10.3389/fendo.2024.1326761 (PMC11116661; doi:10.3389/fendo.2024.1326761)
Supplement: Supplementary Figure S8 — Funnel plots for LBP (replication analysis). [file DataSheet_8.pdf]

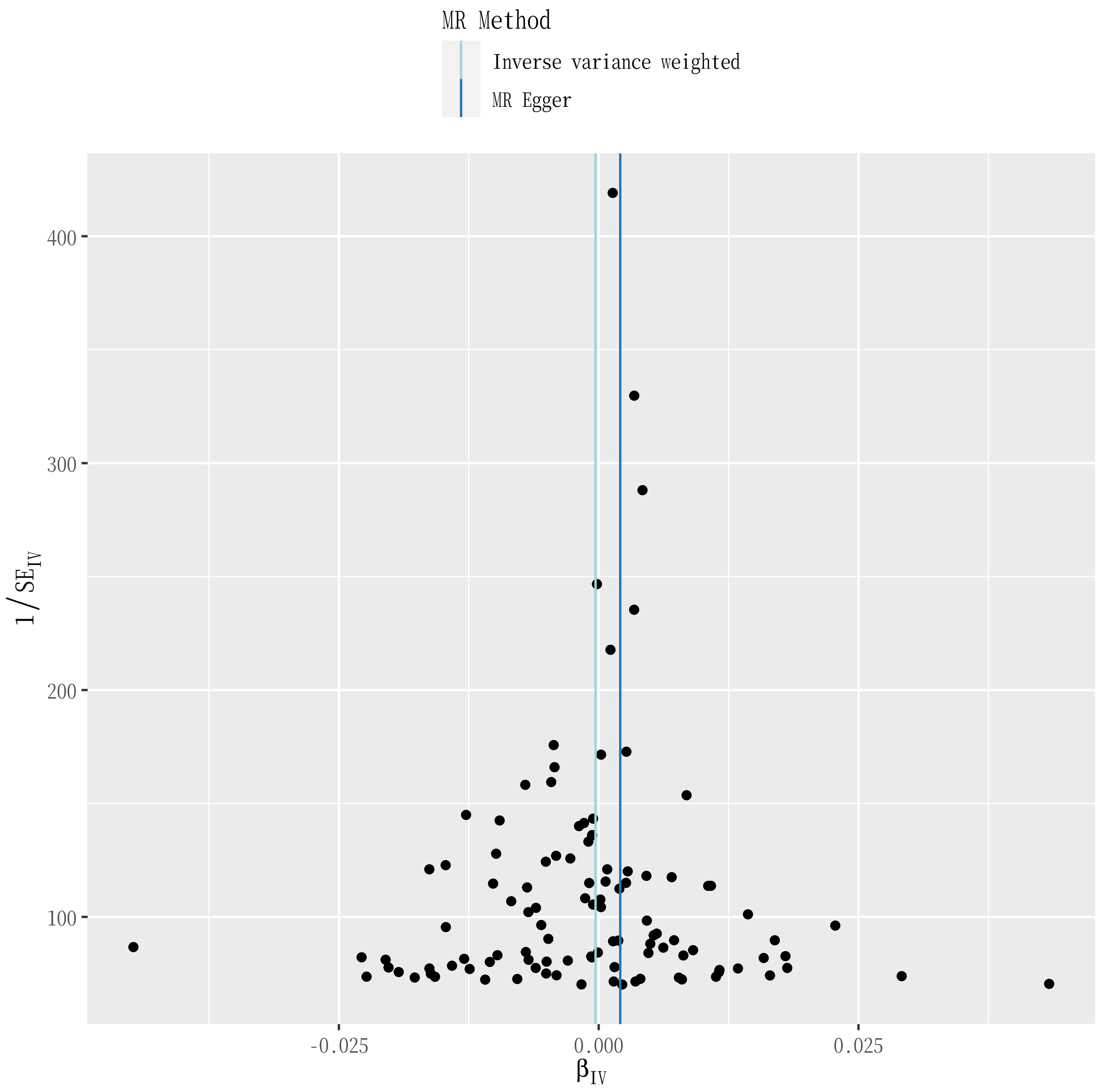

MR Method
Inverse variance weighted
MR Egger
400
300
200
100
-0.025
0.000
0.025
1/SE_IV
β_IV

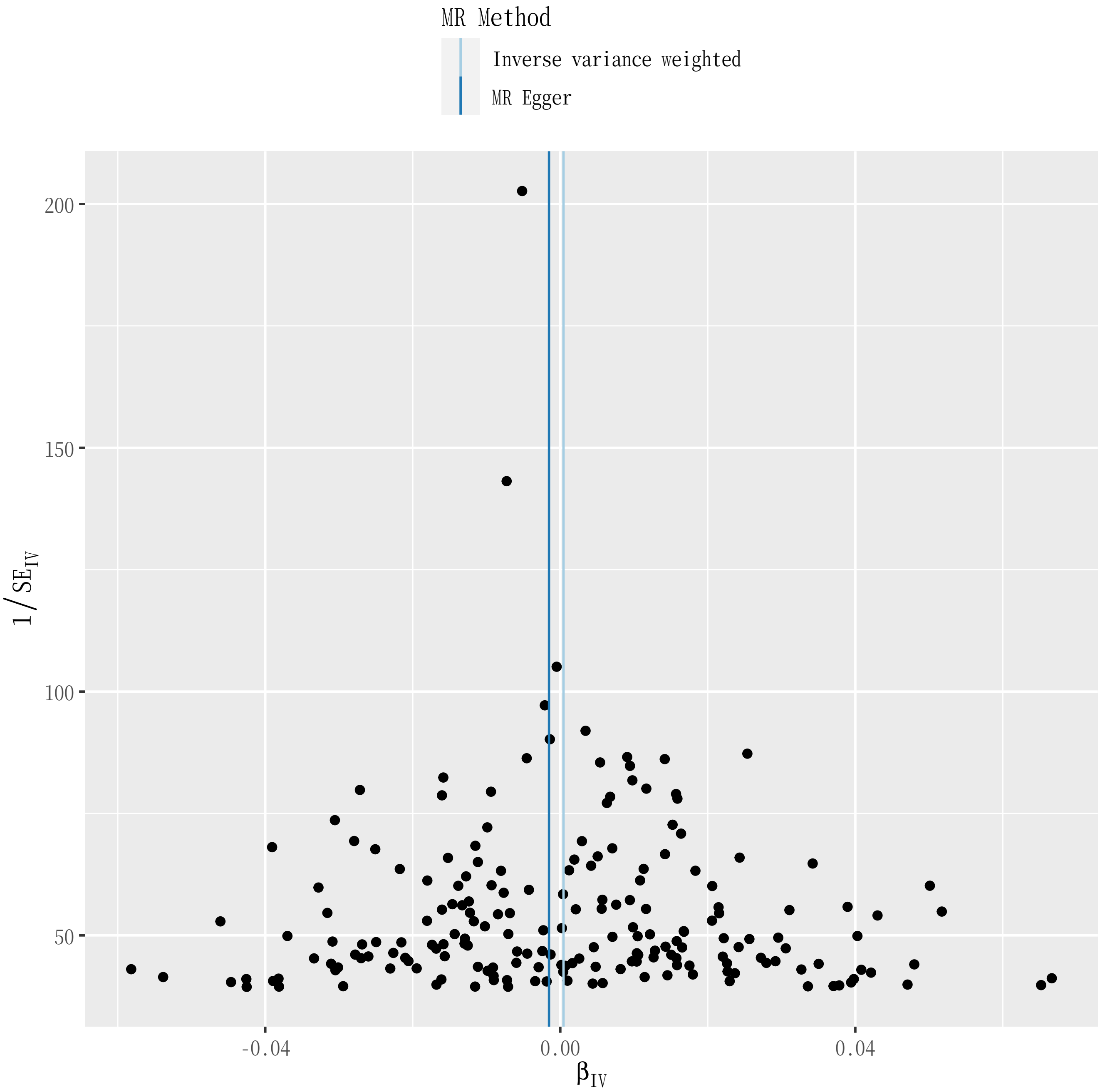
MR Method
Inverse variance weighted
MR Egger
200
150
100
50
1/SE_IV
-0.04
0.00
0.04
β_IV

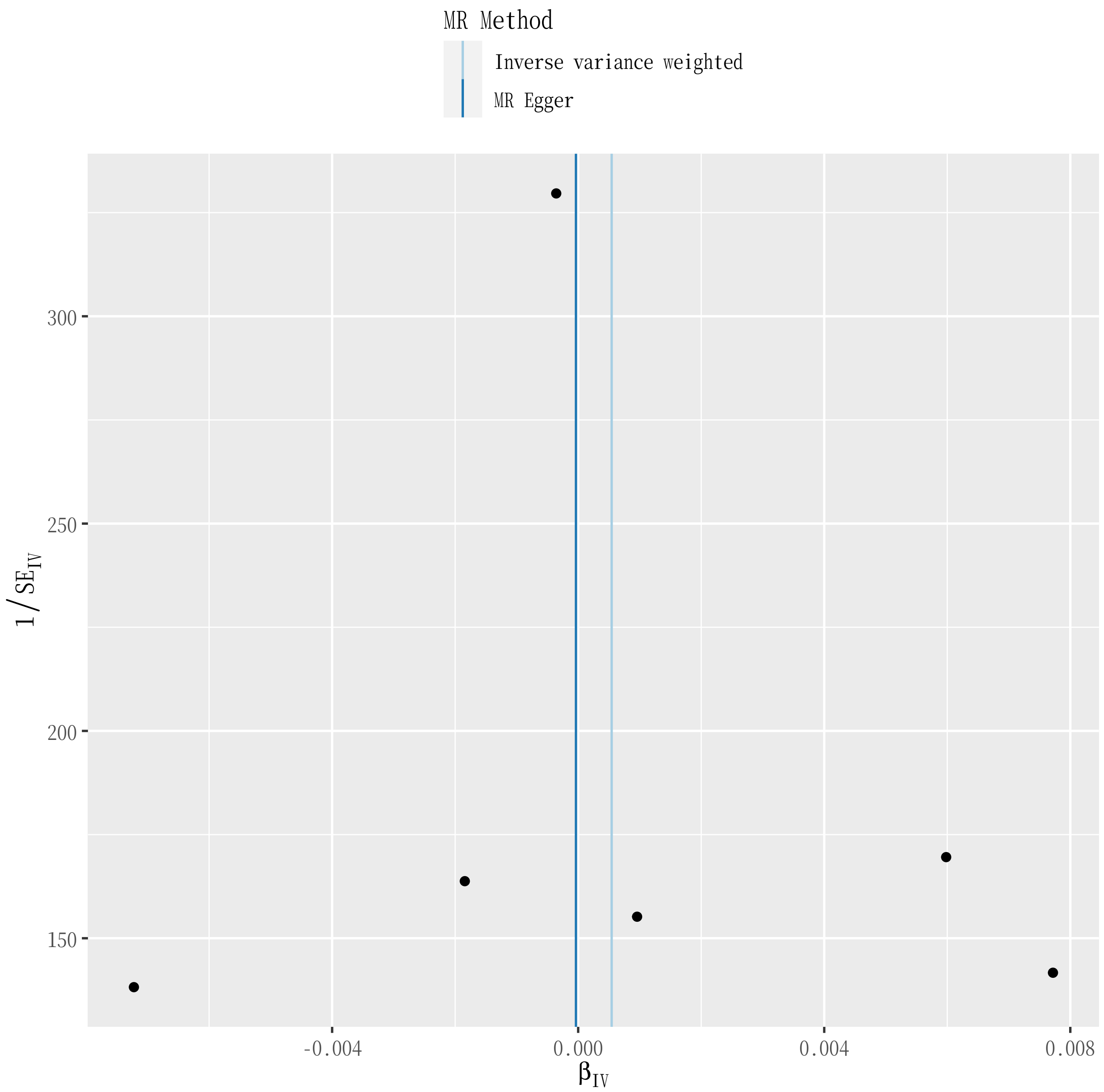
MR Method
Inverse variance weighted
MR Egger
300
250
200
150
1/SE_IV
-0.004
0.000
0.004
0.008
β_IV

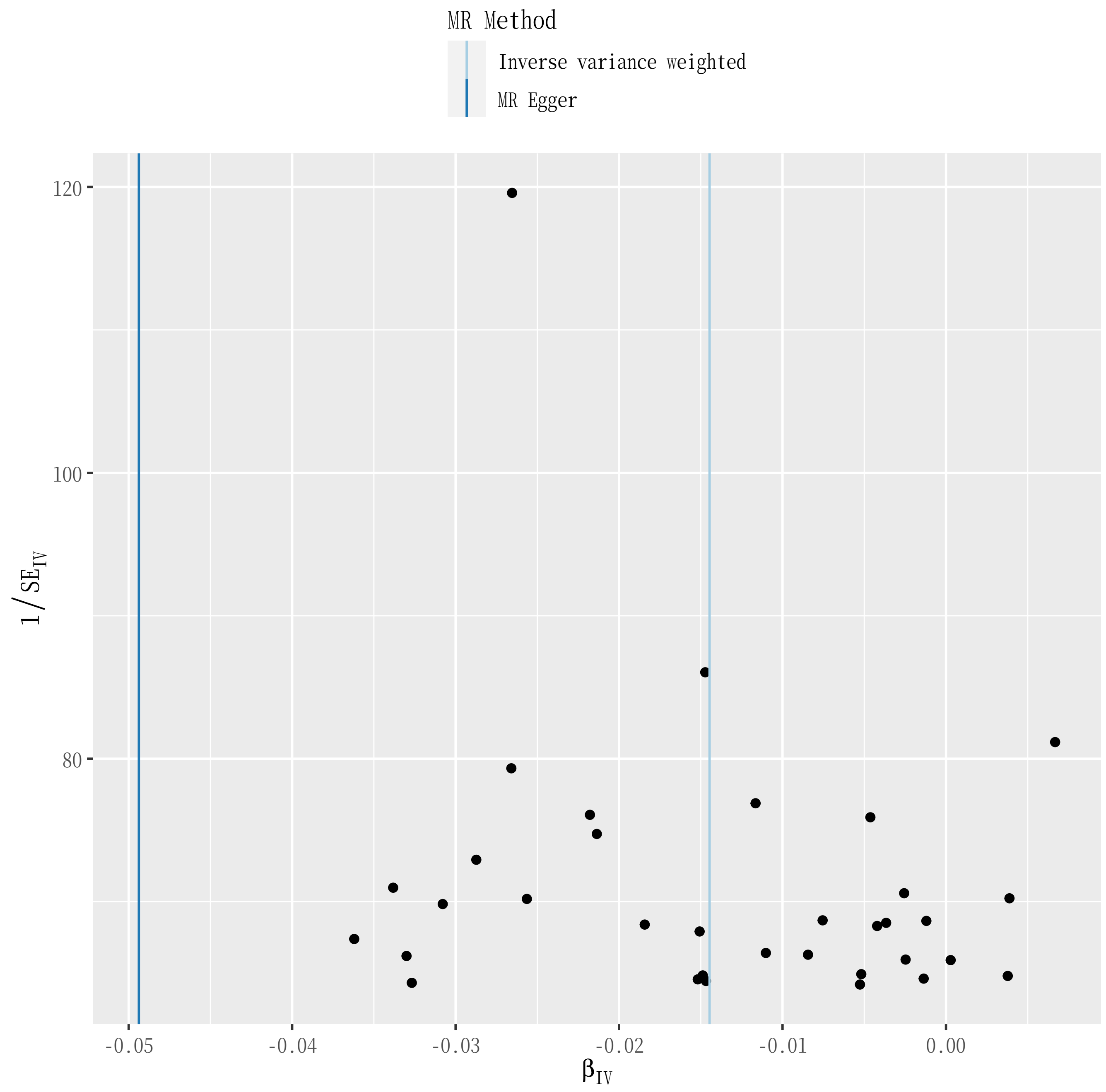

MR Method
Inverse variance weighted
MR Egger
120
100
80
1/SE$_{IV}$
-0.05
-0.04
-0.03
-0.02
-0.01
0.00
β$_{IV}$

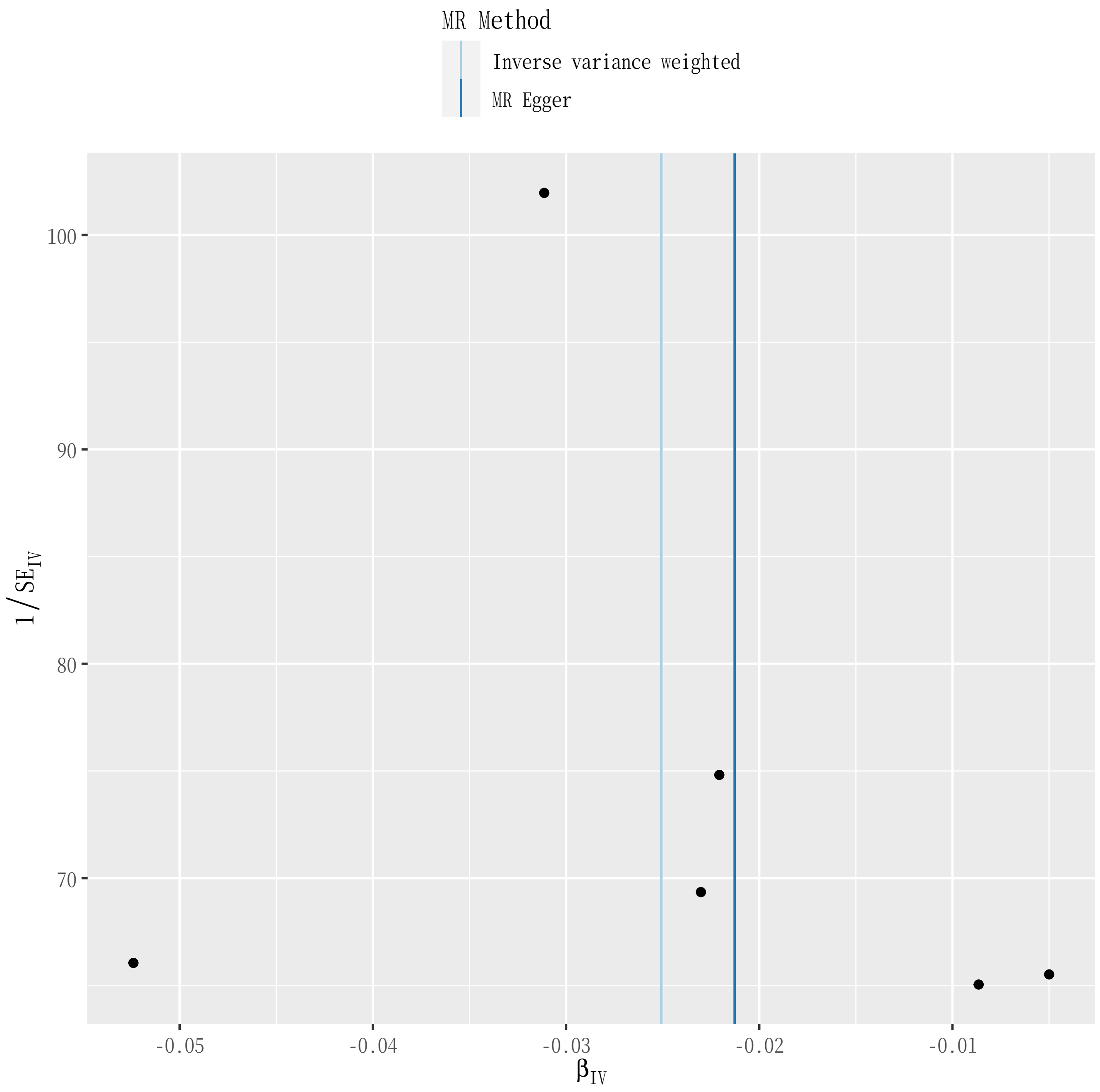

MR Method
Inverse variance weighted
MR Egger
100
90
80
70
$1/SE_{IV}$
-0.05
-0.04
-0.03
-0.02
-0.01
β_{IV}

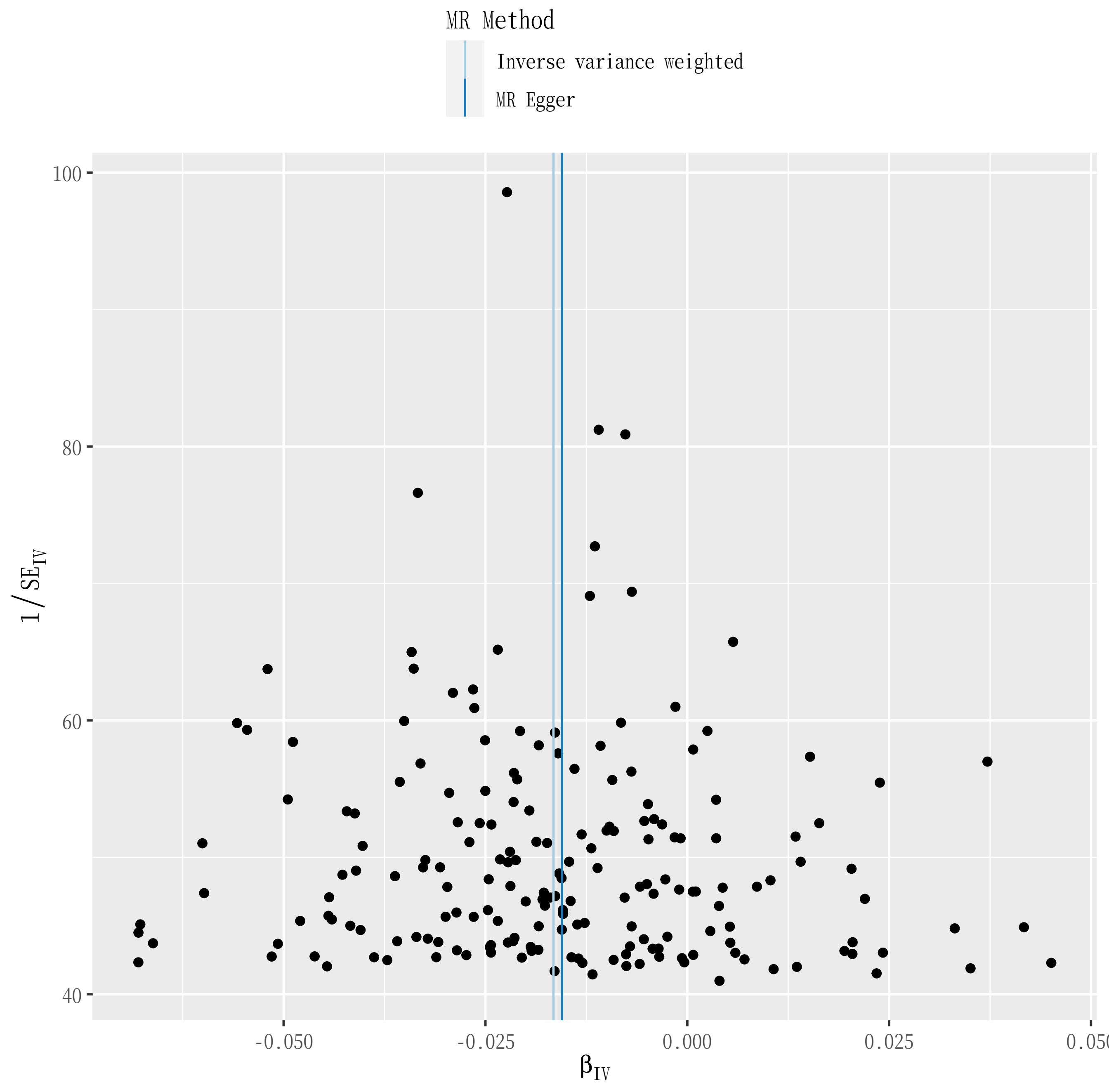

MR Method
Inverse variance weighted
MR Egger
100
80
60
40
1/SE_IV
-0.050
-0.025
0.000
0.025
0.050
β_IV

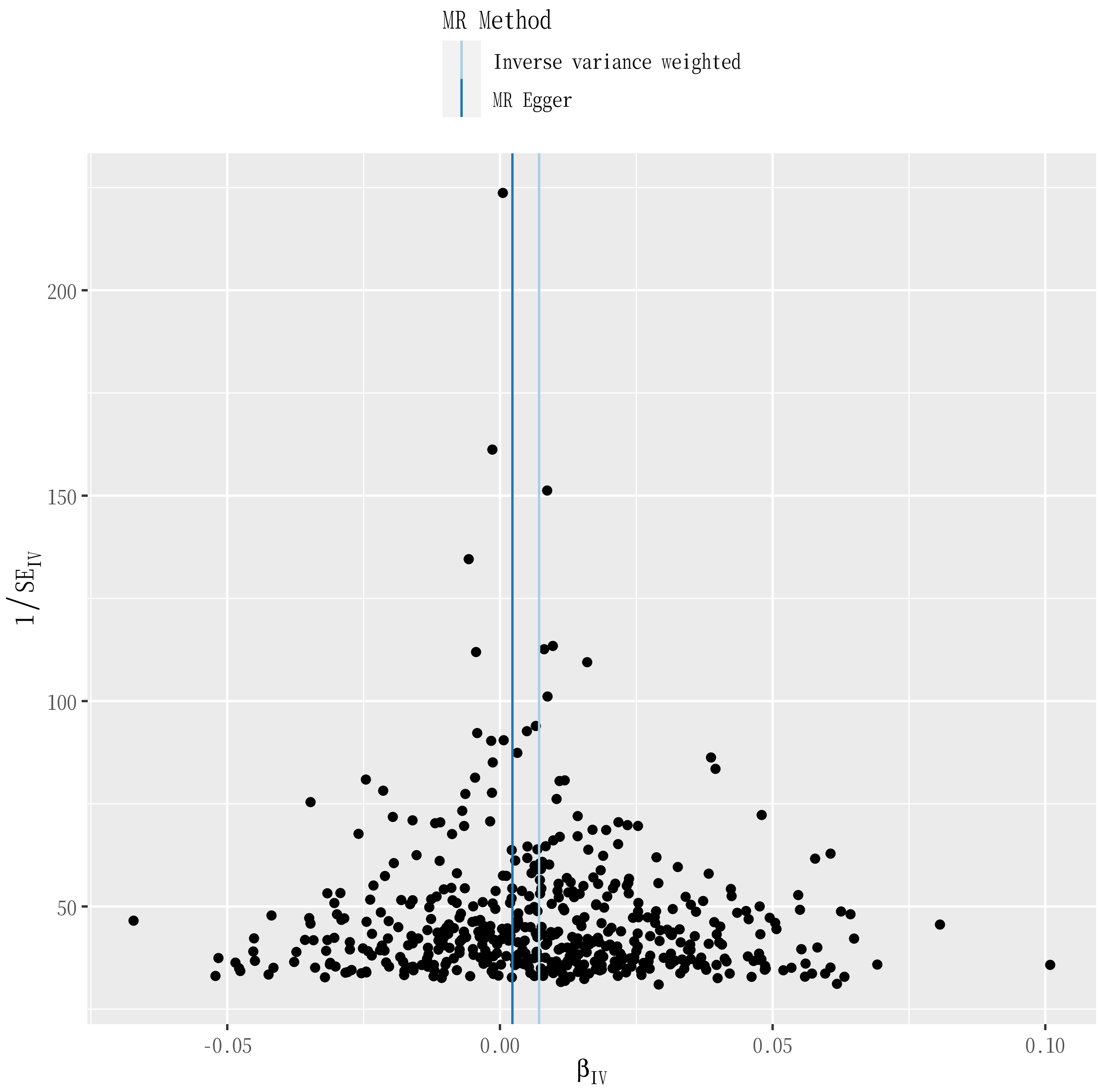
MR Method
Inverse variance weighted
MR Egger
1/SE_{IV}
200
150
100
50
-0.05
0.00
0.05
0.10
β_{IV}
