# Supplementary material for: A causal examination of the correlation between hormonal and reproductive factors and low back pain
Source: Front Endocrinol (Lausanne). 2024 May 10;15:1326761. doi: 10.3389/fendo.2024.1326761 (PMC11116661; doi:10.3389/fendo.2024.1326761)
Supplement: Supplementary Figure S9 — MR leave-one-out sensitivity analysis (replication analysis). [file DataSheet_9.pdf]

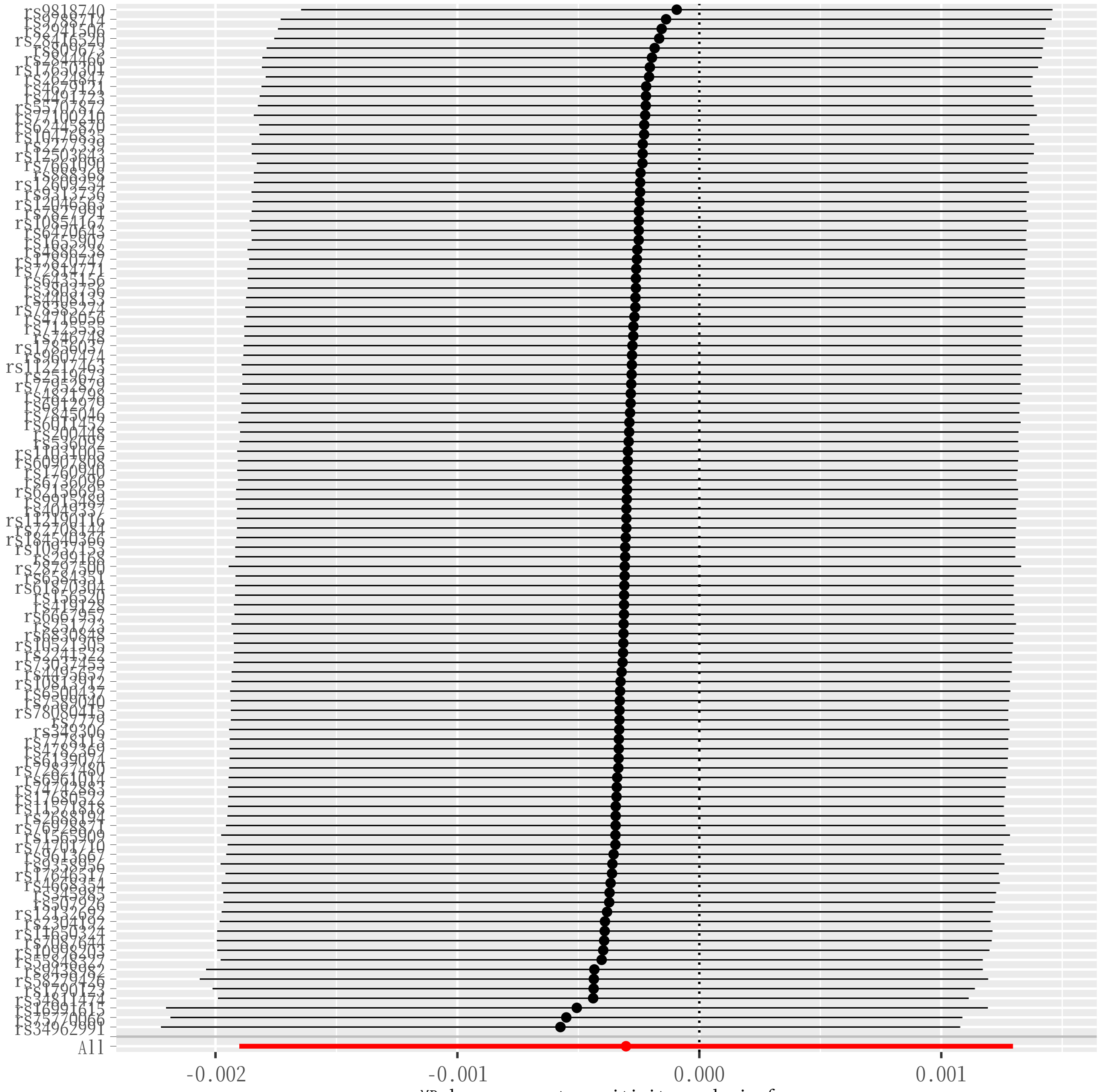

MR leave-one-out sensitivity analysis for
'Age at menopause (last menstrual period) || id:ukb-b-17422' on 'Low back pain || id:ukb-d-M13_LOWBACKPAIN'

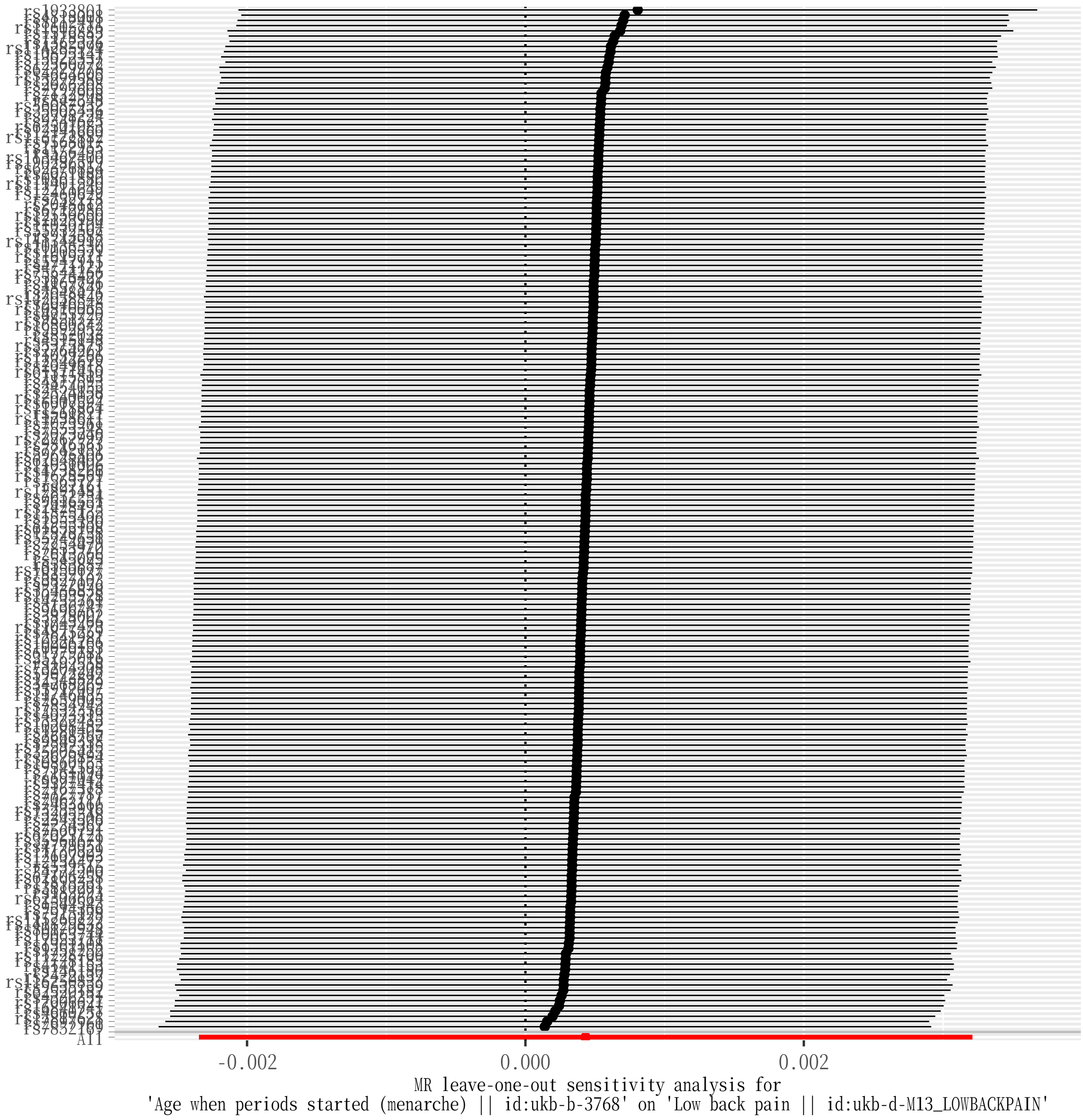
All
-0.002
0.000
0.002
MR leave-one-out sensitivity analysis for
'Age when periods started (menarche) || id:ukb-b-3768' on 'Low back pain || id:ukb-d-M13_LOWBACKPAIN'

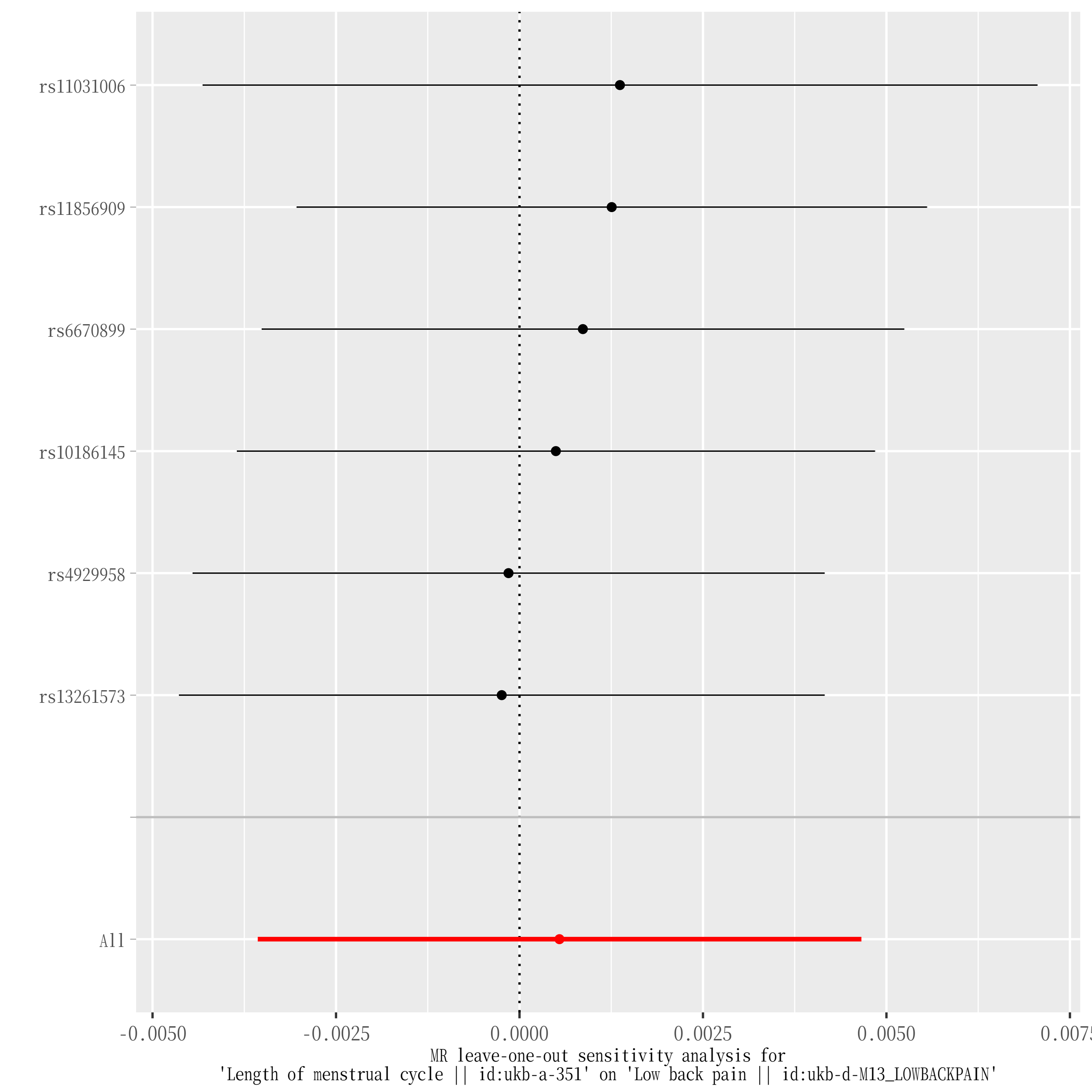
rs11031006
rs11856909
rs6670899
rs10186145
rs4929958
rs13261573
All
-0.0050
-0.0025
0.0000
0.0025
0.0050
MR leave-one-out sensitivity analysis for
'Length of menstrual cycle || id:ukb-a-351' on 'Low back pain || id:ukb-d-M13_LOWBACKPAIN'

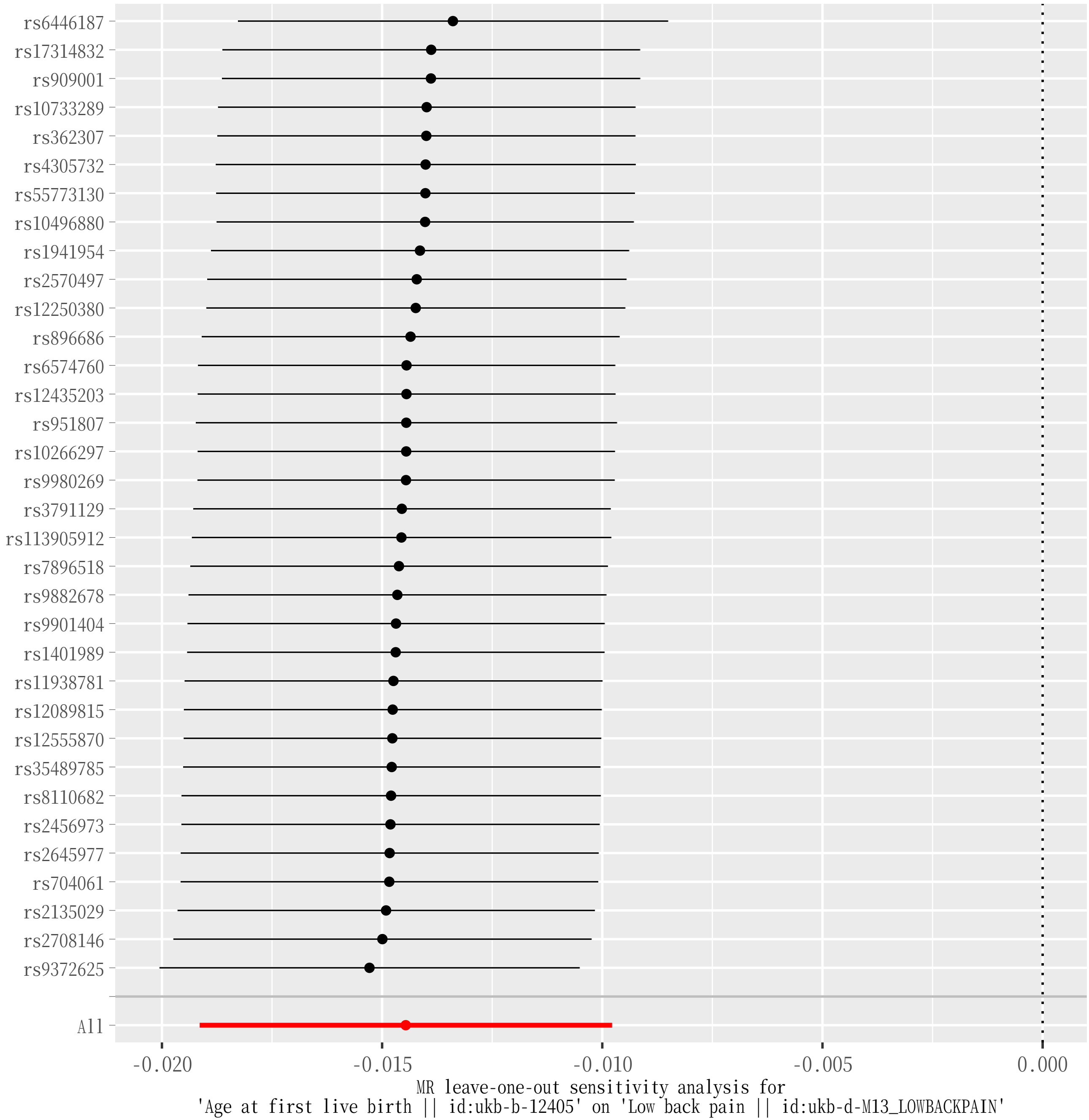

rs6446187
rs17314832
rs909001
rs10733289
rs362307
rs4305732
rs55773130
rs10496880
rs1941954
rs2570497
rs12250380
rs896686
rs6574760
rs12435203
rs951807
rs10266297
rs9980269
rs3791129
rs113905912
rs7896518
rs9882678
rs9901404
rs1401989
rs11938781
rs12089815
rs12555870
rs35489785
rs8110682
rs2456973
rs2645977
rs704061
rs2135029
rs2708146
rs9372625
All
-0.020
-0.015
-0.010
-0.005
0.000
MR leave-one-out sensitivity analysis for
'Age at first live birth || id:ukb-b-12405' on 'Low back pain || id:ukb-d-M13_LOWBACKPAIN'

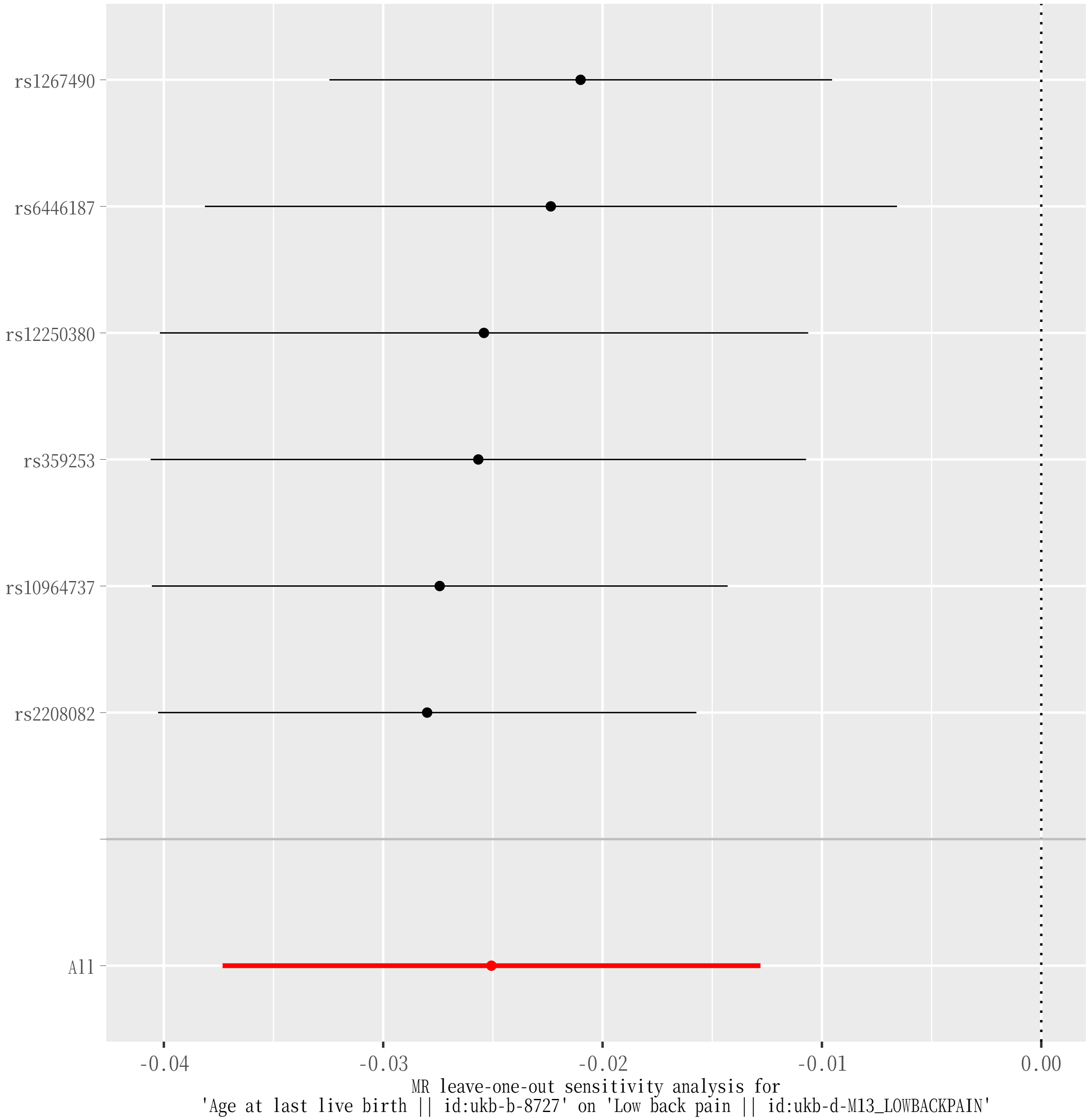
rs1267490
rs6446187
rs12250380
rs359253
rs10964737
rs2208082
All
-0.04
-0.03
-0.02
-0.01
0.00
MR leave-one-out sensitivity analysis for
'Age at last live birth || id:ukb-b-8727' on 'Low back pain || id:ukb-d-M13_LOWBACKPAIN'

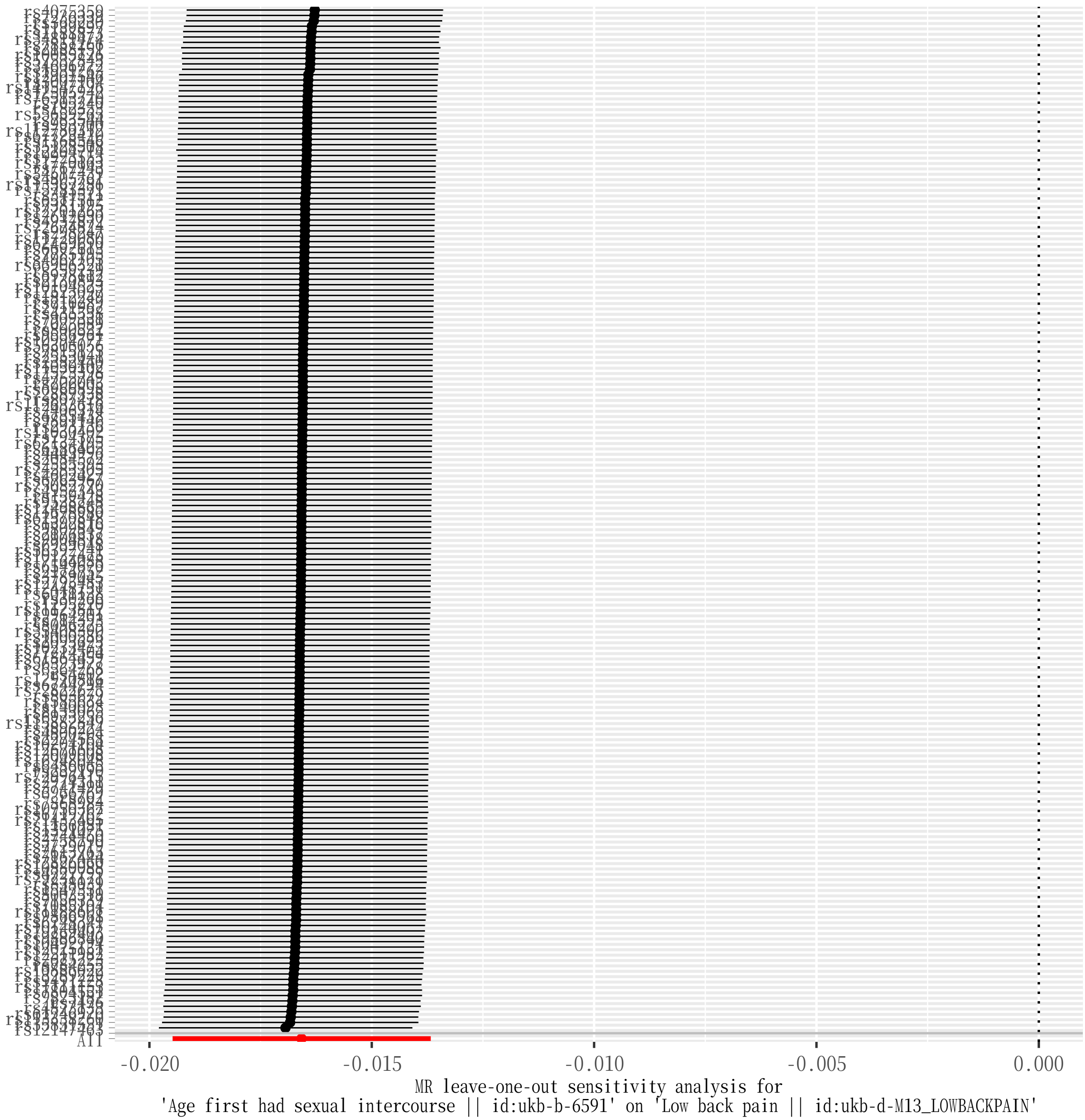
All
-0.020
-0.015
-0.010
-0.005
0.000
MR leave-one-out sensitivity analysis for
'Age first had sexual intercourse || id:ukb-b-6591' on 'Low back pain || id:ukb-d-M13_LOWBACKPAIN'

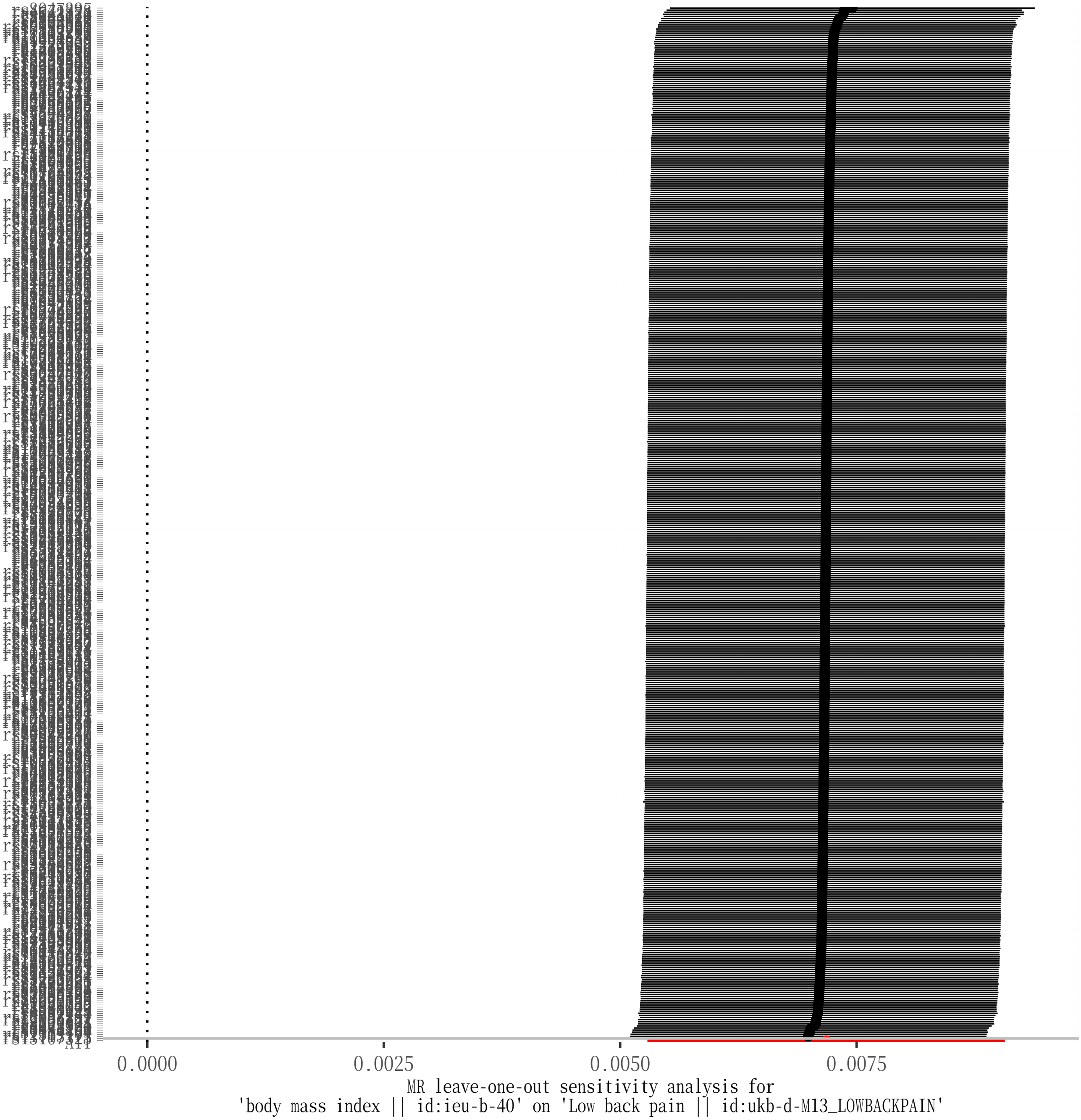
All
0.0000
0.0025
0.0050
0.0075
MR leave-one-out sensitivity analysis for
'body mass index || id:ieu-b-40' on 'Low back pain || id:ukb-d-M13_LOWBACKPAIN'
